# An adenine/thymidine-rich region is integral to RepL-mediated DNA replication

**DOI:** 10.3389/fmicb.2023.1095671

**Published:** 2023-02-09

**Authors:** Yang Wei Huan, Russell Brown, Baojun Wang

**Affiliations:** ^1^School of Biological Sciences, University of Edinburgh, Edinburgh, United Kingdom; ^2^College of Chemical and Biological Engineering & ZJU-Hangzhou Global Scientific and Technological Innovation Center, Zhejiang University, Hangzhou, China; ^3^Research Center for Biological Computation, Zhejiang Laboratory, Hangzhou, China

**Keywords:** RepL, bacteriophage P1, DNA replication, plasmid copy number, biosensor signal amplification

## Abstract

The lytic replication of bacteriophage P1 requires RepL expression and the lytic stage origin, oriL, which is postulated to be located within *repL* gene sequence. The exact sequence of P1 oriL and the mechanism(s) of RepL-mediated DNA replication, however, are not fully understood. By using *repL* gene expression to induce DNA replication of a *gfp* and a *rfp* reporter plasmids, we demonstrated that synonymous base substitution in an adenine/thymidine-rich region of *repL* gene sequence, termed AT2, significantly inhibited the RepL-mediated signal amplification. Contrastingly, mutations in an IHF and two DnaA binding sites did not affect the RepL-mediated signal amplification significantly. A truncated *repL* sequence with the AT2 region allowed RepL-mediated signal amplification *in trans* therefore verifying a significant role of the AT2 region in RepL-mediated DNA replication. A combination of *repL* gene expression and a non-protein-coding copy of *repL* gene sequence (termed nc-*repL*) was able to amplify the output of an arsenic biosensor. Furthermore, mutation(s) at single or multiple positions within the AT2 region produced varying levels of RepL-mediated signal amplification. Overall, our results provide novel insights into the identity and location of P1 oriL as well as demonstrating the potential of using *repL* constructs to amplify and modulate the output of genetic biosensors.

## Introduction

Bacteriophage P1 is a temperate phage which can lysogenise in its host cell as well as switching to a lytic lifecycle for the production of phage progeny (Yarmolinsky and Sternberg, 1988). The decision making between the P1 lysogenic and lytic stage lifecycle is determined by a multitude of environmental cues and its immunity circuitry, which forms a complex network regulating the levels of a C1 repressor and its antagonist, Coi (Austin and Abeles, 1983; Hansen, 1989; Heinrich et al., 1995a). P1 has two replicons, one being the prophage replicon containing an origin of replication known as oriR, which maintains the genome as a low copy number plasmid during the lysogenic cycle, and a separate lytic replicon containing the lytic stage origin of replication (oriL), at which DNA replication is initiated during the lytic lifecycle (Segev et al., 1980; Austin and Abeles, 1983; Walker and Walker, 1983; Hansen, 1989; Sternberg and Cohen, 1989; Cohen et al., 1996). The lytic replicon of P1 contains two protein-coding genes termed *kilA* and *repL*, which has their gene expression regulated by a promoter termed P53, and potentially by two other predicted promoters termed P*_kilA_* and P*_repL_* (Cohen and Sternberg, 1989; Hansen, 1989; Sternberg and Cohen, 1989; Łobocka et al., 2004). Transcription from the P53 promoter is inhibited by the binding of C1 repressor to an operator sequence, termed Op53, which overlaps the promoter therefore inhibiting *kilA* and *repL* gene expression during lysogenic phase (Eliason and Sternberg, 1987; Velleman et al., 1987). The function of *kilA* gene product is not known but it is not essential for P1 lytic replication yet lethal when expressed in *Escherichia coli* cells (Sternberg and Cohen, 1989; Heinrich et al., 1995b). Contrastingly, transcription of the distal *repL* gene is essential for P1 lytic stage DNA replication (Hansen, 1989; Sternberg and Cohen, 1989; Heinrich et al., 1995b; Cohen et al., 1996).

The exact location and sequence of P1 oriL is not known. An analysis of P1 genomic sequence revealed a sharp change in GC skewness at the 3′-region of *repL* gene, which coincides with two DnaA binding sites and a preceding IHF binding motif thus indicating that this region might contain the oriL sequence (Lobry, 1996; Łobocka et al., 2004). During the P1 lytic lifecycle, DNA replication initiates bidirectionally at the oriL in a theta mode (θ), followed by a predominant rolling-circle replication mode (σ) at later stages of the lytic lifecycle (Cohen, 1983). While the θ mode of DNA replication is most likely to initiate at the predicted oriL within *repL* gene sequence, the rolling-circle replication might initiate at other sites (Łobocka et al., 2004). Another possible location for an alternative and/or additional oriL lies within the *rlf* operon located downstream of *repL*, which contains two predicted IHF binding sites (Łobocka et al., 2004). Alternatively, the rolling-circle replication could potentially initiate from DNA nick introduced by P1 DNA packaging enzyme (pacase) at *pac*, which is the canonical P1 DNA packaging signal (Sternberg and Coulby, 1990).

The *repL* gene product was postulated to initiate DNA replication at the oriL, which lies within its coding sequence (Hansen, 1989; Heinrich et al., 1995b; Cohen et al., 1996). Therefore, induction of *repL* gene expression would increase the gene copy number *in cis*, which could potentially be adapted to increase the copy number of plasmids. An early study by Nat Sternberg (1990) demonstrated that isopropyl-β-d-1-thiogalactopyranoside (IPTG) induction of a *lac*-regulated P1 lytic replicon could amplify the copy number of a plasmid encoding the construct. In more recent studies, the induction of *repL* gene expression was demonstrated to amplify the copy number of plasmids thus indicating that the *cis*-acting, RepL-mediated DNA replication could be reproduced in *Escherichia coli* without the need of other P1-derived factors (Sheth et al., 2017; Dwidar and Yokobayashi, 2019). Furthermore, Sheth et al. (2017) demonstrated that codon optimisation of the *repL* gene sequence reduced the *cis*-acting, DNA replication while the protein product could amplify the copy number of another plasmid bearing a non-protein coding *repL* sequence *in trans*. Therefore, RepL expression can be decoupled from the *cis*-acting plasmid amplifying effect, by introducing mutations to disrupt the oriL which is predicted to be located within *repL* gene sequence (Sheth et al., 2017). Consistently, Li et al. (2023) demonstrated that RepL expression could increase the copy number of plasmids, which amplified the signal of an *E. coli*-based As(III) biosensor therefore indicating the potential use of such construct as a signal amplifier for *E. coli* cell-based genetic circuits.

While the use of RepL-mediated DNA replication to increase plasmid copy number *in cis* and *in trans* were demonstrated previously, the identity of P1 *oriL*, molecular mechanisms behind RepL-mediated DNA replication remained unknown. We hypothesized that region(s) within the *repL* gene sequence which is important for RepL-mediated DNA replication would be representative of the P1 *oriL*. Therefore, we have generated a *gfp* reporter plasmid providing P_BAD_ regulated-*repL* gene expression, which would allowed assessment of RepL protein activity in laboratory strains of *E. coli* (Coleman et al., 1977). Arabinose induction of *repL* gene expression increased the GFP fluorescence intensity thus indicating a RepL-mediated amplification of the *gfp* plasmid copy number. Moreover, induction of *repL* gene expression increased the output of a *rfp* reporter plasmid containing a non-protein coding *repL* sequence, hence demonstrating RepL-mediated DNA replication *in trans*. Synonymous base substitutions in the second half of *repL* gene revealed an adenine/thymidine-rich region (termed AT2 hereafter) which was important for RepL-signal amplification. We demonstrated that truncated *repL* sequences containing the AT2 region allowed RepL-mediated signal amplification *in trans*. Taken together, our data indicated a minimal region within *repL* gene encompassing AT2 that could serve as the target site for RepL-mediated plasmid DNA replication in *E. coli* therefore narrowing down the potential location of P1 *oriL*. Furthermore, single or a combination of synonymous mutations within the AT2 region produced varying levels of *trans*-acting, RepL-mediated signal amplification, which could be expanded to modulate output signals of genetic circuits. Consistently, our list of *repL* and nc-*repL* constructs provided amplification and modulation of a biosensor output in laboratory strain of *E. coli*, therefore justifying the potential use of our constructs in the field of synthetic biology to provide a desirable output level of signal amplification.

## Results

### A *gfp* reporter assay to identify DNA sequence(s) which are required for RepL-mediated DNA replication

**Figure 1:**
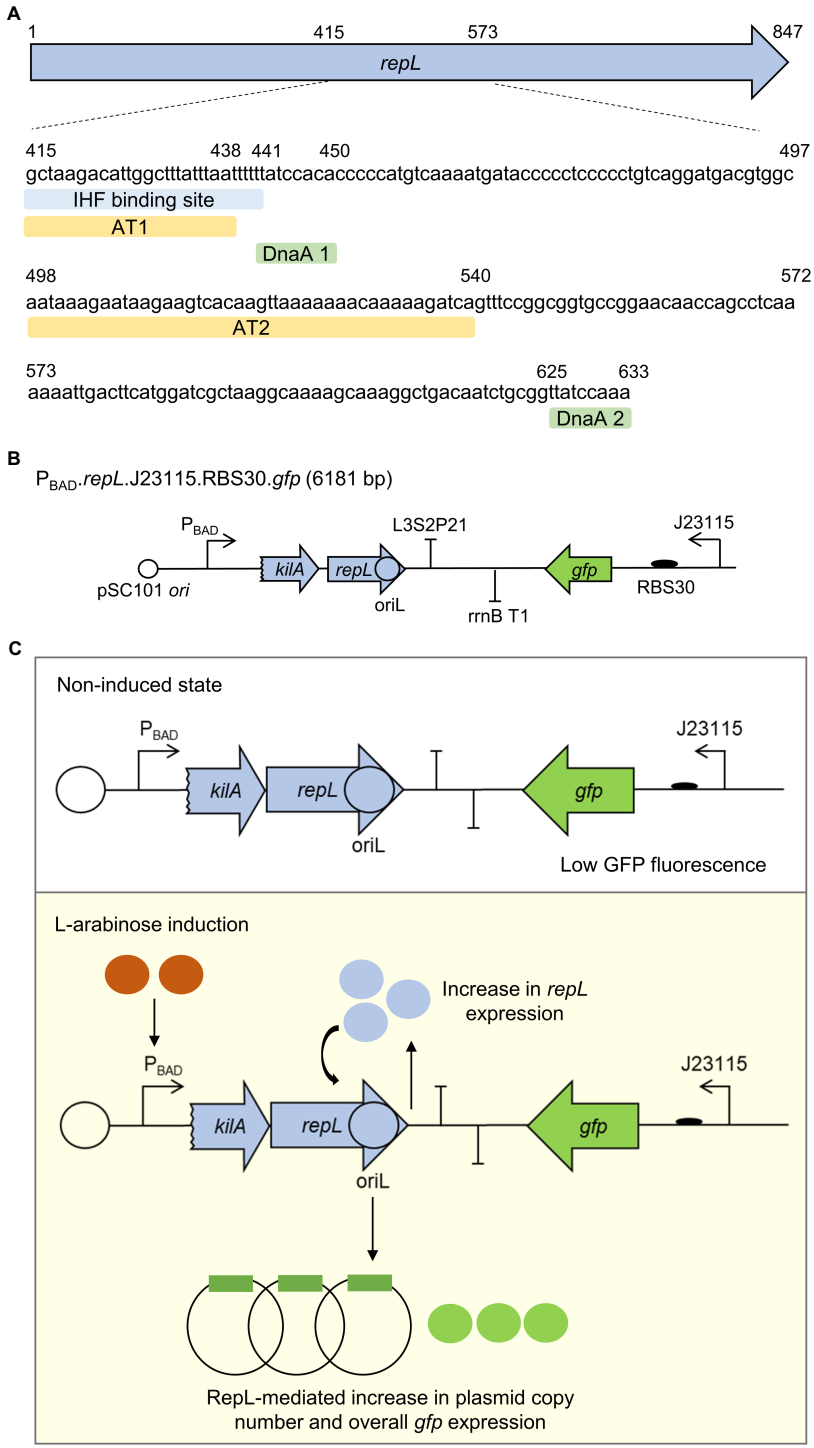
A *gfp* reporter plasmid to assess the RepL-mediated DNA replication process in *E. coli*. **(A)** The location (nucleotide positions relative to *repL* open reading frame, first nucleotide and last nucleotide of AT1, AT2, DnaA binding sites 1 and 2 were shown as numbers) and sequences of A/T-rich regions 1 and 2 (AT1, AT2, light orange), IHF binding site (light blue), DnaA binding sites 1 and 2 (DnaA 1, DnaA 2, light green) within the second half of *repL* gene (light blue arrow). The location of these DNA elements in relation to the full length *repL* coding sequence was shown in Supplementary Figure 1. **(B)** Schematic diagram of a *gfp.repL* reporter construct. *repL* (light blue arrow) gene expression was controlled by the arabinose-inducible promoter P_BAD_. A 24 bp coding sequence of *kilA* was included, as this provides the native ribosomal binding site (RBS) for *repL* translation (Sheth et al., 2017). oriL, the lytic stage origin of replication is postulated to be located in the second half of *repL* gene. *Gfp* (in green) expression is controlled by a weak constitutive promoter Bba_J23115, with an artificial ribosomal binding site, RBS30. The plasmid confers ampicillin resistance and has a pSC101 *ori* conferring low plasmid copy number. **(C)** Schematic diagram describing the RepL-mediated GFP signal amplification process. Arabinose induction of RepL expression in a non-P1 lysogenic strain of *E. coli* would promote DNA replication of the plasmid *in cis*, which was demonstrated in previous studies (Sheth et al., 2017; Dwidar and Yokobayashi, 2019). Therefore, the level of *cis*-acting, RepL-induced plasmid replication can be measured semi-quantitatively, which would be represented by the increase in GFP fluorescence intensity when compared to uninduced state.

Although the molecular mechanism of P1 lytic stage DNA replication is not known, *repL* gene product was assumed to initiate DNA replication, by targeting the oriL postulated to be located within the second half of *repL* gene sequence (Hansen, 1989; Sternberg and Cohen, 1989). We have identified two 24 bp and 43 bp adenine/thymidine-rich in regions in the second half of *repL* gene sequence termed AT1 and AT2 hereafter (~70 and ~ 80% A/T composition respectively), which are adjacent to the DnaA binding sites annotated in a previous study (Łobocka et al., 2004; Figure 1A; Supplementary Figure 1). A stretch of ~20 bp A/T-rich sequence, also known as DNA unwinding elements (DUEs), facilitates DNA denaturation at bacterial origin of replication due to its helical instability (Matsui et al., 1985; Coman and Russu, 2005; Rajewska et al., 2012). The AT1 region overlaps with a previously identified IHF binding site, which might be involved in promoting DNA replication and the assembly of other pre-replication protein complexes, similar to what was reported for the role of IHF at *E. coli* oriC (Ryan et al., 2002; Kasho et al., 2021). Furthermore, the AT2 region contains a 13-mer like motif at the 3′-end (5’-GATCTTTTTGTTT-3′), in which the consensus GATCTnTTnnTTT repeats were demonstrated to facilitate DNA duplex melting at *E. coli* oriC in the presence of host-derived factors (Bramhill and Kornberg, 1988). We therefore hypothesized that the two A/T-rich regions and the DnaA binding sites might play an important role in RepL-mediated DNA replication process. Plasmid-derived, RepL expression was demonstrated to amplify the vector copy number *in cis* (Sheth et al., 2017; Dwidar and Yokobayashi, 2019). Such vectors could be used to identify if the A/T-rich regions and/or the DnaA binding sites play an important role in RepL-mediated DNA replication. Hence, to assess the level of RepL-mediated DNA replication semi-quantitatively, we have designed a low copy number, *gfp*-encoding reporter plasmid that contains a *repL* gene controlled by the arabinose inducible promoter, P_BAD_ (Figure 1B). Arabinose induction of *repL* gene expression in *E. coli* would promote DNA replication of the reporter plasmid *in cis*. Therefore, the level of RepL activity would be represented by an increase in GFP fluorescence signal, when compared to uninduced state (Figure 1C).

### Arabinose induction of *repL* gene expression increased the output signal of a *gfp*-encoding reporter plasmid

To assess if induction of *repL* gene expression could increase the output signal of a *gfp* reporter plasmid, *E. coli* TOP10 cells were transformed with the *gfp.repL* reporter plasmid and the transformants were induced with a range of L-arabinose concentrations. The GFP fluorescence intensity as well as the OD_600_ of cells were measured over a period of 10 h, and the final output was represented as GFP fluorescence intensity per OD_600_ (Fluo./OD_600_).

**Figure 2:**
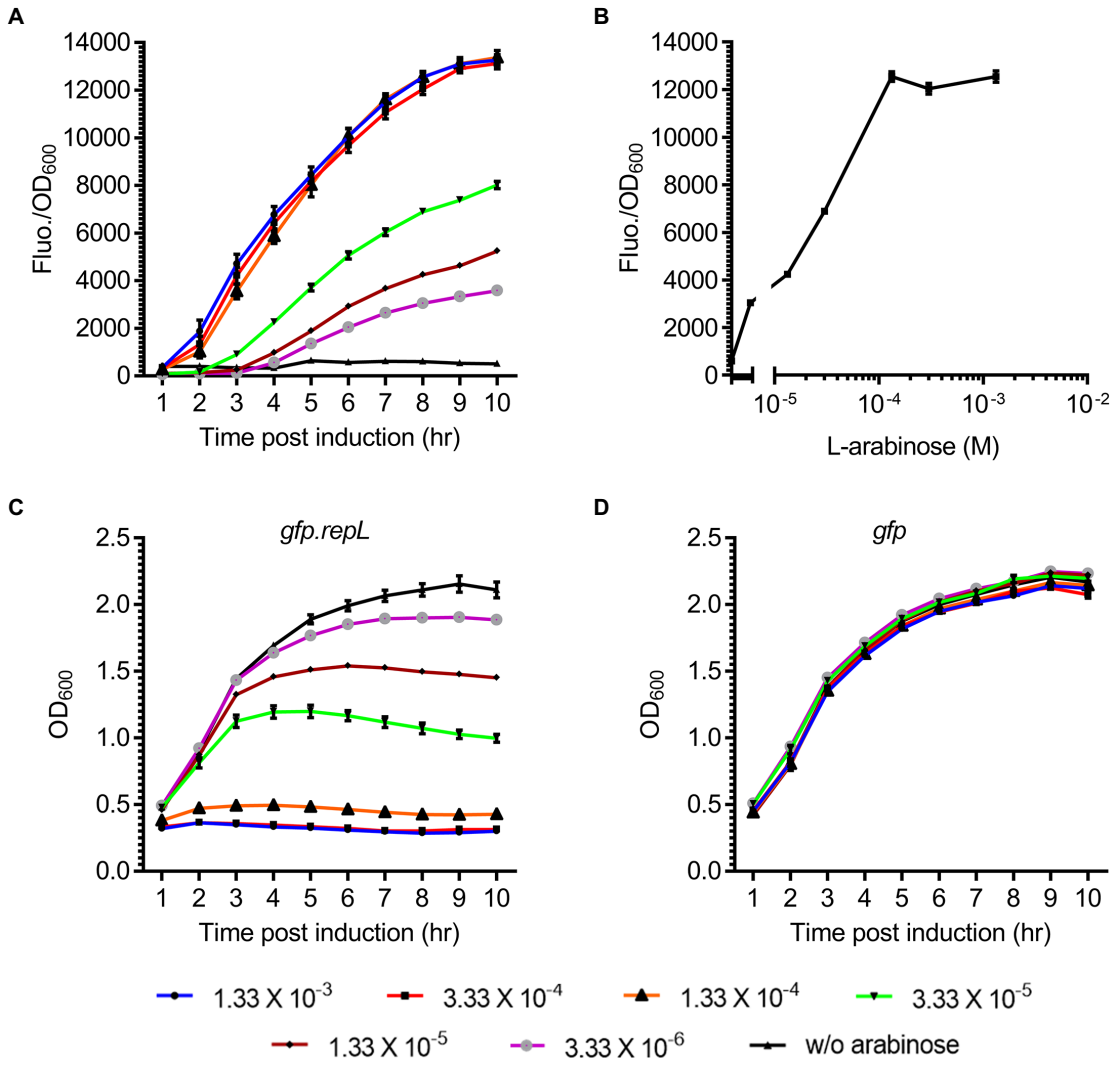
Arabinose induction of *repL* gene expression increased the GFP signal of a reporter plasmid. **(A)** The GFP fluorescence intensity per OD_600_ (FIuo./OD_600_) of *E. coli* TOP10 *gfp.repL* transformants, at 1 to 10 hpi with 3.33 × 10^−6^ M (purple line with gray circles), 1.33 × 10^−5^ M (brown line with black diamonds), 3.33 × 10^−5^ M (green line with black triangles), 1.33 × 10^−4^ M (orange line with black triangles), 3.33 × 10^−4^ M (red line with black squares), 1.33 × 10^−3^ M of L-arabinose (blue line with black circles). GFP FIuo./OD_600_ values of uninduced cells (w/o arabinose, in black line with black triangles) were added for comparison purposes. **(B)** The GFP FIuo./OD_600_ of TOP10 transformants at 8 hpi with 3.33 × 10^−6^ M, 1.33 × 10^−5^ M, 3.33 × 10^−5^ M, 1.33 × 10^−4^ M, 3.33 × 10^−4^ M, 1.33 × 10^−3^ M of L-arabinose. **(C)** The OD_600_ of TOP10 *gfp.repL* transformants and **(D)** *gfp* only transformants, at 1 to 10 h PI with 3.33 × 10^−6^ M (purple line with gray circles), 1.33 × 10^−5^ M (brown line with black diamonds), 3.33 × 10^−5^ M (green line with black triangles), 1.33 × 10^−4^ M (orange line with black triangles), 3.33 × 10^−4^ M (red line with black squares), 1.33 × 10^−3^ M of L-arabinose (blue line with black circles). OD_600_ values of uninduced cells (w/o arabinose, in black line with black triangles) were added for comparison purposes. All GFP fluorescence intensity were normalized to that of TOP10 cells transformed with an empty plasmid without *gfp* and *repL* genes. Induction assays were performed with 3 biological replicates and 4 technical replicates. Data were presented as mean ± SEM.

An increase in the GFP Fluo./OD_600_ of *gfp.repL* transformants was observed after 5 h post induction (hpi) with all concentration of arabinose, when compared to uninduced state (Figure 2A). 1.33 × 10^−4^ M was the lowest L-arabinose concentration required to give a maximum ~12-fold increase in GFP FIuo./OD_600_ values at 10 hpi, when compared to uninduced state (*p* < 0.0005). Contrastingly, arabinose induction of the *gfp* plasmid without a *repL* gene did not increase the GFP FIuo./OD_600_ values of TOP10 transformants at all time points post induction when compared to uninduced state (*p* > 0.05) (Supplementary Figures 2A,B). Furthermore, replacing the preceding 53 bp *kilA* coding sequence with a medium strength, artificial ribosomal binding site, RBS32, reduced the maximum GFP FIuo./OD_600_ value by ~9.2-fold between 5 to 10 hpi with 1.33 × 10^−3^ M and 1.33 × 10^−4^ M arabinose (*p* < 0.0005) (Supplementary Figures 3A,B). Since further increases in arabinose concentration did not improve the GFP FIuo./OD_600_ values (*p* > 0.05), 1.33 × 10^−4^ M was chosen as the optimal arabinose concentration for inducing *repL* gene expression in *E. coli* TOP10 (Figure 2B). The induction of TOP10 *gfp.repL* transformants with 1.33 × 10^−3^ M to 1.33 × 10^−4^ M arabinose yielded an average OD_600_ of ~0.5 at 8 hpi, which was significantly lower when compared to an average OD_600_ of ~2.0 recorded at uninduced state (*p* < 0.0005) (Figure 2C). On the contrary, the average OD_600_ of TOP10 *gfp* or *gfp*.RBS32-*repL* were ~ 2.0 and ~ 1.8 respectively, at 8 hpi with 1.33 × 10^−3^ M to 1.33 × 10^−4^ M arabinose (Figure 2D; Supplementary Figure 3C).

Taken together, induction of *repL* gene expression in *E. coli* TOP10 could increase the output of a *gfp* reporter plasmid, which indicated amplification of the plasmid copy number by RepL.

### A *repL^AT2^* allele significantly reduced the RepL-mediated signal amplification

**Figure 3:**
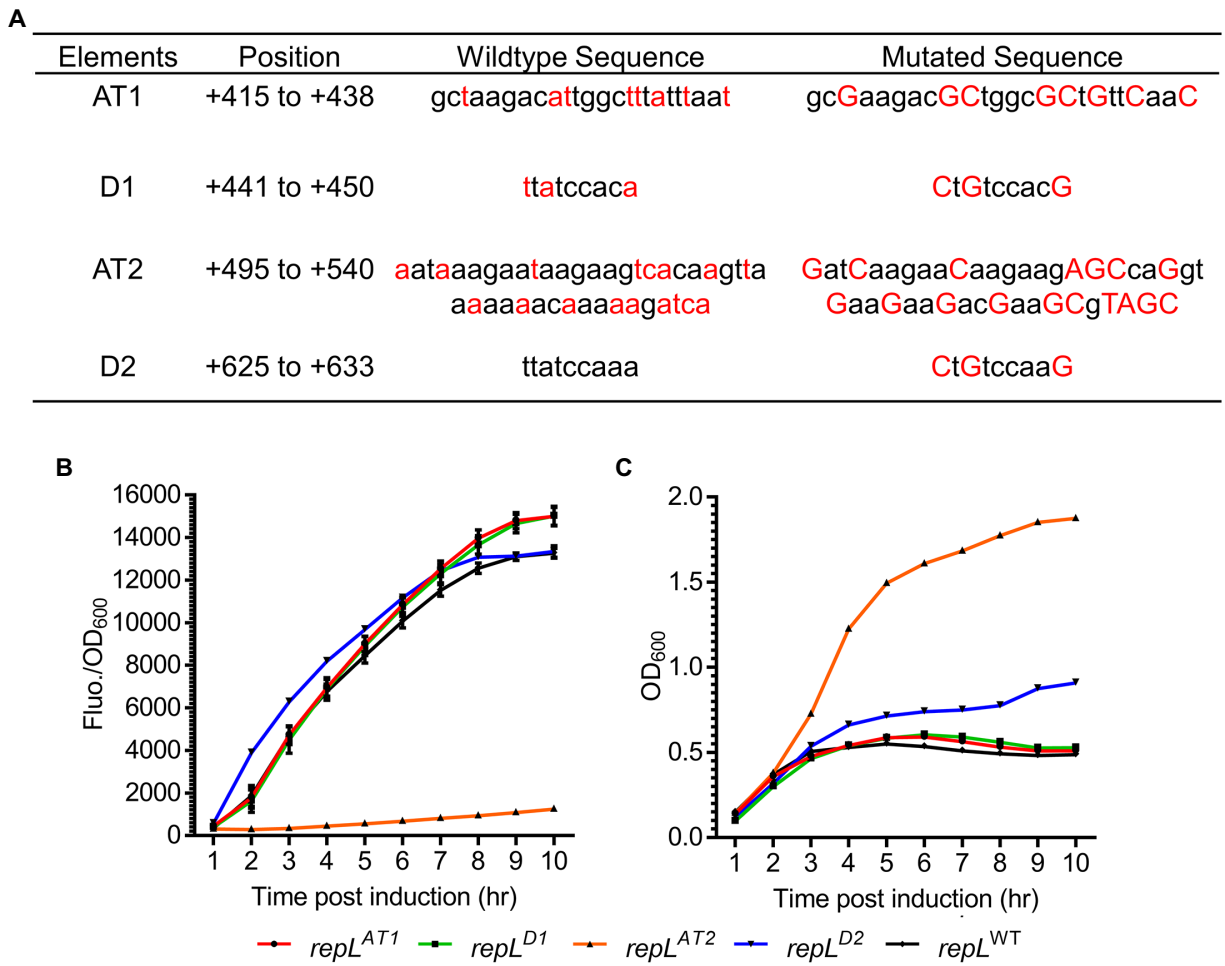
A *repL*^AT2^ allele significantly reduced the RepL-mediated GFP signal amplification. **(A)** Two A/T-rich regions termed AT1 and AT2, as well as two DnaA binding sites termed D1 and D2, might be important for RepL-mediated DNA replication. Synonymous base substitutions were introduced into a *repL* gene *via* PCR without affecting the amino acid sequence (i.e., codon-optimisation). The mutated bases (in red uppercase) as well as the wildtype sequence (in black lowercase) were shown. The position of these elements within the *repL* open reading frame (ORF) were shown. **(B)** The GFP fluorescence intensity per OD_600_ (FIuo./OD_600_) and **(C)** OD_600_ of TOP10 cells transformed with a *gfp.repL^WT^* (black line with black diamonds)*, gfp.repL^AT1^* (red line with black circles)*, gfp.repL^D1^* (green colored line with black rectangles)*, gfp.repL^AT2^* (orange line with black triangles), or *gfp.repL^D2^ gfp* (blue line with black triangles) reporter plasmid, at 1 to 10 hpi with 1.33 × 10^−4^ M of L-arabinose. All GFP fluorescence intensity were normalized to that of TOP10 cells transformed with an empty plasmid without *gfp* and *repL* genes. Induction assays were performed with 3 biological replicates and 4 technical replicates. Data were presented as mean ± SEM.

Arabinose induction of *repL* gene increased the output signal of a *gfp* reporter plasmid thus indicating a *cis*-acting, RepL-induced plasmid DNA replication in *E. coli* TOP10. We next sought to determine if the DnaA binding sites and A/T-rich regions are important for the *cis*-acting, RepL-induced signal amplification. Synonymous base substitutions (mostly A/T to G/C) were introduced in the A/T-rich regions (termed AT1 and AT2 hereafter) and DnaA binding sites (termed D1 and D2 hereafter) without affecting the amino acid sequence of *repL* gene product (Figure 3A). To assess if the mutations affected RepL-mediated signal amplification, *E. coli* TOP10 was transformed with the *gfp* reporter plasmid having a wildtype (WT, allele termed *repL*^WT^) or the mutant *repL* alleles, termed *repL^AT1^*, *repL^AT2^*, *repL^D1^* and *repL^D2^*. TOP10 transformants were induced with 1.33 × 10^−4^ M arabinose, and the GFP fluorescence intensity as well as OD_600_ of cells were recorded over a period of 10 h. The final output was represented as GFP fluorescence intensity per OD_600_ (GFP Fluo./OD_600_).

The *repL^AT1^* and *repL^D1^* alleles did not alter the increasing trend of GFP FIuo./OD_600_ values upon arabinose induction of TOP10 transformants, when compared to that of the *gfp*.*repL*^WT^ transformants (Figure 3B). Contrastingly, the *repL^AT2^* allele reduced the GFP Fluo./OD_600_ values of TOP10 transformants significantly by ~6.8-fold to ~10.7-fold between 2 to 10 hpi, when compared to the GFP Fluo./OD_600_ values of *gfp*.*repL*^WT^ transformants (*p* < 0.0005) (Figure 3B). Between 4 to 10 hpi, an average OD_600_ of ~0.5 was recorded for the *gfp*.*repL^AT1^*, *gfp*.*repL^D1^* and *gfp*.*repL*^WT^ transformants, which was ~2- to ~4-fold lower than the OD_600_ of *gfp*.*repL^AT2^* transformants (*p* < 0.0005) (Figure 3C). The *gfp*.*repL^D2^* transformants yielded intermediate OD_600_ values between ~0. 6 and ~0.9 at 4 to 10 hpi (Figure 3C). Although arabinose induction of *repL*^D2^ produced a similar increasing trend of GFP FIuo./OD_600_ values, the lower OD_600_ of *repL*^D2^ transformants might have provided a slightly higher GFP Fluo./OD_600_ values between 1 to 7 hpi, when compared to that of *repL*^WT^ transformants (Figures 3B,C).

To verify if the GFP signal amplification is directly associated with an increase in plasmid copy number and that mutations in the AT2 region of *repL* gene could reduce such RepL-mediated plasmid DNA replication, plasmid DNA were extracted from TOP10 *gfp.repL*^WT^ and *gfp.repL^AT2^* transformants at uninduced state, or at 8 h post induction with 1.33 × 10^−4^ M arabinose. Arabinose induction of the TOP10 *gfp.repL*^WT^ transformants increased the intensity of plasmid DNA band and the estimated plasmid copy number/OD_600_, which were consistent with the increase in GFP FIuo./OD_600_ values when compared to uninduced state (Supplementary Figures 4A,B). Contrastingly, arabinose induction of the TOP10 *gfp.repL ^AT2^* transformants did not increase the intensity of plasmid DNA band, nor the copy number of plasmid/OD_600_ significantly, when compared to that of uninduced state (Supplementary Figures 4A,B).

Taken together, synonymous base substitutions in the AT2 region significantly reduced RepL-mediated GFP signal amplification therefore indicating that the AT2 region might play an important role in RepL-mediated DNA replication.

### Mutations in the AT2 region of a *repL* gene did not affect the *trans*-acting, RepL-mediated signal amplification

**Figure 4:**
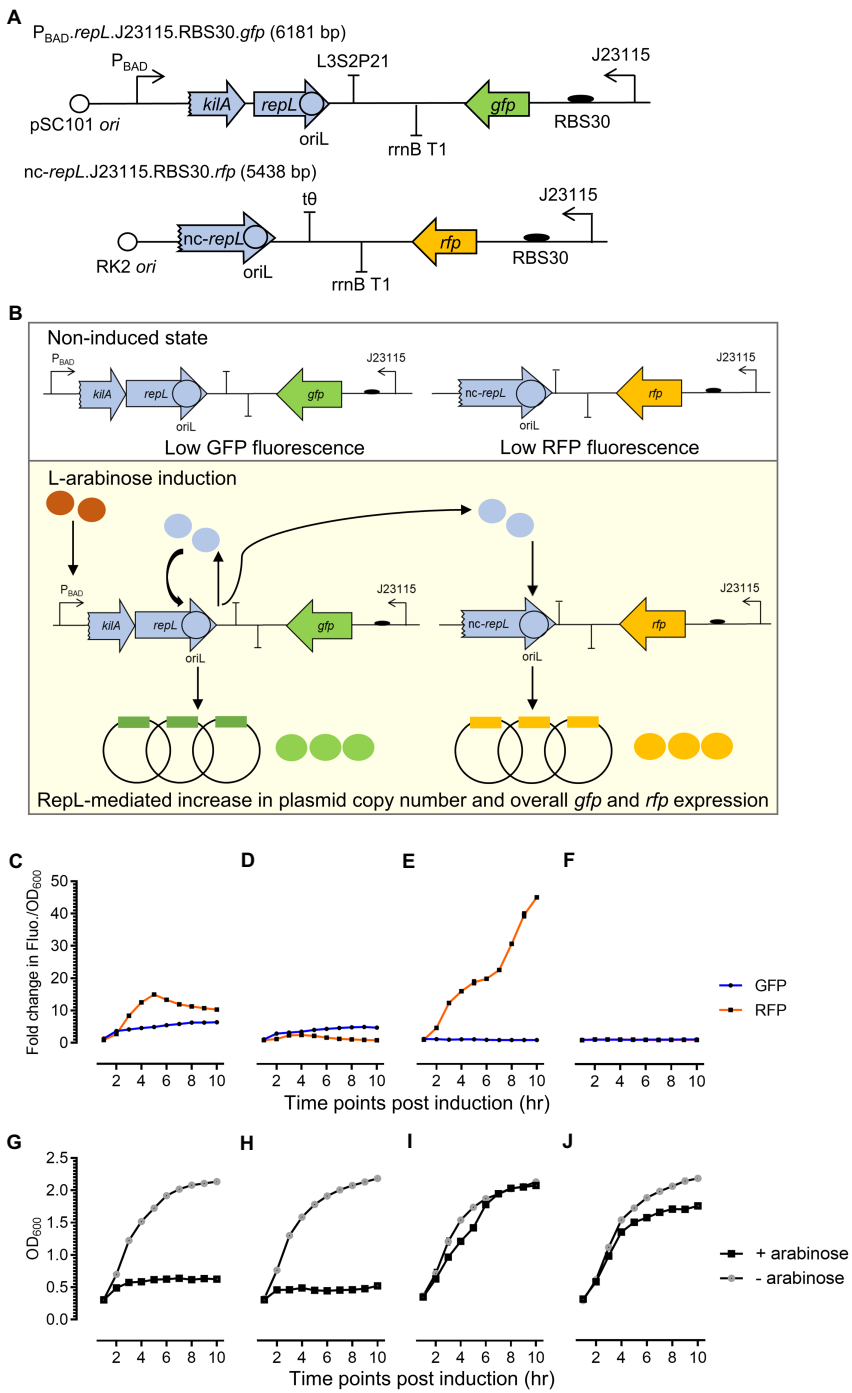
A *repL^AT2^* allele reduced the *cis*-acting RepL-mediated signal amplification while retaining the ability of its protein product to increase the output of a *rfp* plasmid *in trans*. **(A)** Schematic diagram of the *rfp* reporter construct. A *repL* gene sequence truncated by 40 bp at its 5′-end, was assembled in a *rfp*-encoding reporter plasmid. This non-protein coding *repL* gene, termed nc-*repL*, would only serve as a target site for RepL provided *in trans*. *Rfp* (in orange) was constitutively expressed under a weak constitutive promoter, Bba_J23115, with an artificial ribosomal binding site, RBS30. The *rfp.*nc-*repL* plasmid confers kanamycin resistance and has a RK2 oriV conferring low plasmid copy number. **(B)** Schematic diagram of RepL-mediated DNA replication *in cis* and *in trans*. Upon L-arabinose induction of *repL* gene expression, the protein product would target the oriL within its own gene sequence (*in cis*) as well as one within the nc-*repL* sequence (*in trans*). This would lead to an increase in the copy number of both reporter plasmids and therefore, giving an increase in GFP and RFP output compared to uninduced state. Fold changes in GFP (in blue) and RFP (in orange) fluorescence intensity per OD_600_ (Fluo./OD_600_) values of TOP10 transformed with **(C)** *gfp.repL*^WT^ and *rfp*.nc-*repL*^WT^ plasmids, **(D)** *gfp.repL*^WT^. and *rfp*.nc-*repL^AT2^* reporter plasmids, **(E)** *gfp.repL^AT2^* and *rfp.*nc-*repL*^WT^ reporter plasmids, or **(F)** *gfp.repL^AT2^* and *rfp.*nc-*repL^AT2^* reporter plasmids at 1 to 10 hpi with 1.33 ×10^−4^ M of L-arabinose. The OD_600_ of arabinose-induced TOP10 cells (+ arabinose, in black) and uninduced TOP10 cells (− arabinose, in gray) with **(G)** *gfp.repL*^WT^ and *rfp*.nc-*repL*^WT^ reporter plasmids, **(H)** *gfp.repL*^WT^ and *rfp*.nc-*repL^AT2^* reporter plasmids, **(I)** *gfp.repL^AT2^* and *rfp*.nc-*repL*^WT^ reporter plasmids, and **(J)** *gfp.repL^AT2^* and *rfp*.nc-*repL^AT2^* reporter plasmids were measured at 1 to 10 hpi. Fold changes in GFP and RFP FIuo./OD_600_ were calculated based on comparison between arabinose-induced and uninduced state. All GFP and RFP fluorescence intensity were normalized to that of TOP10 cells transformed with empty plasmids without *gfp* and *repL* genes or without *rfp* gene and nc-*repL* sequence. All experiment was performed with 3 biological replicates and 4 technical replicates. Data were presented as mean ± SEM.

The *repL^AT2^* allele consists of synonymous base substitutions, which were not expected to affect RepL amino acid sequence and/or the protein product activity. If this assumption is correct, the protein product of a *repL^AT2^* allele could potentially promote DNA replication of a plasmid *in trans* while the *cis*-acting, plasmid amplification process would be inhibited. Therefore, to determine if arabinose induction of a *repL^AT2^* allele can amplify the copy number of a plasmid *in trans*, the level of RepL-induced DNA replication was assessed semi-quantitatively using a *gfp* and *rfp* double reporter system (Figures 4A,B). A truncated *repL* gene sequence (termed nc-*repL* hereafter) derived from a P1 phagemid established previously (Kittleson et al., 2012; Huan et al., 2022), was assembled in a *rfp* reporter plasmid. The nc-*repL* sequence lacks a start codon and a preceding promoter sequence, which will only serve as a target site for RepL protein provided in *trans* (Figure 4B). The *rfp*.nc-*repL* and the *gfp.repL* plasmids with or without mutations in the AT2 region(s) (of *repL* gene and/or nc-*repL* sequence) were used for transformation of *E. coli* TOP10, followed by arabinose induction of the transformants. The GFP and RFP fluorescence intensity per OD_600_ (Fluo./OD_600_) were compared to that of uninduced state.

Arabinose induction of the *gfp.repL*^WT^ and *rfp.*nc-*repL*^WT^ transformants yielded a maximum ~5.2-fold (*p* < 0.0005) and ~ 10.5-fold (*p* < 0.0005) increase in GFP and RFP Fluo./OD_600_ respectively, when compared to uninduced state (Figure 4C). The OD_600_ of *gfp.repL*^WT^ and *rfp.*nc-*repL*^WT^ transformants were ~ 0.5 between 6 to 10 hpi, which was ~4-fold lower than the OD_600_ of uninduced cells (*p* < 0.0005) (Figure 4G). Arabinose induction of the *gfp.repL*^WT^ and *rfp.*nc-*repL^AT2^* transformants yielded a ~ 7.4-fold lower RFP Fluo./OD_600_ values (*p* < 0.0005), with no significant difference in the GFP Fluo./OD_600_ values (*p* > 0.05) between 4 to 6 hpi, when compared to that of the *gfp.repL*^WT^ and *rfp.*nc-*repL*^WT^ transformants (Figure 4D). An average O_600_ of ~0.5 was recorded for the *gfp.repL*^WT^ and *rfp*.nc-*repL^AT2^* transformants between 6 to 10 hpi, which was ~4-fold lower than the O_600_ of uninduced cells (*p* < 0.0005) (Figure 4H).

Contrastingly, arabinose induction of the *gfp.repL^AT2^* and *rfp*.nc-*repL*^WT^ transformants yielded a ~ 4.7-fold lower GFP Fluo./OD_600_ values (*p* < 0.0005) while the RFP Fluo./OD_600_ was increased by ~4-fold (*p* < 0.0005) at 10 hpi, when compared to that of the *gfp.repL*^WT^ and *rfp.*nc-*repL*^WT^ transformants (Figure 4E). Unexpectedly, an increase in the OD_600_ of *gfp.repL^AT2^* and *rfp*.nc-*repL*^WT^ transformants was observed between 2 to 6 hpi, with no significant differences in its OD_600_ values when compared to uninduced state between 6 to 10 hpi (*p* > 0.05) (Figure 4I). There was no significant increase in both GFP and RFP FIuo./OD_600_ values of the *gfp.repL^AT2^* and *rfp*.nc-*repL^AT2^* transformants, when compared to uninduced state at 10 hpi (*p* > 0.05) (Figure 4F). The OD_600_ of *gfp.repL^AT2^* and *rfp*.nc-*repL^AT2^* transformants showed an increasing trend between 2 to 10 hpi, yet the average OD_600_ was ~1.3-fold lower when compared to that of uninduced cells between 6 to 10 hpi (*p* < 0.05) (Figure 4J).

Taken together, arabinose induction of a *repL*^AT2^ allele increased the output signal of a *rfp* reporter plasmid *in trans* while the *cis*-acting *gfp* plasmid amplification was inhibited therefore indicating that the mutations in AT2 region of a *repL* gene might not have affected the protein product activity.

### RepL-mediated signal amplification in other laboratory strains of *Escherichia coli*

To determine if the *cis*- and *trans*-acting RepL-mediated signal amplification could be reproduced in other laboratory strains of *E. coli*, arabinose induction of the *gfp.repL*^WT^ and *rfp*.nc-*repL*^WT^ reporter constructs was repeated on *E. coli* NCM3722 and *E. coli* BL21. The bacterial cells were transformed with the plasmids, followed by arabinose induction of *repL* gene expression. The GFP and RFP fluorescence intensity per OD_600_ (Fluo./OD_600_) were compared to that of uninduced state.

An increase in both GFP and RFP FIuo./OD_600_ values were observed in NCM3722 and BL21 transformants, between 2 to 10 hpi when compared to that of uninduced cells (Supplementary Figures 5A,B). The NCM3722 transformants yielded a ~ 4.3-fold (*p* < 0.0005) and a ~ 16.6-fold (*p* < 0.0005) increase in GFP and RFP FIuo./OD_600_ values respectively, between 3 and 4 hpi when compared to uninduced state (Supplementary Figure 5A). BL21 transformants, however, produced a maximum ~4.7-fold (*p* < 0.0005) and ~ 26.5-fold (*p* < 0.0005) increase in GFP and RFP FIuo./OD_600_ values respectively, between 2 and 6 hpi when compared to uninduced state (Supplementary Figure 5B). Between 1 and 10 hpi, the OD6_00_ of NCM3722 transformant increased from ~0.5 to ~2.0 (Supplementary Figure 5C). Contrastingly, the OD6_00_ of BL21 transformants increased gradually from ~0.3 to ~0.5 between 2 to 5 hpi, followed by a sharp increase from approximately ~0.5 to ~2.1 between 6 to 10 hpi (Supplementary Figure 5D).

Overall, arabinose induction of *repL* gene in *E. coli* BL21 and *E. coli* NCM3722 increased the output of both *rfp* and *gfp* reporter plasmids, which was indicative of *cis*- and *trans*-acting RepL-mediated DNA replication in two laboratory strains of *E. coli*.

### Truncated nc-*repL* sequences allowed RepL-mediated signal amplification *in trans*

**Figure 5:**
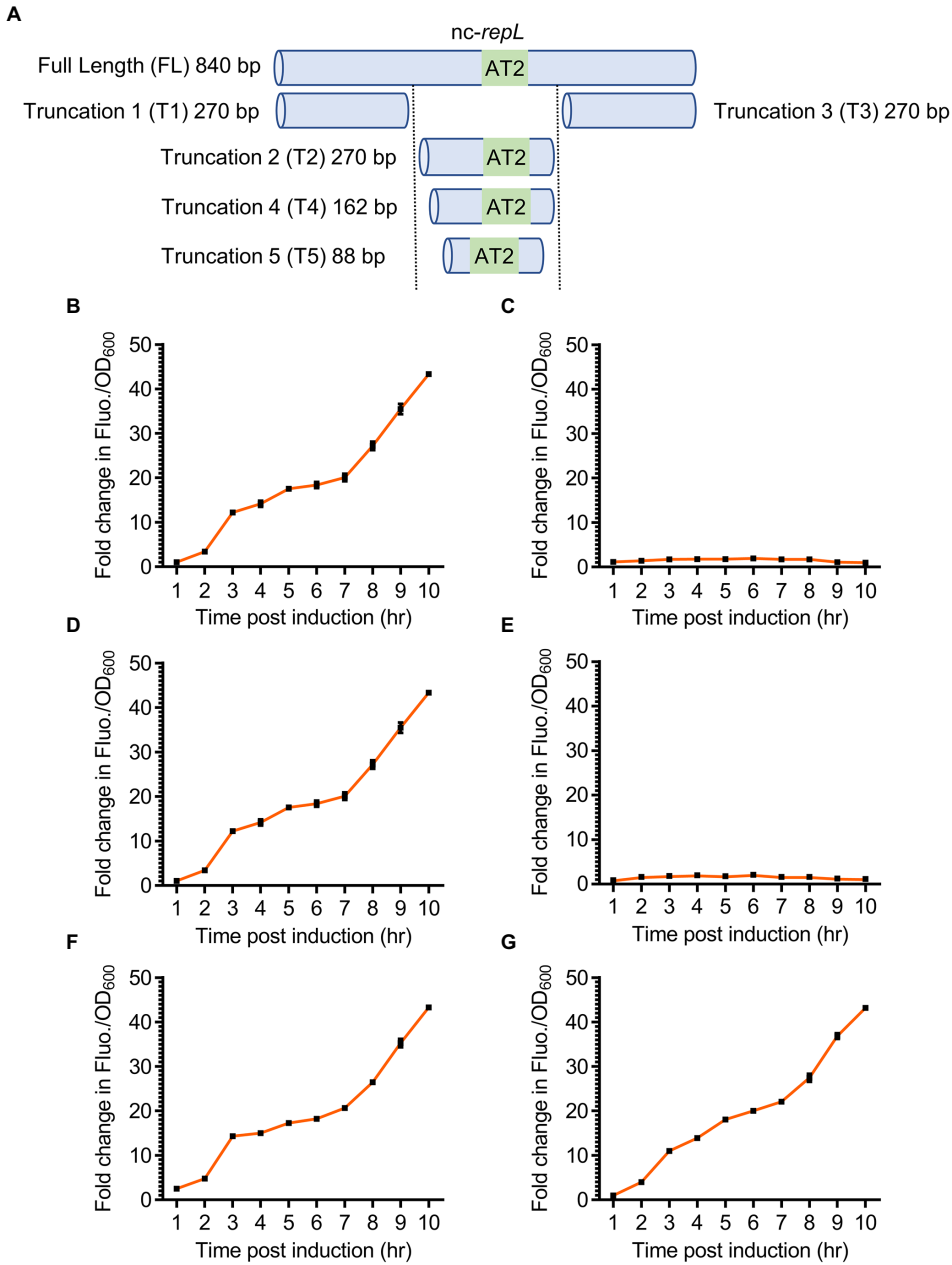
Truncated nc-*repL* sequences could serve as a target site for RepL-mediated signal amplification *in trans*. **(A)** Schematic diagram showing a full length and truncated versions of a non-coding *repL* (nc-*repL*) sequence, which were assembled onto a *rfp* reporter plasmid. Truncation 1 (T1) and Truncation 3 (T3) nc-*repL* do not contain the AT2 region (43 bp). The sizes of truncated nc-*repL* sequence were shown. Quantification of the fold change in RFP fluorescence intensity per OD_600_ (FIuo./OD_600_) of TOP10 cells co-transformed with a *gfp*.*repL^AT2^* plasmid and a *rfp* plasmid with **(B)** full length, **(C)** truncation 1 (T1), **(D)** truncation 2 (T2), **(E)** truncation 3 (T3), **(F)** truncation 4 (T4), or **(G)** truncation 5 (T5) versions of a nc-*repL* sequence at 1 to 10 hpi with 1.33 × 10^−4^ M of L-arabinose (orange). Fold changes in the RFP FIuo./OD_600_ were calculated based on comparison between arabinose-induced and uninduced state. All RFP fluorescence intensity values were normalized to that of TOP10 cells transformed with empty plasmids without *rfp* gene and nc-*repL* sequence. Induction assays were performed with 3 biological replicates and 4 technical replicates. Data were presented as mean ± SEM.

We next sought to determine if a shorter *repL* sequence could support a *trans*-acting RepL-mediated DNA replication, as the data would narrow down the location and sequence of P1 oriL. Three truncated versions of the nc-*repL* sequence, (termed T1 to T3 nc-*repL*) were designed and assembled into the *rfp* reporter plasmid (Figure 5A). *E. coli* TOP10 was co-transformed with the *gfp.repL^AT2^* plasmid (refer to Figure 4E), which would provide RepL expression *in trans* without amplification of *repL* gene copy number, as well as a *rfp* reporter plasmid having full length or truncated nc-*repL* sequence. Transformed cells were induced with 1.33 × 10^−4^ M arabinose and assessed for changes in the RFP fluorescence intensity per OD_600_ (RFP FIuo./OD_600_) when compared to uninduced states.

T2 nc-*repL* construct produced a maximum ~43.3-fold increase in RFP Fluo./OD_600_ value at 10 hpi, which was not significantly different when compared to that recorded with a full length nc-*repL* sequence (*p* > 0.05) (Figures 5B,D). On the contrary, both T1 and T3 nc-*repL* constructs yielded a ~ 22.4-fold lower RFP Fluo./OD_600_ values at 10 hpi, when compared to that of full length and T2 nc-*repL* constructs (*p* < 0.0005) (Figures 5B–E). Taken together, the T2 nc-*repL* sequence should contain all necessary elements to support a *trans*-acting RepL-mediated DNA replication process. Previous data indicated that the AT2 region located at the 3′-end of T2 nc-*repL* sequence might be important for RepL-mediated DNA replication process (Figure 3B). Contrastingly, synonymous base substitution in the preceding AT1 region and DnaA binding sites did not affect the *cis*-acting, RepL-mediated GFP signal amplification in *E. coli* TOP10 (Figure 3B). We therefore hypothesized the AT1 region and DnaA binding site might not be required for a *trans*-acting RepL-induced DNA replication and the T2 nc-*repL* sequence could be further truncated from its 5′-end. T2 nc-*repL* was further truncated into T4 nc-*repL* which contains AT1, AT2 regions and DnaA binding site 1, as well as a T5 nc-*repL* sequence which only contains the AT2 region (Figure 5A). Both T4 and T5 nc-*repL* sequences were assembled in the *rfp* plasmid, and the arabinose induction assay was repeated on TOP10 *gfp.repL^AT2^* and the *rfp* plasmids co-transformants. Both T4 and T5 nc-*repL* constructs produced a maximum ~43.2-fold increase in RFP FIuo./OD_600_ value at 10 hpi when compared to uninduced state, which was not significantly different from that recorded with full length nc-*repL* or the T2 nc-*repL* construct (*p* > 0.05) (Figures 5F,G). An OD_600_ range of ~1.6 to ~2.0 was recorded for TOP10 transformants with the T2, T4 and T5 nc-*repL* constructs between between 6 to 10 hpi, which was similar to that of TOP10 transformants with a full length nc-*repL* construct (*p* > 0.05) (Supplementary Figure 6).

Taken together, truncated nc-*repL* sequences with an intact AT2 region allowed the RepL-mediated RFP signal amplification, which suggested that these sequences could potentially serve as a target site for *trans*-acting RepL protein.

### Single or combination of mutations in the AT2 region of a nc-*repL* sequence provided varying levels of RepL-mediated signal amplification

**Figure 6:**
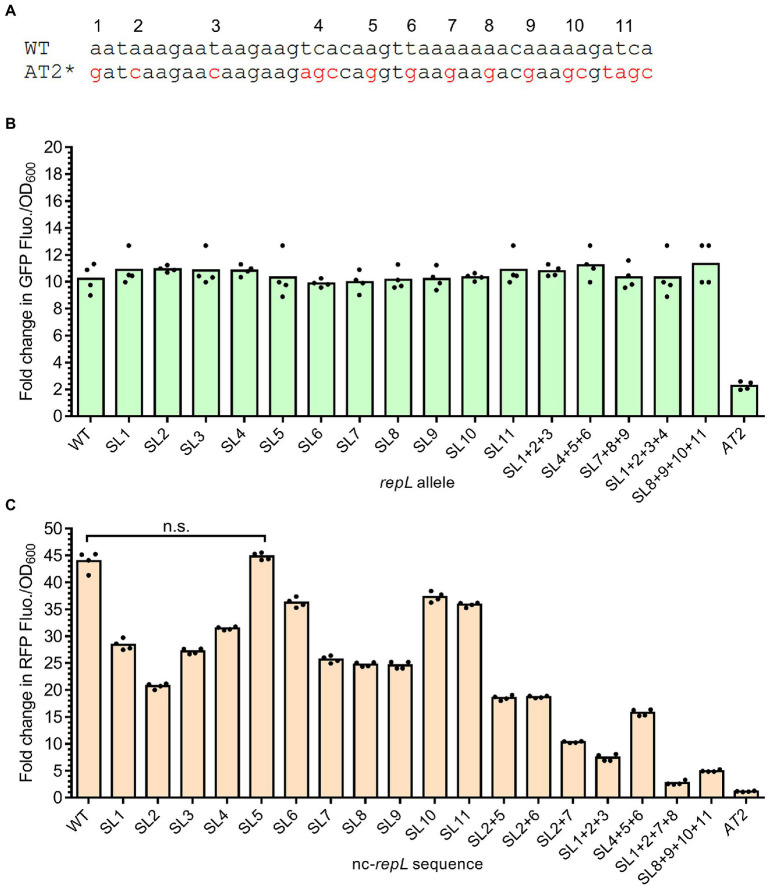
Base substitution at single or combination of positions within the AT2 region of a nc-*repL* sequence produced varying levels of RepL-mediated RFP signal amplification. **(A)** Schematic diagram showing the differences in DNA bases between wildtype (WT) and *repL^AT2^* (AT2*) sequences. Characters in red represent DNA base(s) which were substituted, and the mutations were numbered from 1 to 11 based on their position within the AT2 region. Base substitution at a single position would be named as “SL (position of nucleotide substitution),” while combinations of mutations would be named as “SL (position of nucleotide substitution) + (position of nucleotide substitution).” A *repL^AT2^* allele or a nc-*repL^AT2^* sequence contain base substitutions at all of the 11 positions. **(B)** Fold change in GFP fluorescence intensity per OD_600_ of TOP10 cells (GFP FIuo./OD_600_) transformed with a SL *gfp.repL*, *gfp.repL^AT2^* (*AT2*) or *gfp.repL*^WT^ (WT) plasmid at 10 hpi with 1.33 × 10^−4^ M of L-arabinose. Fold changes in the GFP FIuo./OD_600_ were calculated based on comparison between arabinose-induced and uninduced state. All GFP fluorescence intensity values were normalized to that of TOP10 cells transformed with an empty plasmid without *gfp* and *repL* genes. Comparisons of the fold changes in GFP Fluo./OD_600_ values between WT and all SL *repL* alleles with Welch’s ANOVA yielded a value of p of 0.7654, indicating no significant differences in the GFP output level between different samples. **(C)** Quantification of the fold change in RFP fluorescence intensity per OD_600_ of TOP10 cells (RFP FIuo./OD_600_) co-transformed with a *gfp.repL^AT2^* plasmid and a SL *rfp.*nc*-repL*, *rfp.*nc*-repL^AT2^* (*AT2*) or *rfp.*nc-*repL*^WT^ (WT) plasmid at 10 hpi with 1.33 × 10^−4^ M of L-arabinose. Fold changes in RFP Fluo./OD_600_ were calculated based on comparison between arabinose-induced and uninduced state. Comparisons in the fold changes in RFP Fluo./OD_600_ values between WT and all SL nc-*repL* constructs with Welch’s ANOVA yielded a value of p of 0.028, indicating significant differences in the RFP output level between different samples. n.s. represented no significant difference(s) in the RFP Fluo./OD_600_ values between WT and SL5 nc-*repL* constructs (*p* > 0.05). All RFP fluorescence intensity values were normalized to that of TOP10 cells transformed with empty plasmids without *rfp* gene and nc-*repL* sequence. The induction assay was performed with 4 biological repeats, with each data point represented the average value of 3 biological replicates. Data were presented as mean ± SEM.

The *repL^AT2^* mutation consists of DNA base substitutions at 11 positions which altogether inhibited the RepL-mediated signal amplification responses. We therefore hypothesized that mutation(s) at single or combination of positions within AT2 might produce varying levels of RepL-mediated signal amplification, which could be useful for biosensor-based application. To verify this hypothesis, mutation at each of the 11 positions within the AT2 region were introduced in a *repL* gene, followed by assembly of these *repL* alleles, termed SL *repL* alleles hereafter, in a *gfp* plasmid (Figure 6A). TOP10 cells were transformed with a *gfp* reporter plasmid having wildtype, *repL^AT2^* or one of the SL *repL* alleles. Transformed cells were induced with 1.33 × 10^−4^ M arabinose and assessed for changes in the GFP fluorescence intensity per OD_600_ (GFP FIuo./OD_600_) at 10 hpi when compared to uninduced states. Unexpectedly, there were no significant differences in the GFP Fluo./OD_600_ values between wildtype *repL* and all the SL *repL* transformants (*p* > 0.05) (Figure 6B). To determine if combinations of mutations at 3 or 4 positions within AT2 region would affect the RepL-mediated signal amplification, the corresponding SL *repL* alleles were assembled in the *gfp* reporter plasmid and arabinose induction assay was repeated on *E. coli* TOP10 plasmid transformants. Similarly, all of the SL *repL* alleles produced a ~ 10-fold increase in GFP Fluo./OD_600_ when compared to uninduced state, which was not significantly different to that observed after induction of wildtype *repL* allele (*p* > 0.05) (Figure 6B).

Upon arabinose induction of *repL* gene expression, gene transcription and/or a higher level of RepL protein due to amplification of its gene copy number could potentially overcome the modulatory effect(s) of SL mutation(s), if any, on the *cis*-acting, RepL-mediated DNA replication. If this is true, single or combinations of mutations in the AT2 region of a non-protein coding *repL* sequence (nc-*repL*) could potentially produce varying levels of *trans-*acting RepL-mediated DNA replication. Therefore, base substitution(s) at each of the 11 positions within AT2 region were introduced in a nc-*repL* sequence and assembled in the *rfp* reporter plasmid. *E. coli* TOP10 was co-transformed with a *gfp.repL^AT2^* plasmid, which would provide RepL expression *in trans* without amplification of its gene copy number (refer to Figure 3E), as well as a *rfp* plasmid having wildtype, nc-*repL^AT2^* or one of the SL nc-*repL* sequences. Transformed cells were induced with 1.33 × 10^−4^ M arabinose and assessed for changes in the RFP fluorescence intensity per OD_600_ (RFP FIuo./OD_600_) at 10 hpi when compared to uninduced states. All of the SL nc-*repL* transformants, except for SL5 nc-*repL*, yielded a range of ~16.2- to ~40.4-fold increase in RFP Flou./OD_600_ at 10 hpi when compared to uninduced states, which were intermediary between that of WT nc-*repL* and nc-*repL^AT2^* transformants (Figure 6C). Contrastingly, SL5 nc-*repL* transformants yielded a ~ 45.7-fold increase in RFP Fluo./OD_600_ at 10 hpi, which was not significantly different when compared to that of WT nc-*repL* transformants (*p* > 0.05) (Figure 6C). We next sought to determine if combinations of base substitutions within AT2 region of a nc-*repL* sequence could give a greater range of RFP output. Hence, we have introduced base substitutions at several positions within AT2 region of a nc-*repL* sequence and assembled the SL nc-*repL* sequences in the *rfp* reporter plasmid. Arabinose induction assay was repeated on TOP10 cells co-transformed with a *gfp.repL^AT2^.* plasmid and a *rfp* plasmid having wildtype, nc-*repL^AT2^* or one of the SL nc-*repL* sequences. Combination of mutations within AT2 region of a nc-*repL* sequence produced a range of ~1.2- to ~19.0- fold increase in RFP Fluo./OD_600_ values at 10 hpi, which were intermediary between that of nc-*repL^AT2^* and the SL nc-*repL* constructs having base substitution(s) at a single position (Figure 6C).

Taken together, base substitution at a single or multiple positions within the AT2 region of a nc-*repL* sequence produced varying levels of RepL-mediated RFP signal amplification, which indicated that the mutations could potentially cause varying levels of *trans*-acting, RepL-mediated DNA replication.

### SL nc-*repL* sequences could modulate the output of an arsenic biosensor

**Figure 7:**
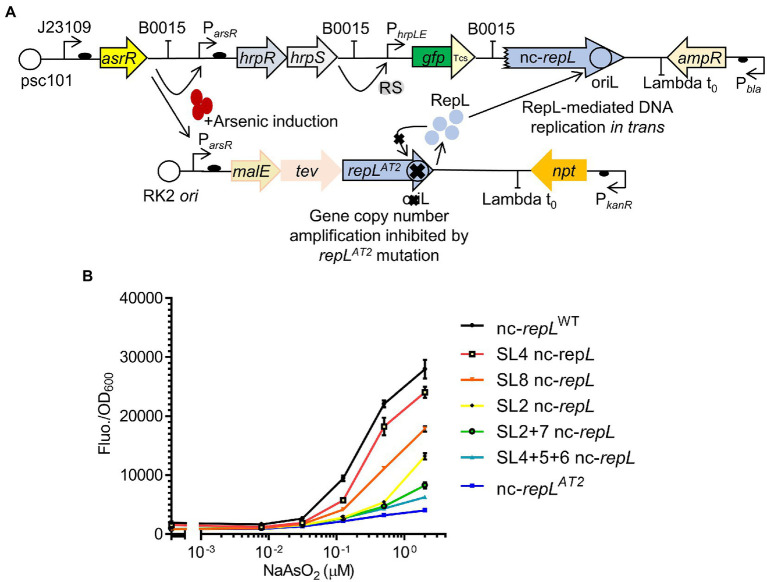
SL nc-*repL* sequences could modulate the output of an *E. coli*-based arsenic biosensor. **(A)** Schematic diagram showing the incorporation of *repL^AT2^* gene and T2 nc-*repL* sequence into an *arsR*-based arsenic biosensor. The biosensor circuit consists of two separate plasmids. Presence of sodium arsenite/arsenic induces the expression of *hrpR*, *hrpS*, as well as *malE*, *tev* and *repL^AT2^*. RepL protein would promote DNA replication of the *gfp* reporter plasmid containing a nc-*repL* sequence. This would give an increase in GFP fluorescence intensity. The mutant *repL^AT2^* allele would inhibit amplification of its gene copy number *in cis*. GFP has a Tev protease degradation tag (Tsc) which reduced the background protein level at uninduced state. **(B)** GFP fluorescence intensity per OD_600_ of TOP10 cells (Fluo./OD_600_) transformed with the arsenic biosensor having a wildtype (nc-*repL*^WT^, in black), nc-*repL^AT2^* (blue), SL4 nc-*repL* (red), SL8 nc-*repL* (orange), SL2 nc-*repL* (yellow), SL2 + 7 nc-*repL* (green), SL4 + 5 + 6 nc-*repL* (teal) sequences, at 5 hpi with 0.0781 μM, 0.031 μM, 0.125 μM, 0.5 μM to 2.0 μM of sodium arsenite (NaAsO_2_). Induction assays were performed with 3 biological replicates and 3 technical replicates. Data were presented as mean ± SEM.

Single or combination of base substitutions within the AT2 region of a nc-*repL* sequence produced varying levels of RepL-mediated RFP signal amplification, therefore indicating that the SL nc-*repL* sequences could potentially modulate the output of other genetic circuits. As a proof of concept, we have integrated the *repL*^AT2^ allele and nc-*repL* sequences into a GFP-expressing, *E. coli*-based arsenic biosensor developed by Wan et al. (2019) (Figure 7A). The modified biosensor would provide an arsenic-inducible *repL^AT2^* expression, which will produce RepL protein to promote DNA replication *in trans* without amplification of the *repL^AT2^* gene copy number (Figure 7A). *E. coli* TOP10 was transformed with the plasmids and the GFP fluorescence intensity as well as OD_600_ were measured at 5 hpi with a range of sodium arsenite (NaASO_2_). The final output was represented as GFP fluorescence intensity per OD_600_ (GFP Fluo./OD_600_).

A maximum ~17.8-fold increase in GFP Fluo./OD_600_ value was observed for TOP10 nc-*repL*^WT^ transformants at 5 hpi with 2 μM NaASO_2_, when compared to that at uninduced state (*p* < 0.0005) (Figure 7B). We next sought to determine if the SL nc-*repL* and nc-*repL^AT2^* sequences could modify the RepL-mediated signal amplification. We have chosen SL2, SL8, SL2 + 7 or SL4 + 5 + 6 nc-*repL* to replace the nc-*repL*^WT^ sequence since these constructs produced distinct, intermediary levels of RepL-mediated signal amplification when compared to that of a nc-*repL*^WT^ and a nc-*repL^AT2^* constructs (Figure 6C). At 2 μM NaASO_2_, the nc-*repL^AT2^* construct significantly reduced the GFP FIuo/OD_600_ value by ~6.9-fold, when compared to that of a nc-*repL*^WT^ construct (*p* < 0.0005) (Figure 7B). Contrastingly, SL4, SL8, SL2, SL2 + 7 and SL4 + 5 + 6 nc-*repL* constructs produced a ~ 1.2-fold (*p* > 0.05), ~1.6-fold (*p* < 0.05), ~2.2-fold (*p* < 0.005), 3.4-fold (*p* < 0.0005) and ~ 4.5-fold (*p* < 0.0005) lower GFP Fluo./OD_600_ respectively, when compared to that of a nc-*repL*^WT^ construct at 2 μM NaASO_2_ (Figure 7B).

Taken together, a combination of *repL^AT2^* allele and nc-*repL*^WT^ sequence amplified the GFP output of an arsenic biosensor while mutations within the AT2 region of nc-*repL* sequence produce varying levels of GFP signal amplification, therefore demonstrating the potential use of *repL* constructs to modulate the output of biosensors.

## Discussion

RepL expression is critical for the lytic replication of bacteriophage P1, in which the protein was thought to initiate DNA replication from the oriL, postulated to be located at the second half of its gene sequence. While the molecular mechanisms behind RepL activity remained unknown, the protein activity could induce plasmid DNA replication in *E. coli*, which would allow investigations into the identity P1 oriL sequence, as well as candidate host factor(s) required for the RepL-mediated process. We have identified an A/T-rich region, termed AT2, whereby synonymous mutations within this sequence significantly affected the RepL-mediated signal amplification therefore suggesting an important role of this previously unidentified element in RepL-mediated DNA replication. Furthermore, a truncated *repL* sequence of 88 bp in size allowed RepL-mediated signal amplification *in trans* thus indicating a minimal sequence that could serve as RepL target site and contain all the DNA elements required for RepL-mediated plasmid DNA replication. Consistent with previous studies by Sheth et al. (2017), Dwidar and Yokobayashi (2019), and Li et al. (2023), we have demonstrated that the RepL could amplify the signal of an arsenic biosensor. Furthermore, our data indicated that base substitution(s) at a single or several positions within the AT2 region produced varying levels of RepL-mediated signal amplification. Taken together, our study provides novel insights into *cis*-acting DNA element(s) required for RepL-mediated DNA replication in *E. coli*, as well as demonstrating that *repL* alleles and non-(protein) coding *repL* sequences could be integrated into genetic circuits to achieve varying levels of signal amplification.

### The AT2 region might be integral to P1 lytic replicon

A/T-rich regions often serve as DNA Unwinding Elements (DUE) of an origin of replication, by facilitating the opening of a DNA double helical structure (Coman and Russu, 2005; Rajewska et al., 2012). The positioning and sequence of A/T-rich repeats are critical for the function of DUE, whereby point mutation and single base insertion could inactivate the origin (Saha et al., 2004; Kowalczyk et al., 2005). Similarly, synonymous base substitution at 11 positions within the AT2 region of a *repL* sequence significantly impaired the RepL-mediated signal amplification response, which indicated that the AT2 region could potentially be a DUE of P1 oriL. Contrastingly, base substitutions in the AT1 region which overlaps with a previously identified IHF binding site, as well as the two DnaA binding site did not affect RepL-mediated signal amplification significantly. Furthermore, a truncated nc-*repL* sequence without the IHF and DnaA binding sites allowed RepL-mediated signal amplification *in trans*. Therefore, IHF and DnaA might not be required for RepL-mediated plasmid DNA replication *in trans*. Interestingly, the AT2 region is preceded by a cytosine/guanine rich in sequence yet the role of such steep changes in DNA composition on RepL-mediated DNA replication was not determined in this study. We could not, however, determine if RepL would bind directly to the AT2 region and/or to the preceding sequence, as well as functional redundancy between RepL and other host proteins involved in DNA replication, if any. Hence, structural study of RepL protein, as well as the use of a AT2-containing DNA sequence and purified RepL protein, could provide insights into the role of AT2 region in RepL-mediated DNA replication. Besides molecular studies of RepL activity, the ability to purify RepL protein would be useful to verify if the synonymous mutations, if any, in *repL* gene would affect the protein activity, despite the fact that arabinose induction of a *repL*^AT2^ allele could amplify the signal of another plasmid *in trans*. Our preliminary experiments indicated that the addition of a PolyHistidine Tag-encoding sequence in the C-terminus of *repL*^WT^ and *repL^AT2^* genes (6X PolyHis) did not affect the RepL-mediated signal amplification *in cis* and *in trans* (Supplementary Figure 7). We have, however, experienced several issues during purification of the His-tagged RepL protein, such as the presence of multiple protein contaminants after elution (data not shown), which prompted optimisation of the RepL purification protocol in future studies. While the RepL-mediated responses were assessed on reporter plasmids, it is noteworthy that the effect(s) of mutations in *repL* gene sequence was not studied in the context of P1 genomic DNA, whereby variations in *repL* gene expression under its native promoter, as well as discrepancies in nucleotide composition and modifications (if any) when compared to plasmid DNA, might require other host and/or P1-derived factors for supporting RepL-mediated P1 DNA replication. Hence, the role of IHF and DnaA binding sites, as well as the AT2 region in the lytic stage replication of P1 genomic DNA remained to be elucidated. Furthermore, other nucleoproteins, such as H-NS and Fis which are associated with A/T-rich in regions, might play important role(s) in RepL mediated DNA replication process. Taken together, the truncated versions of nc-*repL* constructs of this study would provide an excellent platform to probe DNA binding of host factor(s) which are necessary for RepL-mediated DNA replication process, as well as a identifying the molecular mechanisms of RepL activity.

### High level of RepL activity might be cytotoxic to *Escherichia coli* TOP10

Although the mechanism of both *cis*- and *trans*-acting RepL-mediated DNA replication was expected to be similar, a *cis*-acting RepL-mediated signal amplification reduced the OD_600_ of *E. coli* TOP10 significantly when compared to uninduced state. The cytotoxicity associated with RepL activity was previously reported by Dwidar and Yokobayashi (2019), yet it was not observed in TOP10 *gfp* plasmid transformants without a *repL* gene, nor when the *cis*-acting signal amplification was inhibited by a *repL^AT2^* allele in this study. Furthermore, our results indicated an inverse relationship between the OD_600_ of TOP10 *gfp.repL*^WT^ transformants and arabinose concentration. Therefore, a high level of *cis*-acting RepL activity, caused by an amplification of its gene dosage, might be cytotoxic to *E. coli* TOP10 cells. Unexpectedly, the presence of a *cis*-acting, RepL-mediated signal amplification did not reduce the OD_600_ of *E. coli* NCM3722 significantly when compared to that of *E. coli* TOP10. Therefore, other host-derived factor(s) could potentially alleviate the cellular burden associated with high level of RepL activity in NCM3722. One such candidate would be the RecA recombinase, in which the protein-coding gene is supposedly deleted in *E. coli* TOP10 (Anton and Raleigh, 2006). RepL protein activity might be causing DNA strand breakages and/or the stalling of replication forks directly, which could potentially activate the SOS-mediated responses and causing an arrest in cell division (Cox et al., 2000; Pennington and Rosenberg, 2007). Alternatively, a high level of *cis*-acting RepL activity might increase the frequency of co-directional or head-on collision between replisome and transcription unit at the *repL* gene sequence, indirectly causing DNA damage and/or disruption to DNA replication which could potentially arrest cell division (French, 1992; Mirkin and Mirkin, 2005; Dutta et al., 2011; Merrikh et al., 2012). If this is true, RecA activity could repair DNA damages (Robu et al., 2001; Lusetti and Cox, 2002) and/or potentially resolve other consequences of RepL activity, which would then alleviate the cytotoxicity caused by high level of RepL activity in *E. coli* NCM3722. Therefore, restoring the *recA* gene expression in TOP10 cells, followed by a direct measurement of RepL protein level and activity, as well as investigating the interactions between purified RepL protein and its DNA substrate, could provide insights into how a *cis*-acting RepL-mediated DNA replication is cytotoxic to *E. coli* TOP10.

### SL nc-*repL* sequence could modulate the output of a biosensor and the limitations of this study

While a nc-*repL* sequence with base substitutions within the AT2 region could produce varying levels of RepL-mediated signal amplification, the modulatory effect of these mutations was not observed on the *cis*-acting, RepL-mediated signal amplification. Transcription preceding the *E. coli* oriC sequence was shown to promote opening of DNA duplex by DnaA (Baker and Kornberg, 1988), as well as changing the topology of 13-mer region which might facilitate DNA melting (Asai et al., 1992). Furthermore, a direct interaction between *E. coli* RNA polymerase and bacteriophage λO replication initiator protein suggested coupling mechanisms between transcription and the initiation of phage DNA replication (Szambowska et al., 2011). Therefore, the presence of *repL* gene transcription and/or a higher level of RepL activity due to amplification of its gene copy number, might overcome the modulatory effects of SL *repL* alleles on the *cis*-acting RepL-mediated DNA replication. Interestingly, the modulatory effect of SL nc-*repL* sequences suggested that base substitution(s) at certain positions within the AT2 region produced a greater inhibitory effect against RepL-mediated DNA replication than others. We have only examined a handful of SL *repL* alleles thus the effects of other forms of mutations (i.e., insertion and deletion) as well as base substitutions at different positions within AT2 and its adjacent sequences remained to be elucidated. Furthermore, the RepL-mediated signal amplification should be repeated in different strains of *E. coli* and *in vitro* such as in cell free-based system, as fluctuations and discrepancies in the growth rate of different *E. coli* strains would affect the efficiency of biosensors and the effect of RepL-mediated signal amplification. Although the *gfp* and *rfp* reporter plasmids allowed seamless time-point assessment of RepL-mediated responses, changes in the plasmid copy number, as well as *repL* gene expression were not measured directly in this study. We have, however, attempted agarose gel electrophoresis of DNA extracted from TOP10 transformants after induction of *repL*^WT^ and/or *repL^AT2^* gene expression, in which the results indicated amplification of the plasmid copy number *in cis*, as well as the copy number of a 11.6 kbp nc-*repL*^WT^-encoding cosmid *in trans*, respectively (Supplementary Figures 4, 8). Nevertheless, a greater panel of mutations within AT2 region as well as substitution of DNA bases at adjacent regions, coupled with a direct quantification of the reporter gene expression level, *repL* gene expression as well as the copy number of targeted plasmid with varying lengths and nucleotide composition, would aid in building a comprehensive list of *repL* allele and/or nc-*repL* sequence to provide a precise level of signal amplification for future biosensor-based applications.

## Materials and methods

### Bacterial strains, plasmids, and media

All bacterial strains used in this study were listed in Supplementary Table 1 while plasmids were listed in Supplementary Table 2. Bacteria were routinely cultured in Luria–Bertani (LB) broth and agar, unless stated otherwise. LB broth was prepared with 10 g/l of NaCl (Fisher Scientific, 7647), 10 g/l of tryptone (VWR, 9000-71-9) and 5 g/l of yeast extract (Formedium, YEA03). For LB agar, 20 g/l of agar powder (Formedium, AGA03) was added to LB medium. Antibiotics used in this study include ampicillin (100 μg/mL) and kanamycin (50 μg/mL).

### Heat-shock transformation of *Escherichia coli*

Transformation of *E. coli* TOP10 and BL21 was carried out *via* the heat shock method. Chemically competent bacterial cells were prepared by selecting a single bacterial colony and inoculated in 3 mL of LB medium. Cells were cultured for 16 h, at 37°C, with shaking. Overnight culture was sub-cultured by 1/100 in fresh LB medium at 1/100 dilution factor without antibiotics, until it reached an OD_600_ of approximately 0.33. Bacterial culture was cooled on ice for 15 min, followed by centrifugation at 4000 X g for 5 min at 4°C. Supernatant was removed and the cell pellet was washed with 25 mL of 5 mM CaCl_2_ solution for every 50 mL of bacterial culture. Cells were spun down at 4,000 X g for 5 min. The washing step was repeated, and the final cell pellet was resuspended in 25 mL of 5 mM CaCl_2_ solution and left on ice for 30 min. Cells were centrifuged at 4,000 X g for 5 min, and cell pellet was resuspended in 2 mL of 5 mM CaCl_2_ solution with 15% glycerol, for every 50 mL of bacterial culture and left on ice for 2 h. Cells were aliquoted into 1.5 mL microcentrifuge tubes, and stored at −80°C. For heat-shock transformation, 50 μL of chemically competent *E. coli* TOP10 and BL21 cells was pre-mixed with 10 ng of DNA (for each plasmid). Cells were incubated at 43°C on a heat block for 50 s. Cells were immediately chilled on ice for 3 min, followed by addition of 450 μL of SOC medium. Transformed cells were recovered in SOC for 1 h, at 37°C, with shaking. Thirty μL to 50 μL of recovered cells were plated onto LB agar with the appropriate antibiotic(s) and incubated at 37°C for 16 h.

### Polymerase chain reactions

All PCR reactions were carried out using Q5® High-Fidelity 2X Master Mix (NEB, M0491) following manufacturer’s protocol, with 1 ng of DNA sample as template. A primer concentration of 0.5 μM was used for all PCR reactions. Denaturation of template was carried out for 30 s at 98°C. The annealing temperature of primers were determined using The NEB T_m_ Calculator and this step was carried out for 30 s. Extension step was set at 72°C, for 30 s per kbp of DNA sample. A final extension step at 72°C for 2 min was added. Thirty cycles of amplifications were used. Mutations were introduced into *repL* sequence *via* PCR, whereby primers were designed to contain the desirable mutation (Supplementary Table 3). IDT codon optimisation tool (IDT) were used to select synonymous base substitution in *repL* gene sequence, emphasizing on A/T to G/C base substitution, if applicable. The annealing temperature of primers determined with NEB T_m_ Calculator was reduced by 3°C, to ensure proper annealing of the primers with mismatch mutation(s) to the template DNA. DNA of *E. coli* P1 lysogen, EMG16, was used as template for molecular cloning of *repL* gene sequence *via* colony PCR with Q5® High-Fidelity 2X Master Mix. Colonies of the bacteria were diluted in 100 μL of water, and 1 μL of the bacterial suspension was used as template. A 5 min of heating step at 95°C was added prior to the thermocycling steps. The annealing temperature of primers were determined using The NEB T_m_ Calculator and this step was carried out for 30 s. Extension step was set at 72°C, for 1 min per kbp of DNA sample. A final extension step at 72°C for 3 min was added. Thirty cycles of amplifications were used.

### Assembly and preparation of plasmids

All plasmids of this study were assembled *via* Gibson assembly, using the NEBuilder® HiFi DNA Assembly Master Mix (NEB, E2621), following the manufacturer’s protocol. Primers were designed to have 20 bp to 30 bp overlapping regions between DNA fragments. A total of 80 ng of DNA was used for each reaction, with a vector: insert ratio of 1:2. All DNA fragments were prepared *via* PCR. Single-stranded DNA (ssDNA) was used to assemble the T5 nc-*repL* sequence in a *rfp* plasmid (refer to Figure 5A). Two primers were designed to have 28 bp overlapping regions between them and with the vector DNA (Supplementary Table 3). A total of 80 ng of DNA was used for each reaction, with a vector: insert (double-stranded DNA, dsDNA): insert (ssDNA) ratio of 1:2:5. Two micro liter of the reaction mix was added to 50 μL of chemically competent *E. coli* TOP10 cells for heat-shocked transformation. Fifty microliter of cells recovered after transformation was plated on LB agar with the appropriate antibiotic and incubated at 37°C for 16 h. Three colonies were picked and used for the preparation of overnight cultures. Extraction of plasmid and phagemid DNA were carried out using Qiaspin 250 Miniprep Kit (Qiagen, 27,106), following the manufacturer’s protocol. All plasmids were sequenced-verified *via* Sanger Sequencing carried out by Source Bioscience (Nottingham). Sequences of plasmids are listed in Supplementary Table 4.

### Electroporation of *Escherichia coli*

Transformation of *E. coli* NCM3722 was carried out *via* electroporation. For preparing electrocompetent cells, overnight culture was sub-cultured in fresh LB medium at 1/100 dilution factor without antibiotics, until it reached an OD_600_ of approximately 0.40. Bacterial culture was cooled on ice for 15 min, followed by centrifugation at 4,000 X g for 5 min at 4°C. Supernatant was removed and the cell pellet was washed with 25 mL of 10% glycerol solution for every 50 mL of bacterial culture. Cells were centrifuged at 3,000 X g for 5 min. The washing step was repeated twice, and the final cell pellet was resuspended in 250 μL of 10% glycerol solution for every 50 mL of bacterial culture. Cells were aliquoted into 1.5 mL microcentrifuge tubes and stored at −80°C. Forty micro liter of chemically competent cells were pre-mixed with 10 ng of DNA for *E. coli* NCM3722. Cells were transferred into a Gene Pulser/MicroPulser Electroporation Cuvettes, 0.1 cm gap (Biorad, 1,652,083), and electroporation was carried out using MicroPulser Electroporator (Biorad, 652,100), with the “Bacteria” setting. Immediately after electroporation, cells were transferred into pre-chilled, 800 μL of SOC. Transformed cells were recovered in SOC for 1 h, at 37°C, with shaking. Twenty μL to 30 μL of recovered cells were plated onto LB agar with the appropriate antibiotic(s) and incubated at 37°C for 16 h.

### Arabinose induction of *repL* expression, quantification of GFP, RFP fluorescence intensity, and OD_600_

The level of RepL-mediated DNA replication was assessed semi-quantitatively, by measuring changes in the output of reporter plasmid(s) upon arabinose induction of *repL* gene expression. Overnight cultures were prepared in LB with the appropriate antibiotic(s), using at least 3 X randomly picked colonies, at 37°C with shaking. Overnight cultures were diluted by 1/100, in 200 μL of fresh LB medium with the appropriate antibiotic(s), in a black 96 wells μClear microplate (GBO, 655096). The plate was incubated at at 37°C in CLARIOstar Plus BMG Microplate reader (BMG Labtech), with a dual orbital shaking mode setting of 700 rpm. Cells were cultured until an OD_600_ of ~0.2, whereby (varying concentrations or 1.33 × 10^−4^ M of) L-arabinose was added to induce the expression of *repL*. OD_600_ as well as the GFP and RFP fluorescence intensities were recorded at every hour, for a period of 10 h. OD_600_ values were recorded at discrete wavelength of 600 nm, with a pathlength correction for 200 μL reaction and 5.88 mm length, settling time of 0.5 s, 20 flashes and a measurement start time of 0 s. The excitation and emission wavelengths used for GFP were 485–12 nm and 520 nm respectively, while that for RFP were 584 nm and 620 nm, respectively. Fluorescence intensities were recorded from the bottom of each well, with a settling time of 0.2 s, measurement start time of 0 s and 10 flashes. Data were analyzed using Omega software (BMG Labtech). All data were first normalized to readings obtained from blank wells which contained only LB medium, followed by normalization of fluorescence intensity values to that of cells transformed with empty vector (without *repL* and *gfp* or *rfp* and nc-*repL*), unless stated otherwise. Fluorescence intensity was then divided by OD_600_ (Fluo./OD_600_).

### Arsenic induction of *repL* expression and quantification of GFP output

The *arsR*-based arsenic biosensor was designed by our lab previously (Wan et al., 2019). The arsenite induction assay was performed on *E. coli* TOP10 using a protocol established by Wan et al., 2019. Briefly, overnight cultures of transformed TOP10 cells were prepared in LB with the appropriate antibiotic(s), using 3 randomly picked colonies, at 37°C with shaking. Overnight cultures were diluted by 1/100, in 200 μL of fresh LB medium with the appropriate antibiotic(s), in a black 96 wells μClear microplate. Cells were induced with varying concentrations of sodium arsenite (NaAsO_2_, Sigma, 35,000), followed by incubation in a CLARIOstar Plus BMG Microplate reader (BMG Labtech) at 37°C, with a dual orbital shaking mode setting of 700 rpm. GFP fluorescence intensity and OD_600_ of cells were recorded at 5 h post induction, with the same settings described in quantification of GFP and RFP output. All data were first normalized to readings obtained from blank wells which contained only LB medium. GFP fluorescence intensity was then divided by OD_600_ of cells (Fluo./OD_600_).

### Statistical analysis

Data of this study was generated from at least 3 biological repeats (host cells tested) and 3 technical repeats, unless as stated otherwise. Calculations of this study was carried out using Microsoft excel (Microsoft, Redmond, WA, United States). Welch’s ANOVA or two-way ANOVA for comparisons involving two independent variables (i.e., data involving different concentration of arabinose and at multiple time points post induction of *repL* expression) was used to determine the *value of p*s, followed by a Dunn’s multiple comparison post-hoc test to adjust the respective value of ps, *via* Graphpad Prism 6. A *p*-value of <0.05 is considered statistically significant for this study. Graphpad Prism 6 was used to generate the graphs of this study.

## Data availability statement

The datasets presented in this study can be found in online repositories. The names of the repository/repositories and accession number(s) can be found in the article/Supplementary material.

## Author contributions

BW: conceived and supervised the study. BW, YH, and RB: designed the experiments. YH: performed the experiments and data analysis. All authors took part in the interpretation of results. YH and BW wrote the manuscript. All authors contributed to the article and approved the submitted version.

## Funding

This work was supported by the Bill and Melinda Gates Foundation under the Grand Challenges Explorations grant (OPP1139488), and UK Research and Innovation Future Leaders Fellowship [MR/S018875/1]. BW is supported by the Fundamental Research Funds for the Central Universities (226-2022-00178, 226-2022-00214), the Natural Science Foundation of China (32271475) and Kunpeng Action Programme Award of Zhejiang Province.

## Conflict of interest

The authors declare that the research was conducted in the absence of any commercial or financial relationships that could be construed as a potential conflict of interest.

## Publisher’s note

All claims expressed in this article are solely those of the authors and do not necessarily represent those of their affiliated organizations, or those of the publisher, the editors and the reviewers. Any product that may be evaluated in this article, or claim that may be made by its manufacturer, is not guaranteed or endorsed by the publisher.

## Supplementary material

The Supplementary material for this article can be found online at: https://www.frontiersin.org/articles/10.3389/fmicb.2023.1095671/full#supplementary-material
