# Supplementary material for: An adenine/thymidine-rich region is integral to RepL-mediated DNA replication
Source: Front Microbiol. 2023 Feb 9;14:1095671. doi: 10.3389/fmicb.2023.1095671 (PMC9948254; doi:10.3389/fmicb.2023.1095671)
Supplement: Supplementary file 1 [file Data_Sheet_1.pdf]

**Supplementary Information for**

# "An adenine/thymidine-rich region is integral to RepL-mediated DNA replication"

Yang W. Huan[2], Russell Brown[2] and Baojun Wang[1,2,3]*

[1]College of Chemical and Biological Engineering & ZJU-Hangzhou Global Scientific and Technological Innovation Center, Zhejiang University, Hangzhou 310058, China

[2]School of Biological Sciences, University of Edinburgh, Edinburgh EH9 3FF, United Kingdom

[3]Research Center for Biological Computation, Zhejiang Laboratory, Hangzhou 311100, China

*correspondence (baojun.wang@zju.edu.cn)

## Table of Contents

Supplementary Figures 1-8

Supplementary Tables 1-4

References

1 95
atgctggctaaagtcactttcctgagctgtataacgatgagcgattttactttttctggctatgaattggcctgctttgtaacacactccggtct

96 183
atcccgtagcgccgggcatatcctgtcgcaatgtgcaaatctcgcggcaacaaccagtgaatacttcattcacaagcctcaccgcctg

184 273
atcgcggcagaaactggttatagccaatcaaccgtcgttcgtgcattccgtgaagctgtaaacaaaggaattctgtctgtagagattgtt

274 362
atcggcgatcaccgtgaacgtcgcgctaacctgtaccggtttacaccatcctttttggccttcgcacaacaagccaaaaatgcgctgat

363 415 438 441 450 454
agaaagcaaattaaagatctcttcagcggcaaccaaggttaaagctgttctcgctaagacattggctttatttaattttttatccacacccc
IHF binding site
AT1 DnaA 1

455 495 540
catgtcaaaatgataccccctcccccctgtcaggatgacgtggcaataaagaataagaagtcacaagttaaaaaacaaaaagatca
AT2

541 624
gtttccggcggtgccggaacaaccagcctcaaaaaattgacttcatggatcgctaaggcaaaagcaaaggctgacaatctgcgg

625 633 709
ttatccaaaaaacgcactcaaaaacatgagttcaagcagaaagtagaggcggctgcgcggaaatatgcttacctgaagaacaagc
DnaA 2

710 799
gttcgcctgatattggcgggatatcaaacttcgataacctaccgcattgcatgacggtaaacgaagctcttaatgcggttttagccaaaa

800 847
ataaagataacgaacaatggggtataccggcaggattcagagggtaa

**Supplementary Figure 1: The location of IHF binding site, DnaA binding sites 1 and 2, A/T-rich in regions 1 and 2 within *repL* gene sequence.** The 847 bp *repL* gene contains an IHF binding site (in blue), DnaA binding sites (in green), A/T-rich in regions (AT1, AT2, in yellow), which might play an important role in RepL-mediated DNA replication process. The positions of first and last nucleotide of AT1, AT2, DnaA 1 and DnaA 2 in relation to *repL* coding sequence are shown as numbers.

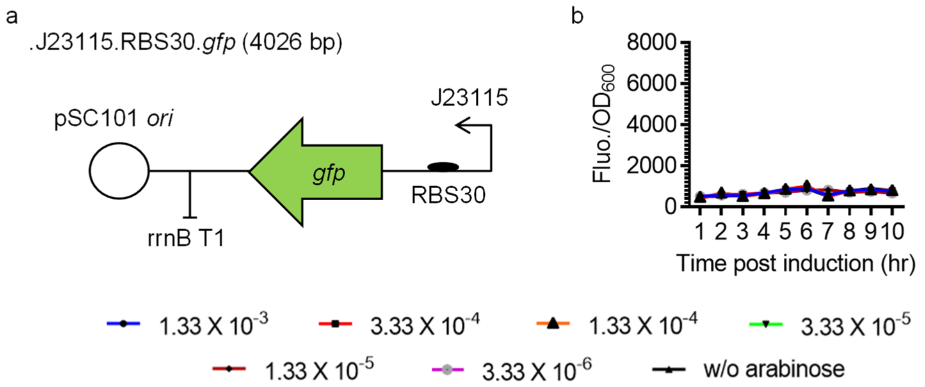


**Supplementary Figure 2: Arabinose induction did not increase the GFP output of a plasmid without *repL* gene. a)** Schematic diagram of a *gfp* reporter construct. *gfp* (in green) expression was controlled by a weak constitutive promoter Bba_J23115, with an artificial ribosomal binding site, RBS30. The plasmid confers ampicillin resistance and has a low copy number origin of replication, pSC101. **b)** The GFP fluorescence intensity per $OD_{600}$ (Fluo./$OD_{600}$) of *E. coli* TOP10 *gfp* transformants, at 1 to 10 hours post-induction (hpi) with $3.33 \times 10^{-6}$ M (purple line with grey circles), $1.33 \times 10^{-5}$ M (brown line with black diamonds), $3.33 \times 10^{-5}$ M (green line with black triangles), $1.33 \times 10^{-4}$ M (orange line with black triangles), $3.33 \times 10^{-4}$ M (red line with black squares), $1.33 \times 10^{-3}$ M of L-arabinose (blue line with black circles). GFP Fluo./$OD_{600}$ values of uninduced cells (w/o arabinose, in black line with black triangles) were added for comparison purposes. All GFP fluorescence intensity values were normalised to that of TOP10 transformants with an empty 4A3 plasmid without *gfp*. Induction assays were performed with 3 biological replicates and 4 technical replicates. Data were presented as mean ± SEM.

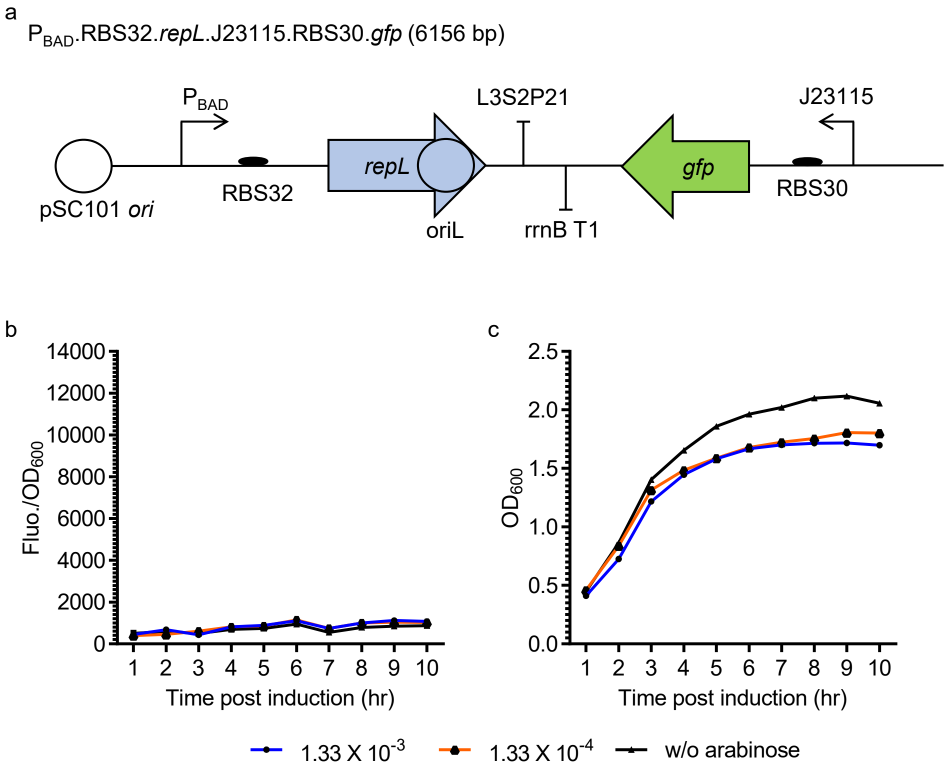


**Supplementary Figure 3: Replacing the *kilA* coding sequence with RBS32 disrupted the RepL-mediated signal amplification of a *gfp* reporter plasmid in *E. coli* TOP10. a)** Schematic diagram of a *gfp*.RBS32.*repL* reporter construct. *gfp* (in green) expression was controlled by a weak constitutive promoter

Bba_J23115, with an artificial ribosomal binding site, RBS30. The plasmid confers ampicillin resistance and has a low copy number origin of replication, pSC101. The *kilA* coding sequence was replaced with an artificial ribosomal binding site, RBS32. **b)** The GFP fluorescence intensity per $OD_{600}$ (Fluo./$OD_{600}$) of *E. coli* TOP10 *gfp*.RBS32.*repL* transformants, and **c)** the $OD_{600}$, at 1 to 10 hpi with $1.33 \times 10^{-3}$ M (blue line, black circles) and $1.33 \times 10^{-4}$ M (orange line with black triangles) arabinose induction. GFP Fluo./$OD_{600}$ values of uninduced cells (w/o arabinose, in black line with black triangles) were added for comparison purposes. All GFP fluorescence intensity values were normalised to that of TOP10 cells transformed with an empty plasmid without *gfp* and *repL* genes. Induction assays were performed with 3 biological replicates and 4 technical replicates. Data were presented as mean ± SEM.

a

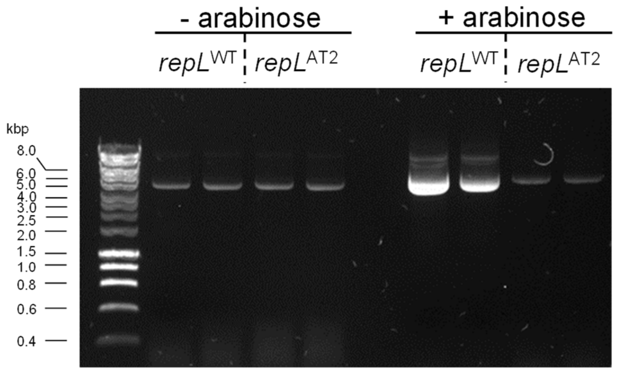


b

| | - arabinose | | | | + arabinose | | | |
|---|---|---|---|---|---|---|---|---|
| | *repL*^WT | | *repL*^AT2 | | *repL*^WT | | *repL*^AT2 | |
| $OD_{600}$ | 2.12 | 2.02 | 2.10 | 2.11 | 0.51 | 0.40 | 1.76 | 1.79 |
| GFP Fluo./$OD_{600}$ ($\times 10^3$) | 0.57 | 0.68 | 0.64 | 0.52 | 13.4 | 10.1 | 0.86 | 1.1 |
| Concentration of DNA (ng/μL) | 61 | 57 | 60 | 65 | 274 | 175 | 53 | 45 |
| Estimated copy number of plasmid per $OD_{600}$ ($10^{11}$) | 1.36 | 1.33 | 1.35 | 1.45 | 25.3 | 20.6 | 1.42 | 1.18 |

**Supplementary Figure 4: RepL-mediated, amplification of plasmid copy number *in cis*. a)** Image of a 1 % agarose gel, with miniprep product of *E. coli* TOP10 4A3.*gfp.repL*$^{WT}$ (*repL*$^{WT}$) and 4A3.*gfp.repL*$^{AT2}$ (*repL*$^{AT2}$) transformants, uninduced (-arabinose) or induced with $1.33 \times 10^{-4}$ M arabinose (+ arabinose) for 8 hours. 5 mL of TOP10 cultures were used for miniprep, and 5 μL of eluted DNA was used for agarose gel electrophoresis. **b)** Triplicates of the transformants (in 200 μL) were collected and the $OD_{600}$ as well as GFP fluorescence intensity (GFP Fluo.) were recorded using a CLARIOstar Plus BMG Microplate reader (BMG Labtech), with the average values shown in the table. Concentration of eluted DNA (total of approximately 30 μL) was determined using a DeNovix DS-11 Series Spectrophotometer. The results indicated a significant increase in copy number of *gfp.repL*$^{WT}$ plasmid at 8 hours post induction with arabinose induction when compared to uninduced state ($p < 0.0005$). Contrastingly, the copy number of *gfp.repL*$^{AT2}$ plasmid was not significantly increased upon arabinose induction, when compared to uninduced state ($p > 0.05$).

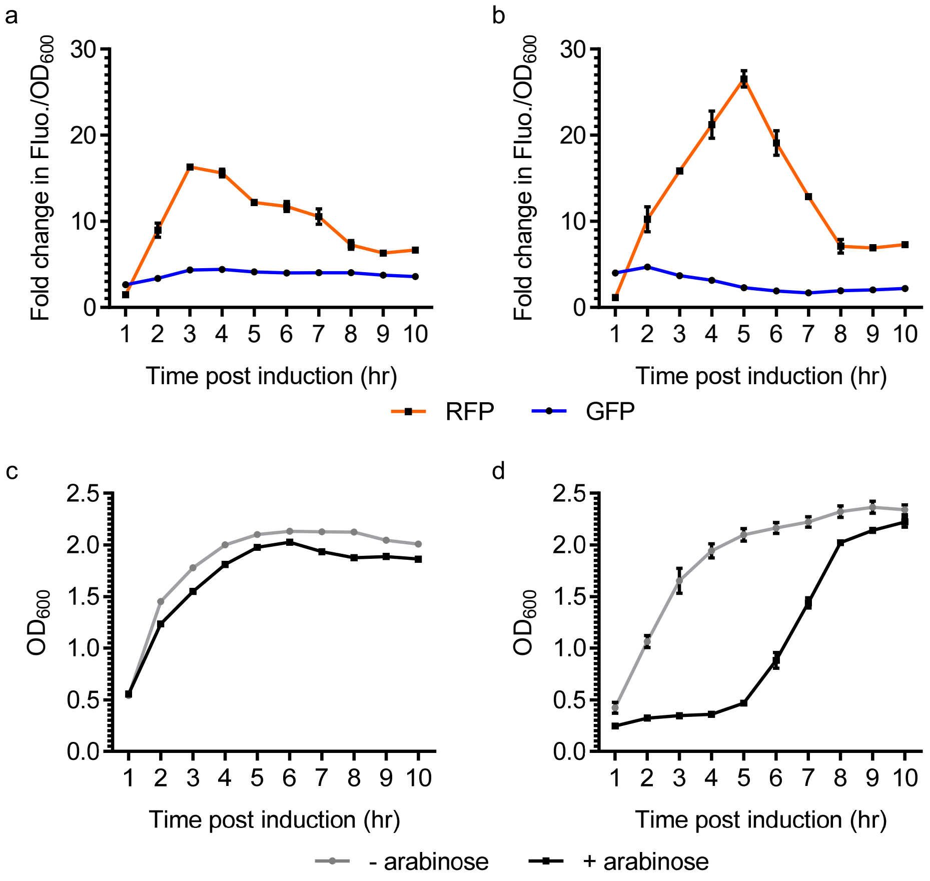


**Supplementary Figure 5: Arabinose induction of *repL* gene in two laboratory strains of *E. coli*.** The fold changes in GFP (in blue) and RFP (in orange) fluorescence intensity per $OD_{600}$ (Fluo./$OD_{600}$) of **a)** *E. coli* NCM3722 cells and **b)** BL21 cells transformed with the *gfp.repL*$^{WT}$ and *rfp*.nc-*repL*$^{WT}$ reporter plasmids at 1 to 10 hpi with 1.33 X $10^{-3}$ M of L-arabinose. The $OD_{600}$ of **c)** *E. coli* NCM3722 and **d)** BL21 transformants, at 1 to 10 hpi with (+ arabinose, in black) or without (- arabinose, in grey) 1.33 X $10^{-3}$ M of L-arabinose. Fold changes in the GFP and RFP Fluo./$OD_{600}$ values were calculated based on comparison between arabinose-induced and uninduced state. All GFP and RFP fluorescence intensity values were normalised to that of TOP10 cells transformed with empty plasmids without *gfp* and *repL* genes or without *rfp* gene and nc-*repL* sequence. Induction assays were performed with 3 biological replicates and 4 technical replicates. Data were presented as mean ± SEM.

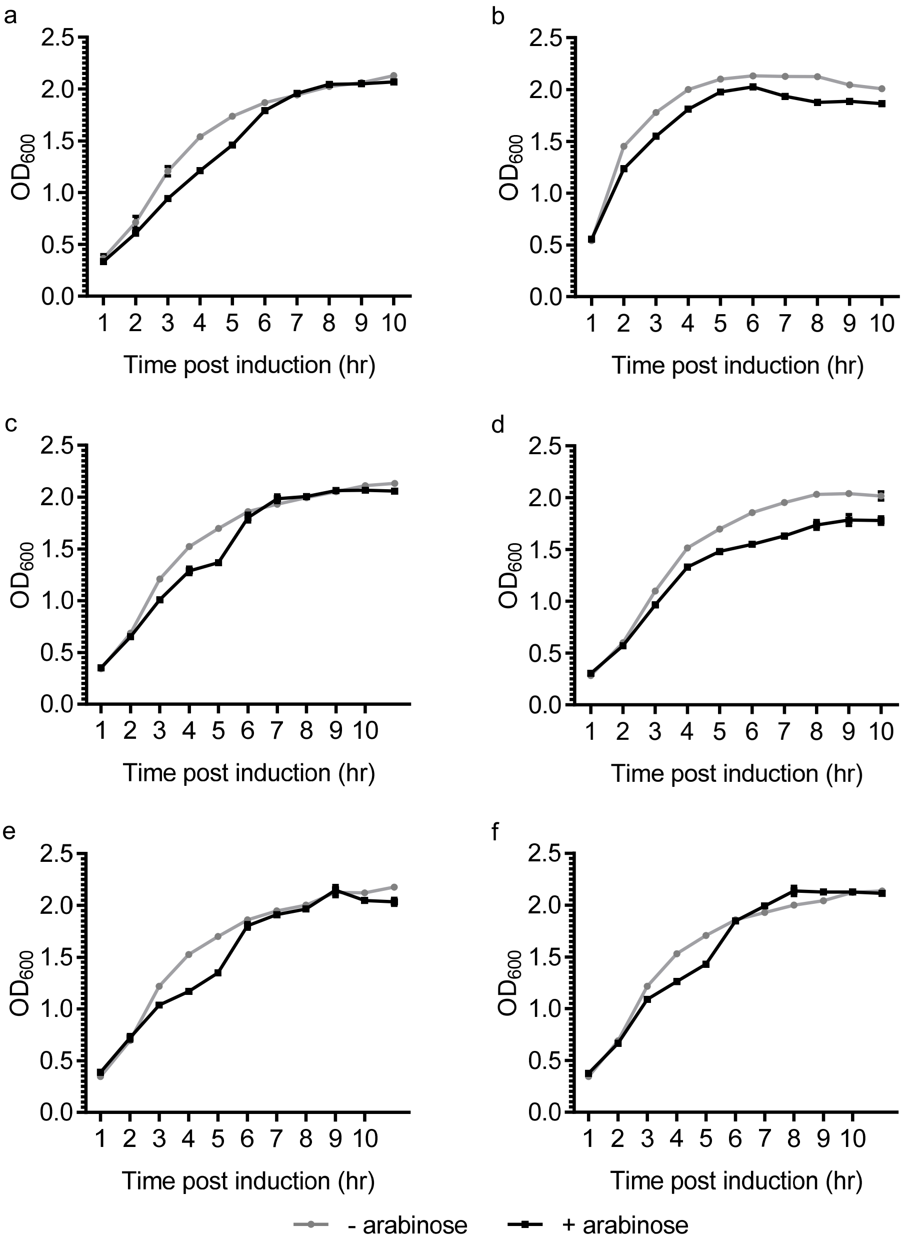


**Supplementary Figure 6: $OD_{600}$ of *E. coli* TOP10 transformants with truncated versions of non-protein coding *repL* constructs (nc-*repL*).** The $OD_{600}$ of TOP10 cells co-transformed a *gfp.repL*$^{AT2}$ plasmid and a **a)** full length **b)** truncation 1 (T1), **c)** truncation 2 (T2), **d)** truncation 3 (T3), **e)** truncation 4 (T4) or **f)** truncation 5 (T5) versions of a nc-*repL* construct at 1 to 10 hpi with (+ arabinose, in black) or without (-arabinose, in grey) 1.33 X $10^{-3}$ M of L-arabinose. Induction assays were performed with 3 biological replicates and 4 technical replicates. Data were presented as mean ± SEM.

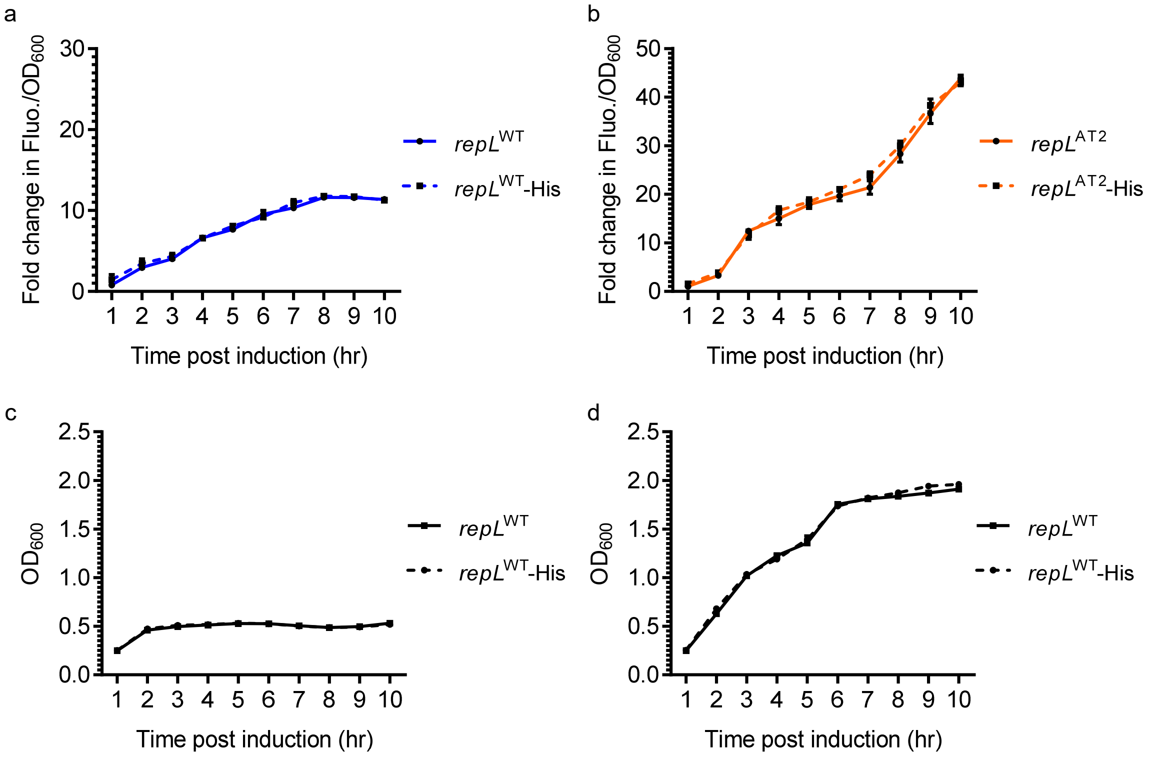


**Supplementary Figure 7: Presence of a PolyHis tag-encoding sequence at the C-terminus of *repL* gene did not significantly affect the *repL*-mediated signal amplification *in cis* and *in trans***. The fold changes in GFP (in blue) and RFP (in orange) fluorescence intensity per $OD_{600}$ (Fluo./$OD_{600}$) of *E. coli* TOP10 cells transformed with **a)** 4A3.*repL*$^{WT}$.*gfp* (blue solid line) or 4A3.*repL*$^{WT}$-His.*gfp* (blue dashed line), as well as co-transformed with **b)** 4A3.*repL*$^{AT2}$.*gfp* (orange solid line) or 4A3.*repL*$^{AT2}$-His.*gfp* (orange dashed line) plasmid and a RK2.nc-*repL*$^{WT}$.*rfp* plasmid. **c)** The $OD_{600}$ of 4A3.*repL*$^{WT}$.*gfp* (black solid line) or 4A3.*repL*$^{WT}$-His.*gfp* (black dashed line) transformants. **d)** The $OD_{600}$ of 4A3.*repL*$^{AT2}$.*gfp* (black solid line) or 4A3.*repL*$^{AT2}$-His.*gfp* (black dashed line) plasmid and a RK2.nc-*repL*$^{WT}$.*rfp* plasmid transformants. Fold changes in the GFP and RFP Fluo./$OD_{600}$ values were calculated based on comparison between arabinose-induced and uninduced state. All GFP and RFP fluorescence intensity values were normalised to that of TOP10 cells transformed with empty plasmids without *gfp* and *repL* genes or without *rfp* gene and nc-*repL* sequence. Induction assays were performed with 3 biological replicates and 4 technical replicates. Data were presented as mean ± SEM.

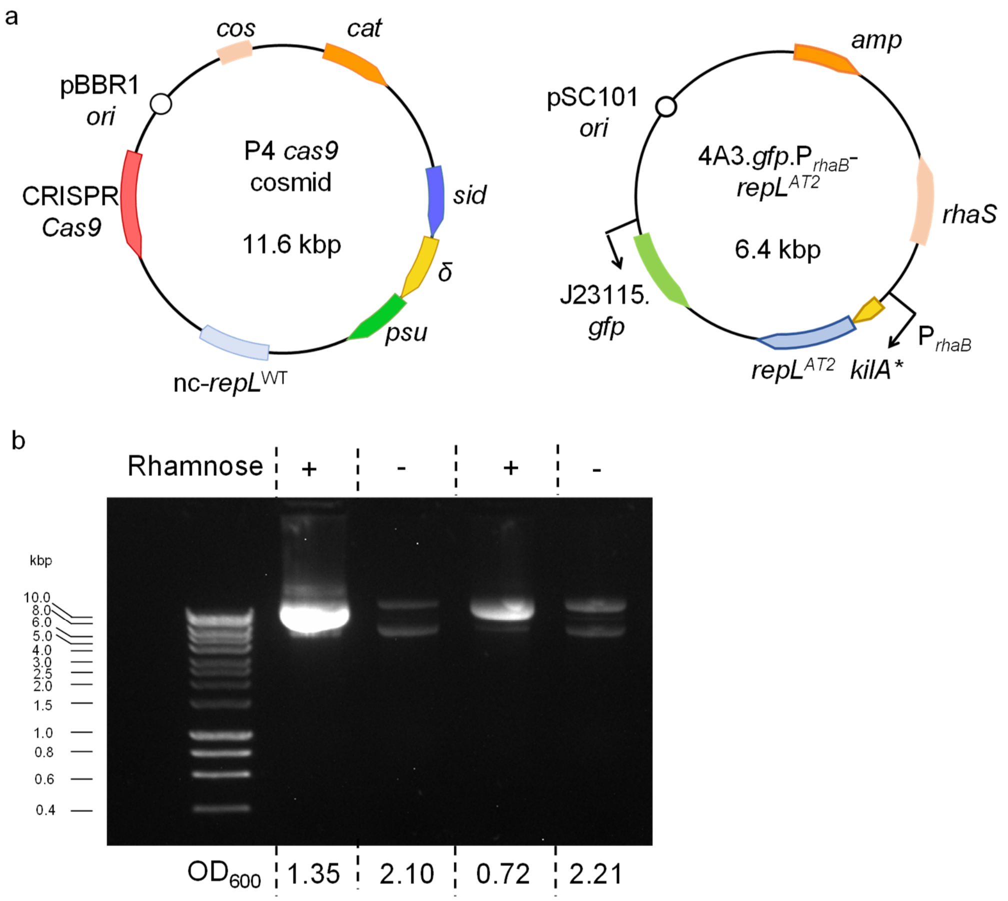


**Supplementary Figure 8: RepL-mediated, amplification of a cosmid copy number *in trans*. a)** Schematic diagram showing the 11.6 kbp P4 cosmid and a 6.4 kbp plasmid containing a nc-*repL*$^{WT}$ sequence and a *repL*$^{AT2}$ allele, respectively. The *repL*$^{AT2}$ expression was controlled by a rhamnose inducible, $P_{rhaB}$ promoter. The P4 cosmid contains a constitutively active, CRISPR Cas9 construct whose expression is regulated by $P_{cas9}$, the P4-derived *sid* operon consisting of *sid*, *δ* and *psu* genes (allowing the production of smaller size P4 capsid in an *E. coli* P2 lysogenic strain), and a chloramphenicol acetyltransferase gene (*cat*) as the selection marker. Rhamnose induction of *repL*$^{AT2}$ would promote DNA replication of the cosmid *in trans* but not *in cis*, which could potentially be distinguished by differences in intensity of DNA bands corresponding to the vectors. The *repL*$^{AT2}$ gene expression was placed under the regulation of $P_{rhaB}$, as the same promoter was involved in regulating the switch between lysogenic to lytic replication of an *E. coli* C-5545 Δcosδε P2 lysogen (described in Tridgett et al., 2021) therefore allowing an increase in P4 cosmid copy number and the packaging of this construct into phage particles. **b)** Image of a 1 % agarose gel, with miniprep product of *E. coli* TOP10 P4 nc-*repL*$^{WT}$ cosmid and *repL*$^{AT2}$ plasmid double transformants, uninduced (-) or induced with 0.2 % rhamnose (+) for 8 hours. 5 mL of TOP10 cultures were used for miniprep, and 5 µL of eluted DNA was used for agarose gel electrophoresis. 200 µL of the transformants were collected and the $OD_{600}$ values were recorded using a CLARIOstar Plus BMG Microplate reader (BMG Labtech). The $OD_{600}$ values were reported in the figure, below the gel image. A higher intensity of DNA band corresponding to P4 cosmid was observed upon rhamnose induction when compared to the DNA band corresponding to the *repL*$^{AT2}$ plasmid. The lower $OD_{600}$ values of induced transformants might be due to the increase in *cas9* and/or P4 *sid* genes copy number, whereby a high level of constitutive *cas9* gene expression and/or P4 *sid* operon gene expression could potentially have negatively impacted *E. coli* TOP10 growth.

**Supplementary Table 1:** Bacterial strains used in this study

| Registry | Description | Remarks/Source |
|---|---|---|
| *E. coli* TOP10 | Used for routine molecular cloning, induction of *repL* expression | Thermo Fisher Scientific, Waltham, MA, USA |
| *E. coli* P1 lysogen EMG16 | P1 lysogenic strain, used for molecular cloning of *repL* gene sequence | CGSC#: 4405 |
| *E. coli* NCM3722 | Induction of *repL* expression | CGSC#: 12355 |
| *E. coli* BL21 | Induction of *repL* expression | Thermo Fisher Scientific, Waltham, MA, USA |

**Supplementary Table 2:** Plasmids used in this study

| Registry | Description | Remarks/Source |
|---|---|---|
| 4A3.$P_{BAD}$.*repL*$^{WT}$.J23115.RBS30.*gfp* | Plasmid of pSC101 *ori*, conferring ampicillin resistance, *repL* gene expression under $P_{BAD}$, having weak and constitutive *gfp* expression | Plasmid generated in this study |
| 4A3.$P_{BAD}$.*repL*$^{AT1}$.J23115.RBS30.*gfp* | Plasmid having synonymous mutations introduced into AT1 region of *repL*. Identical features, otherwise, as compared to 4A3.$P_{BAD}$.*repL*$^{WT}$.J23115.RBS30.*gfp* | Plasmid generated in this study |
| 4A3.$P_{BAD}$.*repL*$^{D1}$.J23115.RBS30.*gfp* | Plasmid having synonymous mutations introduced into D1 region of *repL*. Identical features, otherwise, as compared to 4A3.$P_{BAD}$.*repL*$^{WT}$.J23115.RBS30.*gfp* | Plasmid generated in this study |
| 4A3.$P_{BAD}$.*repL*$^{AT2}$.J23115.RBS30.*gfp* | Plasmid having synonymous mutations introduced into AT2 region of *repL*. Identical features, otherwise, as compared to 4A3.$P_{BAD}$.*repL*$^{WT}$.J23115.RBS30.*gfp* | Plasmid generated in this study |
| 4A3.$P_{BAD}$.*repL*$^{D2}$.J23115.RBS30.*gfp* | Plasmid having synonymous mutations introduced into D2 region of *repL*. Identical features, otherwise, as compared to 4A3.$P_{BAD}$.*repL*$^{WT}$.J23115.RBS30.*gfp* | Plasmid generated in this study |
| P1 *cas9*-NT | P1 phagemid with constitutive *cas9* expression, tracrRNA and crRNA guide from *S. pyogenes* derived from pCas9 (Addgene plasmid #42876). Cas9 chromosomal-targeting effect is absent in *S. flexneri*, hence named non-targeting (NT). Used for molecular cloning of nc-*repL* sequence | Phagemid generated in our previous study (Huan et al., 2022) |
| RK2.nc-*repL*$^{WT}$.J23115.RBS30.*rfp* | Plasmid of RK2 *ori*, conferring kanamycin resistance, non-coding *repL* sequence (nc-*repL*) without promoter nor start codon which inhibits gene transcription and translation, having weak and constitutive *rfp* expression | Plasmid generated in this study |
| RK2.nc-*repL*$^{AT2}$.J23115.RBS30.*rfp* | Plasmid having synonymous mutations introduced into AT2 region of non-coding *repL* sequence. Identical features, otherwise, as compared to RK2.nc-*repL*$^{WT}$.J23115.RBS30.*rfp* | Plasmid generated in this study |

| | | |
|---|---|---|
| RK2.(T1)nc-*repL*$^{WT}$. J23115.RBS30.*rfp* | Plasmid having T1 truncated version of nc-*repL*. Identical features, otherwise, as compared to RK2.nc-*repL*$^{WT}$. J23115.RBS30.*rfp*. | Plasmid generated in this study |
| RK2.(T2)nc-*repL*$^{WT}$. J23115.RBS30.*rfp* | Plasmid having T2 truncated version of nc-*repL*. Identical features, otherwise, as compared to RK2.nc-*repL*$^{WT}$. J23115.RBS30.*rfp*. | Plasmid generated in this study |
| RK2.(T3)nc-*repL*$^{WT}$. J23115.RBS30.*rfp* | Plasmid having T3 truncated version of nc-*repL*. Identical features, otherwise, as compared to RK2.nc-*repL*$^{WT}$. J23115.RBS30.*rfp*. | Plasmid generated in this study |
| RK2.(T4)nc-*repL*$^{WT}$. J23115.RBS30.*rfp* | Plasmid having T4 Truncated version of nc-*repL*. Identical features, otherwise, as compared to RK2.nc-*repL*$^{WT}$. J23115.RBS30.*rfp*. | Plasmid generated in this study |
| RK2.(T5)nc-*repL*$^{WT}$. J23115.RBS30.*rfp* | Plasmid having T5 truncated version of nc-*repL*. Identical features, otherwise, as compared to RK2.nc-*repL*$^{WT}$. J23115.RBS30.*rfp*. | Plasmid generated in this study |
| 4A3.$P_{BAD}$.SL1 *repL*.J23115.RBS30.*gfp* | Plasmid having *SL1* mutation introduced into the AT2 region of *repL* gene sequence. Identical features, otherwise, as compared to 4A3.$P_{BAD}$.*repL*$^{WT}$.J23115.RBS30.*gfp.* | Plasmid generated in this study |
| 4A3.$P_{BAD}$.SL2 *repL*.J23115.RBS30.*gfp* | Plasmid having *SL2* mutation introduced into the AT2 region of *repL* gene sequence. Identical features, otherwise, as compared to 4A3.$P_{BAD}$.*repL*$^{WT}$.J23115.RBS30.*gfp.* | Plasmid generated in this study |
| 4A3.$P_{BAD}$.SL3 *repL*.J23115.RBS30.*gfp* | Plasmid having *SL3* mutation introduced into the AT2 region of *repL* gene sequence. Identical features, otherwise, as compared to 4A3.$P_{BAD}$.*repL*$^{WT}$.J23115.RBS30.*gfp.* | Plasmid generated in this study |
| 4A3.$P_{BAD}$.SL4 *repL*.J23115.RBS30.*gfp* | Plasmid having *SL4* mutation introduced into the AT2 region of *repL* gene sequence. Identical features, otherwise, as compared to 4A3.$P_{BAD}$.*repL*$^{WT}$.J23115.RBS30.*gfp.* | Plasmid generated in this study |
| 4A3.$P_{BAD}$.SL5 *repL*.J23115.RBS30.*gfp* | Plasmid having *SL5* mutation introduced into the AT2 region of *repL* gene sequence. Identical features, otherwise, as compared to 4A3.$P_{BAD}$.*repL*$^{WT}$.J23115.RBS30.*gfp.* | Plasmid generated in this study |
| 4A3.$P_{BAD}$.SL6 *repL*.J23115.RBS30.*gfp* | Plasmid having *SL6* mutation introduced into the AT2 region of *repL* gene sequence. Identical features, otherwise, as compared to 4A3.$P_{BAD}$.*repL*$^{WT}$.J23115.RBS30.*gfp.* | Plasmid generated in this study |
| 4A3.$P_{BAD}$.SL7 *repL*.J23115.RBS30.*gfp* | Plasmid having *SL7* mutation introduced into the AT2 region of *repL* gene sequence. Identical features, otherwise, as compared to 4A3.$P_{BAD}$.*repL*$^{WT}$.J23115.RBS30.*gfp.* | Plasmid generated in this study |

| | | |
|---|---|---|
| 4A3.$P_{BAD}$.SL8 *repL*.J23115.RBS30.*gfp* | Plasmid having *SL8* mutation introduced into the AT2 region of *repL* gene sequence. Identical features, otherwise, as compared to 4A3.$P_{BAD}$.*repL*$^{WT}$.J23115.RBS30.*gfp.* | Plasmid generated in this study |
| 4A3.$P_{BAD}$.SL9 *repL*.J23115.RBS30.*gfp* | Plasmid having *SL9* mutation introduced into the AT2 region of *repL* gene sequence. Identical features, otherwise, as compared to 4A3.$P_{BAD}$.*repL*$^{WT}$.J23115.RBS30.*gfp.* | Plasmid generated in this study |
| 4A3.$P_{BAD}$.SL10 *repL*.J23115.RBS30.*gfp* | Plasmid having *SL10* mutation introduced into the AT2 region of *repL* gene sequence. Identical features, otherwise, as compared to 4A3.$P_{BAD}$.*repL*$^{WT}$.J23115.RBS30.*gfp.* | Plasmid generated in this study |
| 4A3.$P_{BAD}$.SL11 *repL*.J23115.RBS30.*gfp* | Plasmid having *SL11* mutation introduced into the AT2 region of *repL* gene sequence. Identical features, otherwise, as compared to 4A3.$P_{BAD}$.*repL*$^{WT}$.J23115.RBS30.*gfp.* | Plasmid generated in this study |
| 4A3.$P_{BAD}$.SL1+2+3 *repL*.J23115.RBS30.*gfp* | Plasmid having *SL1, SL2* and *SL3* mutations introduced into the AT2 region of *repL* gene sequence. Identical features, otherwise, as compared to 4A3.$P_{BAD}$.*repL*$^{WT}$.J23115.RBS30.*gfp.* | Plasmid generated in this study |
| 4A3.$P_{BAD}$.SL4+5+6 *repL*.J23115.RBS30.*gfp* | Plasmid having *SL4, SL5* and *SL6* mutations introduced into the AT2 region of *repL* gene sequence. Identical features, otherwise, as compared to 4A3.$P_{BAD}$.*repL*$^{WT}$.J23115.RBS30.*gfp.* | Plasmid generated in this study |
| 4A3.$P_{BAD}$.SL7+8+9 *repL*.J23115.RBS30.*gfp* | Plasmid having *SL7, SL8* and *SL9* mutations introduced into the AT2 region of *repL* gene sequence. Identical features, otherwise, as compared to 4A3.$P_{BAD}$.*repL*$^{WT}$.J23115.RBS30.*gfp.* | Plasmid generated in this study |
| 4A3.$P_{BAD}$.SL1+2+3+4 *repL*.J23115.RBS30.*gfp* | Plasmid having *SL1, SL2, SL3* and *SL4* mutations introduced into the AT2 region of *repL* gene sequence. Identical features, otherwise, as compared to 4A3.$P_{BAD}$.*repL*$^{WT}$.J23115.RBS30.*gfp.* | Plasmid generated in this study |
| 4A3.$P_{BAD}$.SL8+9+10+11 *repL*.J23115.RBS30.*gfp* | Plasmid having *SL8, SL9, SL10* and *SL11* mutations introduced into the AT2 region of *repL* gene sequence. Identical features, otherwise, as compared to 4A3.$P_{BAD}$.*repL*$^{WT}$.J23115.RBS30.*gfp.* | Plasmid generated in this study |
| RK2.SL1 nc-*repL*. J23115.RBS30.*rfp* | Plasmid having *SL1* mutation introduced into the AT2 region of nc-*repL* gene sequence. Identical features, otherwise, as compared to RK2.nc-*repL*$^{WT}$. J23115.RBS30.*rfp*. | Plasmid generated in this study |
| RK2.SL2 nc-*repL*. J23115.RBS30.*rfp* | Plasmid having *SL2* mutation introduced into the AT2 region of nc-*repL* gene sequence. Identical features, otherwise, as compared to RK2.nc-*repL*$^{WT}$. J23115.RBS30.*rfp*. | Plasmid generated in this study |

| | | |
|---|---|---|
| RK2.SL3 nc-*repL*. J23115.RBS30.*rfp* | Plasmid having *SL3* mutation introduced into the AT2 region of nc-*repL* gene sequence. Identical features, otherwise, as compared to RK2.nc-*repL*$^{WT}$. J23115.RBS30.*rfp*. | Plasmid generated in this study |
| RK2.SL4 nc-*repL*. J23115.RBS30.*rfp* | Plasmid having *SL4* mutation introduced into the AT2 region of nc-*repL* gene sequence. Identical features, otherwise, as compared to RK2.nc-*repL*$^{WT}$. J23115.RBS30.*rfp*. | Plasmid generated in this study |
| RK2.SL5 nc-*repL*. J23115.RBS30.*rfp* | Plasmid having *SL5* mutation introduced into the AT2 region of nc-*repL* gene sequence. Identical features, otherwise, as compared to RK2.nc-*repL*$^{WT}$. J23115.RBS30.*rfp*. | Plasmid generated in this study |
| RK2.SL6 nc-*repL*. J23115.RBS30.*rfp* | Plasmid having *SL6* mutation introduced into the AT2 region of nc-*repL* gene sequence. Identical features, otherwise, as compared to RK2.nc-*repL*$^{WT}$. J23115.RBS30.*rfp*. | Plasmid generated in this study |
| RK2.SL7 nc-*repL*. J23115.RBS30.*rfp* | Plasmid having *SL7* mutation introduced into the AT2 region of nc-*repL* gene sequence. Identical features, otherwise, as compared to RK2.nc-*repL*$^{WT}$. J23115.RBS30.*rfp*. | Plasmid generated in this study |
| RK2.SL8 nc-*repL*. J23115.RBS30.*rfp* | Plasmid having *SL8* mutation introduced into the AT2 region of nc-*repL* gene sequence. Identical features, otherwise, as compared to RK2.nc-*repL*$^{WT}$. J23115.RBS30.*rfp*. | Plasmid generated in this study |
| RK2.SL9 nc-*repL*. J23115.RBS30.*rfp* | Plasmid having *SL9* mutation introduced into the AT2 region of nc-*repL* gene sequence. Identical features, otherwise, as compared to RK2.nc-*repL*$^{WT}$. J23115.RBS30.*rfp*. | Plasmid generated in this study |
| RK2.SL10 nc-*repL*. J23115.RBS30.*rfp* | Plasmid having *SL10* mutation introduced into the AT2 region of nc-*repL* gene sequence. Identical features, otherwise, as compared to RK2.nc-*repL*$^{WT}$. J23115.RBS30.*rfp*. | Plasmid generated in this study |
| RK2.SL11 nc-*repL*. J23115.RBS30.*rfp* | Plasmid having *SL11* mutation introduced into the AT2 region of nc-*repL* gene sequence. Identical features, otherwise, as compared to RK2.nc-*repL*$^{WT}$. J23115.RBS30.*rfp*. | Plasmid generated in this study |
| RK2.SL2+5 nc-*repL*. J23115.RBS30.*rfp* | Plasmid having *SL2* and *SL5* mutations introduced into the AT2 region of nc-*repL* gene sequence. Identical features, otherwise, as compared to RK2.nc-*repL*$^{WT}$. J23115.RBS30.*rfp*. | Plasmid generated in this study |
| RK2.SL2+6 nc-*repL*. J23115.RBS30.*rfp* | Plasmid having *SL2* and *SL6* mutations introduced into the AT2 region of nc-*repL* gene sequence. Identical features, otherwise, as compared to RK2.nc-*repL*$^{WT}$. J23115.RBS30.*rfp*. | Plasmid generated in this study |

| | | |
|---|---|---|
| RK2.SL2+7 nc-*repL*. J23115.RBS30.*rfp* | Plasmid having *SL2* and *SL7* mutations introduced into the AT2 region of nc-*repL* gene sequence. Identical features, otherwise, as compared to RK2.nc-*repL*$^{WT}$. J23115.RBS30.*rfp*. | Plasmid generated in this study |
| RK2.SL1+2+3 nc-*repL*. J23115.RBS30.*rfp* | Plasmid having *SL1, SL2* and *SL3* mutations introduced into the AT2 region of nc-*repL* gene sequence. Identical features, otherwise, as compared to RK2.nc-*repL*$^{WT}$. J23115.RBS30.*rfp*. | Plasmid generated in this study |
| RK2.SL4+5+6 nc-*repL*. J23115.RBS30.*rfp* | Plasmid having *SL4, SL5* and *SL6* mutations introduced into the AT2 region of nc-*repL* gene sequence. Identical features, otherwise, as compared to RK2.nc-*repL*$^{WT}$. J23115.RBS30.*rfp*. | Plasmid generated in this study |
| RK2.SL1+2+7+8 nc-*repL*. J23115.RBS30.*rfp* | Plasmid having *SL1, SL2, SL7, SL8* mutations introduced into the AT2 region of nc-*repL* gene sequence. Identical features, otherwise, as compared to RK2.nc-*repL*$^{WT}$. J23115.RBS30.*rfp*. | Plasmid generated in this study |
| RK2.SL8+9+10+11 nc-*repL*. J23115.RBS30.*rfp* | Plasmid having *SL8, SL9, SL10, SL11* mutations introduced into the AT2 region of nc-*repL* gene sequence. Identical features, otherwise, as compared to RK2.nc-*repL*$^{WT}$. J23115.RBS30.*rfp*. | Plasmid generated in this study |
| RK2.P*asrR*.*repL*$^{AT2}$ | Plasmid of RK2 *ori*, conferring kanamycin resistance, providing *repL* gene expression in the presence of arsenite under the regulation of $P_{asrR}$. Synonymous mutations introduced into *repL* gene sequence to inhibit RepL-mediated DNA replication *in cis* | Plasmid generated by Dr. Xinyi Wan of Wang's lab |
| pSC101.J23109-*asrR*.$P_{asrR}$-*hprR.hprS*.$P_{hprLE}$-*gfp*.nc-*repL*$^{WT}$ | Plasmid of pSC101 *ori*, conferring ampicillin resistance, providing constitutive *asR* gene expression under the regulation of J23109. *hprR* and *hrpS* genes expression were regulated by $P_{asR}$, providing arsenite-inducible gene expression. The proteins in turn induce the expression of *gfp*. Non-coding *repL* (nc-*repL*) sequence acts as recognition site for RepL protein, allowing RepL-mediated DNA replication *in trans* | Plasmid generated by Dr. Xinyi Wan of Wang's lab |
| pSC101.J23109-*asrR*.$P_{asrR}$-*hprR.hprS*.$P_{hprLE}$-*gfp*.SL4 nc-*repL* | Plasmid having *SL4* mutation introduced into nc-*repL* sequence. Identical features, otherwise, as compared to pSC101.J23109-*asrR*.$P_{asrR}$-*hprR.hprS*.$P_{hprLE}$-*gfp*.nc-*repL* | Plasmid generated in this study |
| pSC101.J23109-*asrR*.$P_{asrR}$-*hprR.hprS*.$P_{hprLE}$-*gfp*.SL8 nc-*repL* | Plasmid having *SL8* mutation introduced into nc-*repL* sequence. Identical features, otherwise, as compared to pSC101.J23109-*asrR*.$P_{asrR}$-*hprR.hprS*.$P_{hprLE}$-*gfp*.nc-*repL* | Plasmid generated in this study |
| pSC101.J23109-*asrR*.$P_{asrR}$-*hprR.hprS*.$P_{hprLE}$-*gfp*.SL2 nc-*repL* | Plasmid having *SL2* mutation introduced into nc-*repL* sequence. Identical features, otherwise, as compared to pSC101.J23109-*asrR*.$P_{asrR}$-*hprR.hprS*.$P_{hprLE}$-*gfp*.nc-*repL* | Plasmid generated in this study |

| | | |
|---|---|---|
| pSC101.J23109-*asrR*.$P_{asrR}$-*hprR.hprS*.$P_{hprLE}$-*gfp*.SL2+7 nc-*repL* | Plasmid having *SL2* and *SL7* mutations introduced into nc-*repL* sequence. Identical features, otherwise, as compared to pSC101.J23109-*asrR*.$P_{asrR}$-*hprR.hprS*.$P_{hprLE}$-*gfp*.nc-*repL* | Plasmid generated in this study |
| pSC101.J23109-*asrR*.$P_{asrR}$-*hprR.hprS*.$P_{hprLE}$-*gfp*.SL4+5+6 nc-*repL* | Plasmid having *SL4, SL5* and *SL6* mutations introduced into nc-*repL* sequence. Identical features, otherwise, as compared to pSC101.J23109-*asrR*.$P_{asrR}$-*hprR.hprS*.$P_{hprLE}$-*gfp*.nc-*repL* | Plasmid generated in this study |
| pSC101.J23109-*asrR*.$P_{asrR}$-*hprR.hprS*.$P_{hprLE}$-*gfp*.nc-$repL^{AT2}$ | Plasmid having *AT2* mutations introduced into nc-*repL* sequence. Identical features, otherwise, as compared to pSC101.J23109-*asrR*.$P_{asrR}$-*hprR.hprS*.$P_{hprLE}$-*gfp*.nc-*repL* | Plasmid generated in this study |
| 4A3. J23115.RBS30.*gfp* | Plasmid of pSC101 *ori*, conferring ampicillin resistance, having weak and constitutive *gfp* expression (*gfp*-only plasmid) | Plasmid generated in this study |
| 4A3.$P_{BAD}$.RBS32.$repL^{WT}$. J23115.RBS30.*gfp* | Plasmid of pSC101 *ori*, conferring ampicillin resistance, *repL* gene expression under $P_{BAD}$ and RBS32, having weak and constitutive *gfp* expression | Plasmid generated in this study |
| 4A3 empty plasmid | Plasmid of pSC101 *ori*, conferring ampicillin resistance (empty plasmid), used for normalisation of data | Plasmid generated in this study |
| RK2 empty plasmid | Plasmid of RK2 *ori*, conferring kanamycin resistance (empty plasmid), used for normalisation of data | Plasmid generated in this study |

**Supplementary Table 3:** Primers used for mutagenesis of *repL* and nc-*repL* sequences

| Name | Sequence (5' to 3')[a] | Remarks |
|---|---|---|
| repL-f | gtacctgtcgcggcaacgcgctaa | Molecular cloning of *kilA.repL* sequence from *E. coli* EMG16 |
| repL-r | ttattaccctctgaatcctgccggtatacc | Molecular cloning of *kilA.repL* sequence from *E. coli* EMG16 |
| nc-repL-f | taaagcgattttactttttctggc | Primer to remove start codon of *repL* |
| nc-repL-r | actagtcgccagggttttcccagtcacgacttacc | Primer to assemble nc-*repL* in *rfp* plasmid, used with nc-repL-f |
| AT1-1 | gcgaagacgctggcgctgttcaactttttatccacacccccatgtc | Introducing mutations in AT1 region of *repL* |
| AT1-2 | gttgaacagcgccagcgtcttcgcgagaacagctttaaccttggttgc | Introducing mutations in AT1 region of *repL* |
| DnaA1-1 | cattggctttatttaatttcctgtccacgcccccatgtcaaaatgatac | Introducing mutations in DnaA binding site 1 of *repL* |
| DnaA1-2 | cgtggacaggaaattaaataaagccaatgtcttagcgagaacagc | Introducing mutations in DnaA binding site 1 of *repL* |
| AT2-1 | caagaagagccaggtgaagaagacgaagcgtagcgtttccggcgg tgccggaacaac | Introducing mutations in AT2 region of *repL* |
| AT2-2 | cgtcttcttcacctggctcttcttgttcttgatcgccacgtcatcctgacag | Introducing mutations in AT2 region of *repL* |
| DnaA2-1 | gcaaaggctgacaatctgcggctgtccaagaaacgcac | Introducing mutations in DnaA binding site 2 of *repL* |
| DnaA2-2 | tcttggacagccgcagattgtcagcctttgcttttgc | Introducing mutations in DnaA binding site 2 of *repL* |
| nc-repL T1-r | cgtgaacgtcgcgctaacctggtcgtgactgggaaaaccctggcg | Primer to construct T1 nc-*repL*, used with nc-repL-f as forward primer |
| nc-*repL* T2-f | ccagtcacgactttttgaggctggttgttccggc | Primer to construct T2 nc-*repL* |
| nc-repL T2-r | tgtaaaccggtagaattcgcggccgcttctagag | Primer to construct T2 nc-*repL* |
| nc-repL T3-f | agaagcggccgcgaattctgacttcatggatcgctaagg | Primer to construct T3 nc-*repL*, used with nc-repL-r as reverse primer |
| nc-repL T4-f | ctctagaagcggccgcgaattcgctaagacattggctttatt | Primer to construct T4 nc-*repL*, used with nc-repL-r as reverse primer |
| nc-repL T5-f | cgccagggttttcccagtcacgacccggcaccgccggaaactgatctt | Primer to construct T5 nc-*repL* |

| | | |
|---|---|---|
| nc-repL T5-r | actctagaagcggccgcgaattcccccctcccccctgtcaggatgacgtggcaataaagaataagaagtcacaagttaaaaaaaca | Primer to construct T5 nc-*repL* |
| SL1 | cccctgtcaggatgacgtggcGataaagaataagaagtcacaagttaaaaaaacaaaaagatcagtttccggcggtgccggaaca | Primer to introduce SL1 mutation into *repL* sequence, used with repL-r |
| SL2 | cccctgtcaggatgacgtggcaatCaagaataagaagtcacaagttaaaaaaacaaaaagatcagtttccggcggtgccggaaca | Primer to introduce SL2 mutation into *repL* sequence, used with repL-r or with nc-repL-r |
| SL3 | cccctgtcaggatgacgtggcaataaagaaCaagaagtcacaagttaaaaaaacaaaaagatcagtttccggcggtgccggaaca | Primer to introduce SL3 mutation into *repL* sequence, used with repL-r or with nc-repL-r |
| SL4 | cccctgtcaggatgacgtggcaataaagaataagaagAGCcaagttaaaaaaacaaaaagatcagtttccggcggtgccggaaca | Primer to introduce SL4 mutation into *repL* sequence, used with repL-r or with nc-repL-r |
| SL5 | cccctgtcaggatgacgtggcaataaagaataagaagtcacaGgttaaaaaaacaaaaagatcagtttccggcggtgccggaaca | Primer to introduce SL5 mutation into *repL* sequence, used with repL-r or with nc-repL-r |
| SL6 | cccctgtcaggatgacgtggcaataaagaataagaagtcacaagtGaaaaaaacaaaaagatcagtttccggcggtgccggaaca | Primer to introduce SL6 mutation into *repL* sequence, used with repL-r or with nc-repL-r |
| SL7 | cccctgtcaggatgacgtggcaataaagaataagaagtcacaagttaaGaaaacaaaaagatcagtttccggcggtgccggaaca | Primer to introduce SL7 mutation into *repL* sequence, used with repL-r or with nc-repL-r |
| SL8 | cccctgtcaggatgacgtggcaataaagaataagaagtcacaagttaaaaaGacaaaaagatcagtttccggcggtgccggaaca | Primer to introduce SL8 mutation into *repL* sequence, used with repL-r or with nc-repL-r |
| SL9 | cccctgtcaggatgacgtggcaataaagaataagaagtcacaagttaaaaaaacGaaaagatcagtttccggcggtgccggaaca | Primer to introduce SL9 mutation into *repL* sequence, used with repL-r or with nc-repL-r |
| SL10 | cccctgtcaggatgacgtggcaataaagaataagaagtcacaagttaaaaaaacaaaGCgatcagtttccggcggtgccggaaca | Primer to introduce SL10 mutation into *repL* sequence, used with repL-r or with nc-repL-r |
| SL11 | cccctgtcaggatgacgtggcaataaagaataagaagtcacaagttaaaaaaacaaaaagTAGCgtttccggcggtgccggaaca | Primer to introduce SL11 mutation into *repL* sequence, used with repL-r or with nc-repL-r |
| SL2+5 | cccctgtcaggatgacgtggcaatCaagaataagaagtcacaGgttaaaaaaacaaaaagatcagtttccggcggtgccggaaca | Primer to introduce SL2+5 mutation into *repL* sequence, used with repL-r or with nc-repL-r |

| | | |
|---|---|---|
| SL2+6 | cccctgtcaggatgacgtggcaatCaagaataagaagtcacaagtGaaaaaaacaaaaagatcagtttccggcggtgccggaaca | Primer to introduce SL2+6 mutation into *repL* sequence, used with repL-r or with nc-repL-r |
| SL2+7 | cccctgtcaggatgacgtggcaatCaagaataagaagtcacaagttaaGaaaacaaaaagatcagtttccggcggtgccggaaca | Primer to introduce SL2+7 mutation into *repL* sequence, used with repL-r or with nc-repL-r |
| SL1+2+3 | cccctgtcaggatgacgtggcGatCaagaaCaagaagtcacaagttaaaaaaacaaaaagatcagtttccggcggtgccggaaca | Primer to introduce SL1+2+3 mutation into *repL* sequence, used with repL-r or with nc-repL-r |
| SL4+5+6 | cccctgtcaggatgacgtggcaataaagaataagaagAGCcaGgtGaaaaaaacaaaaagatcagtttccggcggtgccggaaca | Primer to introduce SL4+5+6 mutation into *repL* sequence, used with repL-r or with nc-repL-r |
| SL7+8+9 | cccctgtcaggatgacgtggcaataaagaataagaagtcacaagttaaGaaGacGaaaagatcagtttccggcggtgccggaaca | Primer to introduce SL7+8+9 mutation into *repL* sequence, used with repL-r or with nc-repL-r |
| SL1+2+3+4 | cccctgtcaggatgacgtggcGatCaagaaCaagaagAGCcaagttaaaaaaacaaaaagatcagtttccggcggtgccggaaca | Primer to introduce SL1+2+3+4 mutation into *repL* sequence, used with repL-r or with nc-repL-r |
| SL1+2+7+8 | cccctgtcaggatgacgtggcGatCaagaataagaagtcacaagttaaGaaGacaaaaagatcagtttccggcggtgccggaaca | Primer to introduce SL1+2+7+8 mutation into *repL* sequence, used with repL-r or with nc-repL-r |
| SL8+9+10+11 | cccctgtcaggatgacgtggcaataaagaataagaagtcacaagttaaaaaGacGaaGCgTAGCgtttccggcggtgccggaaca | Primer to introduce SL8+9+10+11 mutation into *repL* sequence, used with repL-r or with nc-repL-r |

**Supplementary Tables 4:** DNA sequences of plasmids and constructs

*repL* construct*, kilA* sequence in orange, *repL* sequence in blue

tgtacctgtcgcggcaacgcgctaacagacgtagtaagaaccaccagcattgta
atgctggctaaagtcactttcctgagctgtataacgatgagcgattttactttttctggctatgaattggcctgctttgtaacacactccggtctatcccgtagcgccgggcatatcctgtcgcaatgtg
caaatctcgcggcaacaaccagtgaatacttcattcacaagcctcaccgcctgatcgcggcagaaactggttatagccaatcaaccgtcgttcgtgcattccgtgaagctgtaaacaaagg
aattctgtctgtagagattgttatcggcgatcaccgtgaacgtcgcgctaacctgtaccggtttacaccatcctttttggccttcgcacaacaagccaaaaatgcgctgatagaaagcaaattaa
agatctcttcagcggcaaccaaggttaaagctgttctcgctaagacattggctttatttaatttttatccacacccccatgtcaaaatgatacccctcccccctgtcaggatgacgtggcaataaa
gaataagaagtcacaagttaaaaaacaaaaagatcagtttccggcggtgccggaacaaccagcctcaaaaaattgacttcatggatcgctaaggcaaaagcaaaggctgacaatctg
cggttatccaaaaaacgcactcaaaaacatgagttcaagcagaaagtagaggcggctgcgcggaaatatgcttacctgaagaacaagcgttcgcctgatattggcgggatatcaaacttc
gataacctaccgcattgcatgacggtaaacgaagctcttaatgcggttttagccaaaaataaagataacgaacaatggggtataccggcaggattcagagggtaat

P~BAD~.*araC* construct, P~BAD~ in green, *araC* in blue

ttatgacaacttgacggctacatcattcactttttcttcacaaccggcacgaaactcgctcgggctggccccggtgcattttttaaatactcgcgagaaatagagttgatcgt
caaaaccaacattgcgaccgacggtggcgataggcatccgggtagtgctcaaaagcagcttcgcctgactaatgcgttggtcctcgcgccagcttaagacgctaatc
cctaactgctggcggaaaagatgtgacagacgcgacggcgacaagcaaacatgctgtgcgacgctggcgatatcaaaattgctgtctgccaggtgatcgctgatgta
ctgacaagcctcgcgtacccgattatccatcggtggatggagcgactcgttaatcgcttccatgcgccgcagtaacaattgctcaagcagatttatcgccagcagctcc
gaatagcgcccttccccttgcccggcgttaatgatttgcccaaacaggtcgctgaaatgcggctggtgcgcttcatccgggcgaaagaaacccgtattggcaaatattg
acggccagttaagccattcatgccagtaggcgcgcggacgaaagtaaacccactggtgataccattcgcgagcctccggatgacgaccgtagtgatgaatctctcct
ggcgggaacagcaaaatatcacccggtcggcagacaaattctcgtccctgatttttcaccaccccctgaccgcgaatggtgagattgagaatataacctttcattccca
gcggtcggtcgataaaaaaatcgagataaccgttggcctcaatcggcgttaaacccgccaccagatgggcgttaaacgagtatcccggcagcaggggatcattttgc
gcttcagccatacttttcatactcccaccattcagagaagaaaccaattgtccatattgcatcagacattgccgtcactgcgtcttttactggctcttctcgctaacccaaccg
gtaaccccgcttattaaaagcattctgtaacaaagcgggaccaaagccatgacaaaaacgcgtaacaaaagtgtctataatcacggcagaaaagtccacattgatt
atttgcacggcgtcacactttgctatgccatagcatttttatccataagattagcggatcctacctgacgctttttatcgcaactctctactgtttctccat

The J23115.RBS30.*gfp* construct, J23115 in lower cases alphabets, RBS30 in orange, *gfp* in green

tttatagctagctcagcccttggtacaatgctagcTCTAGAGATTAAAGAGGAGAAATACTAGATGCGTAAAGGAGAAGAACTTTTC
ACTGGAGTTGTCCCAATTCTTGTTGAATTAGATGGTGATGTTAATGGGCACAAATTTTCTGTCAGTGGAGAGGGTG
AAGGTGATGCAACATACGGAAAACTTACCCTTAAATTTATTTGCACTACTGGAAAACTACCTGTTCCATGGCCAAC
ACTTGTCACTACTTTCGGTTATGGTGTTCAATGCTTTGCGAGATACCCAGATCATATGAAACAGCATGACTTTTTCA
AGAGTGCCATGCCCGAAGGTTATGTACAGGAAAGAACTATATTTTTCAAAGATGACGGGAACTACAAGACACGTG
CTGAAGTCAAGTTTGAAGGTGATACCCTTGTTAATAGAATCGAGTTAAAAGGTATTGATTTTAAAGAAGATGGAAA
CATTCTTGGACACAAATTGGAATACAACTATAACTCACACAATGTATACATCATGGCAGACAAACAAAAGAATGGA
ATCAAAGTTAACTTCAAAATTAGACACAACATTGAAGATGGAAGCGTTCAACTAGCAGACCATTATCAACAAAATA
CTCCAATTGGCGATGGCCCTGTCCTTTTACCAGACAACCATTACCTGTCCACACAATCTGCCCTTTCGAAAGATCC
CAACGAAAAGAGAGACCACATGGTCCTTCTTGAGTTTGTAACAGCTGCTGGGATTACACATGGCATGGATGAACT
ATACAAATAA

4A3 plasmid backbone, with *amp* gene in blue

ttcttagacgtcaggtggcaccatgggaaatgtgcgcggaacccctatttgtttatttttctaaatacattcaaatatgtatccgctcatgagacaataaccctgataaatgcttcaataatattgaa
aaaggaagagtatgagtattcaacatttccgtgtcgcccttattcccttttttgcggcattttgccttcctgtttttgctcacccagaaacgctggtgaaagtaaaagatgctgaagatcagttgggtg
cacgagtgggttacatcgaactggatctcaacagcggtaagatccttgagagttttcgccccgaagaacgttttccaatgatgagcacttttaaagttctgctatgtggcgcggtattatcccgtat
tgacgccgggcaagagcaactcggtcgccgcatacactattctcagaatgacttggttgagtactcaccagtcacagaaaagcatcttacggatggcatgacagtaagagaattatgcagt
gctgccataaccatgagtgataacactgcggccaacttacttctgacaacgatcggaggaccgaaggagctaaccgcttttttgcacaacatgggggatcatgtaactcgccttgatcgttgg
gaaccggagctgaatgaagccataccaaacgacgagcgtgacaccacgatgcctgtagcaatggcaacaacgttgcgcaaactattaactggcgaactacttactctagcttcccggca
acaattaatagactggatggaggcggataaagttgcaggaccacttctgcgctcggcccttccggctggctggtttattgctgataaatctggagccggtgagcgtgggtctcgcggtatcattg
cagcactggggccagatggtaagccctcccgtatcgtagttatctacacgacggggagtcaggcaactatggatgaacgaaatagacagatcgctgagataggtgcctcactgattaagc
attggtaactgtcagaccaagtttacgagctcgcttggactcctgttgatagatccagtaatgacctcagaactccatctggatttgttcagaacgctcggttgccgccgggcgtttttattggtga
gaatccaagcactagggacagtaagacgggtaagcctgttgatgataccgctgccttactgggtgcattagccagtctgaatgacctgtcacgggataatccgaagtggtcagactggaaa
atcagagggcaggaactgctgaacagcaaaaagtcagatagcaccacatagcagacccgccataaaacgccctgagaagcccgtgacgggcttttcttgtattatgggtagtttccttgca
tgaatccataaaaggcgcctgtagtgccatttacccccattcactgccagagccgtgagcgcagcgaactgaatgtcacgaaaaagacagcgactcaggtgcctgatggtcggagacaa
aaggaatattcagcgatttgcccgagcttgcgagggtgctacttaagcctttagggttttaaggtctgttttgtagaggagcaaacagcgtttgcgacatccttttgtaatactgcggaactgacta
aagtagtgagttatacacagggctgggatctattctttttatctttttttattctttctttattctataaattataaccacttgaatataaacaaaaaaaacacacaaaggtctagcggaatttacagagg
gtctagcagaatttacaagttttccagcaaaggtctagcagaatttacagatacccacaactcaaaggaaaaggacatgtaattatcattgactagcccatctcaattggtatagtgattaaaat
cacctagaccaattgagatgtatgtctgaattagttgttttcaaagcaaatgaactagcgattagtcgctatgacttaacggagcatgaaaccaagctaattttatgctgtgtggcactactcaac
cccacgattgaaaaccctacaaggaaagaacggacggtatcgttcacttataaccaatacgctcagatgatgaacatcagtagggaaaatgcttatggtgtattagctaaagcaaccaga
gagctgatgacgagaactgtggaaatcaggaatcctttggttaaaggctttgagattttccagtggacaaactatgccaagttctcaagcgaaaaattagaattagtttttagtgaagagatatt
gccttatcttttccagttaaaaaaattcataaaatataatctggaacatgttaagtcttttgaaaacaaatactctatgaggatttatgagtggttattaaaagaactaacacaaaagaaaactcac
aaggcaaatatagagattagccttgatgaatttaagttcatgttaatgcttgaaaataactaccatgagtttaaaaggcttaaccaatgggttttgaaaccaataagtaaagatttaaacacttac
agcaatatgaaattggtggttgataagcgaggccgcccgactgatacgttgattttccaagttgaactagatagacaaatggatctcgtaaccgaacttgagaacaaccagataaaaatga
atggtgacaaaataccaacaaccattacatcagattcctacctacgtaacggactaagaaaaacactacacgatgctttaactgcaaaaattcagctcaccagttttgaggcaaaatttttga
gtgacatgcaaagtaagcatgatctcaatggttcgttctcatggctcacgcaaaaacaacgaaccacactagagaacatactggctaaatacggaaggatctgaggttcttatggctcttgta
tctatcagtgaagcatcaagactaacaaacaaaagtagaacaactgttcaccgttagatatcaaagggaaaactgtccatatgcacagatgaaaacggtgtaaaaaagatagatacatc
agagcttttacgagtttttggtgcatttaaagctgttcaccatgaacagatcgacaatgtaacctcgagattaccgcctttgagtgagcGGACCAAAACGAAAAAAGGCCCCCC
TTTCGGGAGGCCTCTTTTCTGG

RBS32 (replacement of *kilA* coding sequence)

tcacacaggaaag

*repL*$^{AT1}$, mutation in AT1-region represented by uppercase red letters

atgctggctaaagtcactttcctgagctgtataacgatgagcgattttactttttctggctatgaattggcctgctttgtaacacactccggtctatcccgtagcgccgggcata
tcctgtcgcaatgtgcaaatctcgcggcaacaaccagtgaatacttcattcacaagcctcaccgcctgatcgcggcagaaactggttatagccaatcaaccgtcgttcg
tgcattccgtgaagctgtaaacaaaggaattctgtctgtagagattgttatcggcgatcaccgtgaacgtcgcgctaacctgtaccggtttacaccatccttttttggccttcg
cacaacaagccaaaaatgcgctgatagaaagcaaattaaagatctcttcagcggcaaccaaggttaaagctgttctcgcGaagacGCtggcGCtGttCaaCttt
ttatccacacccccatgtcaaaatgataccccctcccccctgtcaggatgacgtggcaataaagaataagaagtcacaagttaaaaaaacaaaaagatcagtttccgg
cggtgccggaacaaccagcctcaaaaaattgacttcatggatcgctaaggcaaaagcaaaggctgacaatctgcggttatccaaaaaacgcactcaaaaacatga
gttcaagcagaaagtagaggcggctgcgcggaaatatgcttacctgaagaacaagcgttcgcctgatattggcgggatatcaaacttcgataacctaccgcattgcat
gacggtaaacgaagctcttaatgcggttttagccaaaaataaagataacgaacaatggggtataccggcaggattcagagggtaa

*repL*$^{D1}$, mutation in DnaA binding site 1 represented by uppercase red letters

atgctggctaaagtcactttcctgagctgtataacgatgagcgattttactttttctggctatgaattggcctgctttgtaacacactccggtctatcccgtagcgccgggcata
tcctgtcgcaatgtgcaaatctcgcggcaacaaccagtgaatacttcattcacaagcctcaccgcctgatcgcggcagaaactggttatagccaatcaaccgtcgttcg
tgcattccgtgaagctgtaaacaaaggaattctgtctgtagagattgttatcggcgatcaccgtgaacgtcgcgctaacctgtaccggtttacaccatccttttttggccttcg
cacaacaagccaaaaatgcgctgatagaaagcaaattaaagatctcttcagcggcaaccaaggttaaagctgttctcgctaagacattggctttatttaattttCtGtcc
acGcccccatgtcaaaatgataccccctcccccctgtcaggatgacgtggcaataaagaataagaagtcacaagttaaaaaaacaaaaagatcagtttccggcggt
gccggaacaaccagcctcaaaaaattgacttcatggatcgctaaggcaaaagcaaaggctgacaatctgcggttatccaaaaaacgcactcaaaaacatgagttc
aagcagaaagtagaggcggctgcgcggaaatatgcttacctgaagaacaagcgttcgcctgatattggcgggatatcaaacttcgataacctaccgcattgcatgac
ggtaaacgaagctcttaatgcggttttagccaaaaataaagataacgaacaatggggtataccggcaggattcagagggtaa

*repL*$^{AT2}$, mutation in AT2-region represented by uppercase red letters

atgctggctaaagtcactttcctgagctgtataacgatgagcgattttactttttctggctatgaattggcctgctttgtaacacactccggtctatcccgtagcgccgggcata
tcctgtcgcaatgtgcaaatctcgcggcaacaaccagtgaatacttcattcacaagcctcaccgcctgatcgcggcagaaactggttatagccaatcaaccgtcgttcg
tgcattccgtgaagctgtaaacaaaggaattctgtctgtagagattgttatcggcgatcaccgtgaacgtcgcgctaacctgtaccggtttacaccatccttttttggccttcg
cacaacaagccaaaaatgcgctgatagaaagcaaattaaagatctcttcagcggcaaccaaggttaaagctgttctcgctaagacattggctttatttaatttttttatcca
cacccccatgtcaaaatgataccccctcccccctgtcaggatgacgtggcGatCaagaaCaagaagAGCcaGgtGaaGaaGacGaaGCgTAGCgtttc
cggcggtgccggaacaaccagcctcaaaaaattgacttcatggatcgctaaggcaaaagcaaaggctgacaatctgcggttatccaaaaaacgcactcaaaaac
atgagttcaagcagaaagtagaggcggctgcgcggaaatatgcttacctgaagaacaagcgttcgcctgatattggcgggatatcaaacttcgataacctaccgcatt
gcatgacggtaaacgaagctcttaatgcggttttagccaaaaataaagataacgaacaatggggtataccggcaggattcagagggtaa

*repL*$^{D2}$, mutation in DnaA binding site 2 represented by uppercase red letters

atgctggctaaagtcactttcctgagctgtataacgatgagcgattttactttttctggctatgaattggcctgctttgtaacacactccggtctatcccgtagcgccgggcata
tcctgtcgcaatgtgcaaatctcgcggcaacaaccagtgaatacttcattcacaagcctcaccgcctgatcgcggcagaaactggttatagccaatcaaccgtcgttcg
tgcattccgtgaagctgtaaacaaaggaattctgtctgtagagattgttatcggcgatcaccgtgaacgtcgcgctaacctgtaccggtttacaccatccttttttggccttcg
cacaacaagccaaaaatgcgctgatagaaagcaaattaaagatctcttcagcggcaaccaaggttaaagctgttctcgctaagacattggctttatttaatttttttatcca
cacccccatgtcaaaatgataccccctcccccctgtcaggatgacgtggcaataaagaataagaagtcacaagttaaaaaaacaaaaagatcagtttccggcggtgc
cggaacaaccagcctcaaaaaattgacttcatggatcgctaaggcaaaagcaaaggctgacaatctgcggCtGtccaaGaaacgcactcaaaaacatgagttc
aagcagaaagtagaggcggctgcgcggaaatatgcttacctgaagaacaagcgttcgcctgatattggcgggatatcaaacttcgataacctaccgcattgcatgac
ggtaaacgaagctcttaatgcggttttagccaaaaataaagataacgaacaatggggtataccggcaggattcagagggtaa

nc-*repL* sequence (full length)

taaagcgattttactttttctggctatgaattggcctgctttgtaacacactccggtctatcccgtagcgccgggcatatcctgtcgcaatgtgcaaatctcgcggcaacaaccagtgaatacttcat
tcacaagcctcaccgcctgatcgcggcagaaactggttatagccaatcaaccgtcgttcgtgcattccgtgaagctgtaaacaaaggtattctgtctgtagagattgttatcggcgatcaccgtg
aacgtcgcgctaacctgtaccggtttacaccatccttttttggccttcgcacaacaagccaaaaatgcgctgatagaaagcaaattaaagatttcgtcagcggcaaccaaggttaaagctgttct
cgctaagacattggctttatttaatttttttatccacacccccatgtcaaaatgataccccctcccccctgtcaggatgacgtggcaataaagaataagaagtcacaagttaaaaaaacaaaaag
atcagtttccggcggtgccggaacaaccagcctcaaaaaattgacttcatggatcgctaaggcaaaagcaaaggctgacaatctgcggttatccaaaaaacgcactcaaaaacatgagtt
caagcagaaagtagaggcggctgcgcggaaatatgcttacctgaaaaacaagcgttcgcctgatattggcgggatatcaaacttcgataacctaccgcattgcatgacggtaaacgaagc
tcttaatgcggttttagccaaaaataaagataacgaacaatggggtataccggcaggattcagagggtaa

T1 nc-*repL* sequence

taaagcgattttactttttctggctatgaattggcctgctttgtaacacactccggtctatcccgtagcgccgggcatatcctgtcgcaatgtgcaaatctcgcggcaacaac
cagtgaatacttcattcacaagcctcaccgcctgatcgcggcagaaactggttatagccaatcaaccgtcgttcgtgcattccgtgaagctgtaaacaaaggtattctgtc
tgtagagattgttatcggcgatcaccgtgaacgtcgcgctaacctg

T2 nc-*repL* sequence

taccggtttacaccatccttttttggccttcgcacaacaagccaaaaatgcgctgatagaaagcaaattaaagatttcGtcagcggcaaccaaggttaaagctgttctcg
ctaagacattggctttatttaatttttttatccacacccccatgtcaaaatgataccccctcccccctgtcaggatgacgtggcaataaagaataagaagtcacaagttaaaa
aaacaaaaagatcagtttccggcggtgccggaacaaccagcctcaaaaaa

T3 nc-*repL* sequence

ttaccctctgaatcctgccggtataccccattgttcgttatctttatttttggctaaaaccgcattaagagcttcgtttaccgtcatgcaatgcggtaggttatcgaagtttgatatc
ccgccaatatcaggcgaacgcttgttTttcaggtaagcatatttccgcgcagccgcctctactttctgcttgaactcatgtttttgagtgcgtttttggataaccgcagattgtc
agcctttgcttttgccttagcgatccatgaagtcaa

T4 nc-*repL* sequence

gctaagacattggctttatttaatttttatccacacccccatgtcaaaatgatacccctccccctgtcaggatgacgtggcaataaagaataagaagtcacaagttaaa
aaaacaaaaagatcagtttccggcggtgccggaacaaccagcctcaaaaaa

T5 nc-*repL* sequence

ccccctccccctgtcaggatgacgtggcaataaagaataagaagtcacaagttaaaaaaacaaaaagatcagtttccggcggtgccgg

RK2 plasmid backbone, with npt gene in blue

gtcgtgactgggaaaaccctggcgactagtcttggactcctgttgatagatccagtaatgacctcagaactccatctggatttgttcagaacgctcggttgccgccgggcgttttttattggtgaga
atccaggggtccccaataattacgatttaaatttgtgtctcaaaatctctgatgttacattgcacaagataaaaatatatcatcatgaacaataaaactgtctgcttacataaacagtaatacaag
gggtgttatgagccatattcagcgtgaaacgagctgtagccgtccgcgtctgaacagcaacatggatgcggatctgtatggctataaatgggcgcgtgataacgtgggtcagagcggcgcg
accatttatcgtctgtatggcaaaccggatgcgccggaactgtttctgaaacatggcaaaggcagcgtggcgaacgatgtgaccgatgaaatggtgcgtctgaactggctgaccgaatttat
gccgctgccgaccattaaacattttattcgcaccccggatgatgcgtggctgctgaccaccgcgattccgggcaaaaccgcgtttcaggtgctggaagaatatccggatagcggcgaaaac
attgtggatgcgctggccgtgtttctgcgtcgtctgcatagcattccggtgtgcaactgcccgtttaacagcgatcgtgtgtttcgtctggcccaggcgcagagccgtatgaacaacggcctggtg
gatgcgagcgattttgatgatgaacgtaacggctggccggtggaacaggtgtggaaagaaatgcataaactgctgccgtttagcccggatagcgtggtgacccacggcgattttagcctgg
ataacctgatttcgatgaaggcaaactgattggctgcattgatgtgggccgtgtgggcattgcggatcgttatcaggatctggccattctgtggaactgcctgggcgaatttagcccgagcctgc
aaaaacgtctgtttcagaaatatggcattgataatccggatatgaacaaactgcaatttcatctgatgctggatgaatttttctaataattaattggaccgcggtccgcgcgttgtccttttccgctgc
ataaccctgcttcggggtcattatagcgatttttcggtatatccatcctttttcgcacgatatacaggattttgccaaagggttcgtgtagactttccttggtgtatccaacggcgtcagccgggcag
gataggtgaagtaggcccacccgcgagcgggtgttccttcttcactgtcccttattcgcacctggcggtgctcaacgggaatcctgctctgcgaggctggccgtaggccggccgcgatgcag
gtggctgctgaacccccagccggaactgaccccacaaggccctagcgtttgcaatgcaccaggtcatcattgacccaggcgtgttccaccaggccgctgcctcgcaactcttcgcaggctt
cgccgacctgctcgcgccacttcttcacgcgggtggaatccgatccgcacatgaggcggaaggtttccagcttgagcgggtacggctcccggtgcgagctgaaatagtcgaacatccgtcg
ggccgtcggcgacagcttgcggtacttctcccatatgaatttcgtgtagtggtcgccagcaaacagcacgacgatttcctcgtcgatcaggacctggcaacgggacgttttcttgccacggtcc
aggacgcggaagcggtgcagcagcgacaccgattccaggtgcccaacgcggtcggacgtgaagcccatcgccgtcgcctgtaggcgcgacaggcattcctcggccttcgtgtaatacc
ggccattgatcgaccagcccaggtcctggcaaagctcgtagaacgtgaaggtgatcggctcgccgataggggtgcgcttcgcgtactccaacacctgctgccacaccagttcgtcatcgtc
ggcccgcagctcgacgccggtgtaggtgatcttcacgtccttgttgacgtggaaaatgaccttgttttgcagcgcctcgcgcgggattttcttgttgcgcgtggtgaacagggcagagcgggcc
gtgtcgtttggcatcgctcgcatcgtgtccggccacggcgcaatatcgaacaaggaaagctgcatttccttgatctgctgcttcgtgtgtttcagcaacgcggcctgcttggcttcgctgacctgttt
tgccaggtcctcgccggcggtttttcgcttcttggtcgtcatagttcctcgcgtgtcgatggtcatcgacttcgccaaacctgccgcctcctgttcgagacgacgcgaacgctccacggcggccg
atggcgcgggcagggcagggggagccagttgcacgctgtcgcgctcgatcttggccgtagcttgctggactatcgagccgacggactggaaggtttcgcggggcgcacgcatgacggtg
cggcttgcgatggtttcggcatcctcggcggaaaaccccgcgtcgatcagttcttgcctgtatgccttccggtcaaacgtccgattcattcaccctccttgcgggattgccccggaattaattcccc
ggatcgatccgtcgatcttgatcccctgcgccatcagatccttggcggcaagaaagccatccagtttactttgcagggcttcccaaccttaccagagggcgccccagctggcaattccggttc
gcttgctgtccataaaaccgcccagtctagctatcgccatgtaagcccactgcaagctacctgctttctctttgcgcttgcgttttcccttgtccagatagcccagtagctgacattcatccggggtc
agcaccgtttctgcggactggctttctacgtggctgccatttttggggtgaggccgttcgcggccgaggggcgcagcccctggggggatgggaggcccgcgttagcgggccgggagggttc
gagaagggggggcaccccccttcggcgtgcgcggtcacgcgcacagggcgcagccctggttaaaaacaaggtttataaatattggtttaaaagcaggttaaaagacaggttagcggtgg
ccgaaaaacgggcggaaacccttgcaaatgctggattttctgcctgtggacagcccctcaaatgtcaataggtgcgcccctcatctgtcagcactctgcccctcaagtgtcaaggatcgcgc
ccctcatctgtcagtagtcgcgcccctcaagtgtcaataccgcagggcacttatccccaggcttgtccacatcatctgtgggaaactcgcgtaaaatcaggcgttttcgccgatttgcgaggctg
gccagctccacgtcgccggccgaaatcgagcctgcccctcatctgtcaacgccgcgccgggtgagtcggcccctcaagtgtcaacgtccgcccctcatctgtcagtgagggccaagttttcc
gcgaggtatccacaacgccggcggccctacatggctctgctgtagtgagtgggttgcgctccggcagcggtcctgatccccgcagaaaaaaaggatctcaagaagatcctttgatcttttct
acggcgcgcccagctgtctagggcggcggctcggtaccaaattccagaaaagaggcctcccgaaagggggcctttttcgttttggtccctgcagcggccgctactagtatataaacgca
gaaaggcccacccgaaggtgagccagtgtgactctagtagagagcgttcaccgacaaacaacagataaaacgaaaggcccagtctttcgactgagcctttcgttttatttgatgcctggctc
tagtatta

The J23115.RBS30.*rfp* construct, with J23115 in lower cases alphabets, RBS30 in orange, *rfp* in red

tttatagctagctcagcccttggtacaatgctagctCTAGAGATTAAAGAGGAGAAATACTAGATGGTGAGCAAGGGCGAGGAGGAT
AACATGGCCATCATCAAGGAGTTCATGCGCTTCAAGGTGCACATGGAGGGCTCCGTGAACGGCCACGAGTTCGA
GATCGAGGGCGAGGGCGAGGGCCGCCCCTACGAGGGCACCCAGACCGCCAAGCTGAAGGTGACCAAGGGTGG
CCCCCTGCCCTTCGCCTGGGACATCCTGTCCCCTCAGTTCATGTACGGCTCCAAGGCCTACGTGAAGCACCCCG
CCGACATCCCCGACTACTTGAAGCTGTCCTTCCCCGAGGGCTTCAAGTGGGAGCGCGTGATGAACTTCGAGGAC
GGCGGCGTGGTGACCGTGACCCAGGACTCCTCCTTGCAGGACGGCGAGTTCATCTACAAGGTGAAGCTGCGCG
GCACCAACTTCCCCTCCGACGGCCCCGTAATGCAGAAGAAGACCATGGGCTGGGAGGCCTCCTCCGAGCGGAT
GTACCCCGAGGACGGCGCCCTGAAGGGCGAGATCAAGCAGAGGCTGAAGCTGAAGGACGGCGGCCACTACGA
CGCTGAGGTCAAGACCACCTACAAGGCCAAGAAGCCCGTGCAGCTGCCCGGCGCCTACAACGTCAACATCAAGT
TGGACATCACCTCCCACAACGAGGACTACACCATCGTGGAACAGTACGAACGCGCCGAGGGCCGCCACTCCAC
CGGCGGCATGGACGAGCTGTACAAGTAA

AT2 region with SL1 mutation (base substitution in uppercase letter(s))

Gataaagaataagaagtcacaagttaaaaaaacaaaaagatca

AT2 region with SL2 mutation (base substitution in uppercase letter(s))

aatCaagaataagaagtcacaagttaaaaaaacaaaaagatca

| |
|---|
| AT2 region with SL3 mutation (base substitution in uppercase letter(s))<br>aataaagaaCaagaagtcacaagttaaaaaaacaaaaagatca |
| AT2 region with SL4 mutation (base substitution in uppercase letter(s))<br>aataaagaataagaagAGCcaagttaaaaaaacaaaaagatca |
| AT2 region with SL5 mutation (base substitution in uppercase letter(s))<br>aataaagaataagaagtcacaGgttaaaaaaacaaaaagatca |
| AT2 region with SL6 mutation (base substitution in uppercase letter(s))<br>aataaagaataagaagtcacaagtGaaaaaaacaaaaagatca |
| AT2 region with SL7 mutation (base substitution in uppercase letter(s))<br>aataaagaataagaagtcacaagttaaGaaaacaaaaagatca |
| AT2 region with SL8 mutation (base substitution in uppercase letter(s))<br>aataaagaataagaagtcacaagttaaaaaGacaaaaagatca |
| AT2 region with SL9 mutation (base substitution in uppercase letter(s))<br>aataaagaataagaagtcacaagttaaaaaaacGaaaagatca |
| AT2 region with SL10 mutation (base substitution in uppercase letter(s))<br>aataaagaataagaagtcacaagttaaaaaaacaaaGCgatca |
| AT2 region with SL11 mutation (base substitution in uppercase letter(s))<br>aataaagaataagaagtcacaagttaaaaaaacaaaaagTAGC |
| AT2 region with SL2+5 mutation (base substitution in uppercase letter(s))<br>aatCaagaataagaagtcacaGgttaaaaaaacaaaaagatca |
| AT2 region with SL2+6 mutation (base substitution in uppercase letter(s))<br>aatCaagaataagaagtcacaagtGaaaaaaacaaaaagatca |
| AT2 region with SL2+7 mutation (base substitution in uppercase letter(s))<br>aatCaagaataagaagtcacaagttaaGaaaacaaaaagatca |
| AT2 region with SL1+2+3 mutation (base substitution in uppercase letter(s))<br>GatCaagaaCaagaagtcacaagttaaaaaaacaaaaagatca |
| AT2 region with SL4+5+6 mutation (base substitution in uppercase letter(s))<br>aataaagaataagaagAGCcaGgtGaaaaaaacaaaaagatca |
| AT2 region with SL7+8+9 mutation (base substitution in uppercase letter(s))<br>aataaagaataagaagtcacaagttaaGaaGacGaaaagatca |
| AT2 region with SL1+2+3+4 mutation (base substitution in uppercase letter(s))<br>GatCaagaaCaagaagAGCcaagttaaaaaaacaaaaagatca |
| AT2 region with SL1+2+7+8 mutation (base substitution in uppercase letter(s))<br>GatCaagaataagaagtcacaagttaaGaaGacaaaaagatca |

AT2 region with SL8+9+10+11 mutation (base substitution in uppercase letter(s))

aataaagaataagaagtcacaagttaaaaaGacGaaGCgTAGC

$P_{arsR}$-ABS62-32malE-linte-TEV-kilA-$repL^{AT2}$-B15@221 (Aresenic biosensor plasmid 1, *repL*$^{AT2}$ allele highlighted in yellow, mutated AT2 region highlighted in cyan)

TTAATTAAAGCGGATAACAATTTCACACAGGAGGCCGCCTAGGCCGCGGCCGCGCGAATTCGCGGCCGCTTCTA
GAGCCAACTCAAAATTCACACCTATTACCTTCCTCTGCACTTACACATTCGTTAAGTCATATATGTTTTTGACTTAT
CCGCTTCGAAGAGAGACACTACCTGCAAACTTACACATAAGTCATATATGTTTTTGACTTATCCGCTTCTACTAGA
GTCACACAGGAAAGTACTAGATGATCGAAGAAGGTAAACTGGTAATCTGGATTAACGGCGATAAAGGCTATAACG
GTCTCGCTGAAGTCGGTAAGAAATTCGAGAAAGATACCGGAATTAAAGTCACCGTTGAGCATCCGGATAAACTGG
AAGAGAAATTCCCACAGGTTGCGGCAACTGGCGATGGCCCTGACATTATCTTCTGGGCACACGACCGCTTTGGT
GGCTACGCTCAATCTGGCCTGTTGGCTGAAATCACCCCGGACAAAGCGTTCCAGGACAAGCTGTATCCGTTTAC
CTGGGATGCCGTACGTTACAACGGCAAGCTGATTGCTTACCCGATCGCTGTTGAAGCGTTATCGCTGATTTATAA
CAAAGATCTGCTGCCGAACCCGCCAAAAACCTGGGAAGAGATCCCGGCGCTGGATAAAGAACTGAAAGCGAAAG
GTAAGAGCGCGCTGATGTTCAACCTGCAAGAACCGTACTTCACCTGGCCGCTGATTGCTGCTGACGGGGGTTAT
GCGTTCAAGTATGAAAACGGCAAGTACGACATTAAAGACGTGGGCGTGGATAACGCTGGCGCGAAAGCGGGTCT
GACCTTCCTGGTTGACCTGATTAAAAACAAACACATGAATGCAGACACCGATTACTCCATCGCAGAAGCTGCCTTT
AATAAAGGCGAAACAGCGATGACCATCAACGGCCCGTGGGCATGGTCCAACATCGACACCAGCAAAGTGAATTA
TGGTGTAACGGTACTGCCGACCTTCAAGGGTCAACCATCCAAACCGTTCGTTGGCGTGCTGAGCGCAGGTATTA
ACGCCGCCAGTCCGAACAAAGAGCTGGCGAAAGAGTTCCTCGAAAACTATCTGCTGACTGATGAAGGTCTGGAA
GCGGTTAATAAAGACAAACCGCTGGGTGCCGTAGCGCTGAAGTCTTACGAGGAAGAGTTGGCGAAAGATCCACG
TATTGCCGCCACCATGGAAAACGCCCAGAAAGGTGAAATCATGCCGAACATCCCGCAGATGTCCGCTTTCTGGTA
TGCCGTGCGTACTGCGGTGATCAACGCCGCCAGCGGTCGTCAGACTGTCGATGAAGCCCTGAAAGACGCGCAG
ACTCGTATCACCAAGGGCGGCTCCGGCGGCTCCGGCGGCACCGGCGGCTCCGGCGGCTCCGAAAACCTGTATT
TTCAGGGTGGCGGCGAAAGCCTGTTCAAAGGTCCGCGTGACTACAACCCGATTAGCTCGACGATCTGCCACCTG
ACGAACGAAAGCGACGGCCACACCACGAGCCTGTATGGCATCGGTTTTGGCCCGTTCATTATCACGAACAAACA
CCTGTTTCGTCGCAACAATGGTACCCTGCTGGTGCAGTCTCTGCATGGCGTGTTTAAAGTTAAAAATACCACGAC
CCTGCAACAACACCTGATCGATGGTCGTGACATGATTATCATTCGCATGCCGAAAGATTTTCCGCCGTTCCCGCA
GAAACTGAAATTCCGTGAACCGCAACGTGAAGAACGCATTTGCCTGGTCACGACCAACTTTCAGACCAAATCAAT
GAGCTCTATGGTTAGCGATACGTCTTGTACCTTTCCGAGTTCCGACGGCATCTTCTGGAAACATTGGATTCAGAC
CAAAGATGGTCAATGCGGCAGTCCGCTGGTTTCCACCCGTGACGGTTTCATCGTCGGCATTCACTCAGCGTCGA
ACTTTACGAATACCAACAATTACTTCACGTCCGTTCCGAAAAACTTTATGGAACTGCTGACCAATCAGGAAGCGCA
GCAATGGGTGTCAGGTTGGCGCCTGAATGCCGATTCGGTTCTGTGGGGCGGTCACAAAGTCTTTATGGTGAAAC
CGGAAGAACCGTTCCAGCCGGTCAAAGAAGCAACCCAACTGATGAATTAATAATACTAGAGgtacctgtcgcggcaacgcg
ctaacagacgtagtaagaaccaccagcattgtaatgctggctaaagtcactttcctgagctgtataacgatgagcgattttactttttctggctatgaattggcctgctttgta
acacactccggtctatcccgtagcgccgggcatatcctgtcgcaatgtgcaaatctcgcggcaacaaccagtgaatacttcattcacaagcctcaccgcctgatcgcg
gcagaaactggttatagccaatcaaccgtcgttcgtgcattccgtgaagctgtaaacaaaggaatcctgtctgtagagattgttatcggcgatcaccgtgaacgtcgcgc
taacctgtaccggtttacaccatcctttttggccttcgcacaacaagccaaaaatgcgctgatagaaagcaaattaaagatctcttcagcggcaaccaaggttaaagct
gttctcgctaagacattggctttatttaatttttttatccacacccccatgtcaaaatgataccccctccccctgtcaggatgacgtgGCGATCAAGAACAAGAAG
AGCCAGGTGAAGAAGACGAAGCGTAGCgtttccggcggtgccggaacaaccagcctcaaaaaattgacttcatggatcgctaaggcaaaagcaa
aggctgacaatctgcggttatccaaaaaacgcactcaaaaacatgagttcaagcagaaagtagaggcggctgcgcggaaatatgcttacctgaagaacaagcgtt
cgcctgatattggcgggatatcaaacttcgataacctaccgcattgcatgacggtaaacgaagctcttaatgcggttttagccaaaaataaagataacgaacaatggg
gtataccggcaggattcagagggtaataaTACTAGAGCCAGGCATCAAATAAAACGAAAGGCTCAGTCGAAAGACTGGGCCTT
TCGTTTTATCTGTTGTTTGTCGGTGAACGCTCTCTACTAGAGTCACACTGGCTCACCTTCGGGTGGGCCTTTCTG
CGTTTATATACTAGTAGCGGCCGCTGCAGGCATGCAAGCTTGCGGCCGCGTCGTGACTGGGAAAACCCTGGCGA
CTAGTCTTGGACTCCTGTTGATAGATCCAGTAATGACCTCAGAACTCCATCTGGATTTGTTCAGAACGCTCGGTTG
CCGCCGGGCGTTTTTTATTGGTGAGAATCCAGGGGTCCCCAATAATTACGATTTAAATTTGTGTCTCAAAATCTCT
GATGTTACATTGCACAAGATAAAAATATATCATCATGAACAATAAAACTGTCTGCTTACATAAACAGTAATACAAGG
GGTGTTATGAGCCATATTCAGCGTGAAACGAGCTGTAGCCGTCCGCGTCTGAACAGCAACATGGATGCGGATCT
GTATGGCTATAAATGGGCGCGTGATAACGTGGGTCAGAGCGGCGCGACCATTTATCGTCTGTATGGCAAACCGG
ATGCGCCGGAACTGTTTCTGAAACATGGCAAAGGCAGCGTGGCGAACGATGTGACCGATGAAATGGTGCGTCTG
AACTGGCTGACCGAATTTATGCCGCTGCCGACCATTAAACATTTTATTCGCACCCCGGATGATGCGTGGCTGCTG
ACCACCGCGATTCCGGGCAAAACCGCGTTTCAGGTGCTGGAAGAATATCCGGATAGCGGCGAAAACATTGTGGA
TGCGCTGGCCGTGTTTCTGCGTCGTCTGCATAGCATTCCGGTGTGCAACTGCCCGTTTAACAGCGATCGTGTGTT
TCGTCTGGCCCAGGCGCAGAGCCGTATGAACAACGGCCTGGTGGATGCGAGCGATTTTGATGATGAACGTAACG
GCTGGCCGGTGGAACAGGTGTGGAAAGAAATGCATAAACTGCTGCCGTTTAGCCCGGATAGCGTGGTGACCCAC
GGCGATTTTAGCCTGGATAACCTGATTTTCGATGAAGGCAAACTGATTGGCTGCATTGATGTGGGCCGTGTGGGC
ATTGCGGATCGTTATCAGGATCTGGCCATTCTGTGGAACTGCCTGGGCGAATTTAGCCCGAGCCTGCAAAAACGT
CTGTTTCAGAAATATGGCATTGATAATCCGGATATGAACAAACTGCAATTTCATCTGATGCTGGATGAATTTTTCTA
ATAATTAATTGGACCGCGGTCCGCGCGTTGTCCTTTTCCGCTGCATAACCCTGCTTCGGGGTCATTATAGCGATT
TTTTCGGTATATCCATCCTTTTTCGCACGATATACAGGATTTTGCCAAAGGGTTCGTGTAGACTTTCCTTGGTGTAT
CCAACGGCGTCAGCCGGGCAGGATAGGTGAAGTAGGCCCACCCGCGAGCGGGTGTTCCTTCTTCACTGTCCCT
TATTCGCACCTGGCGGTGCTCAACGGGAATCCTGCTCTGCGAGGCTGGCCGTAGGCCGGCCGCGATGCAGGTG
GCTGCTGAACCCCCAGCCGGAACTGACCCCACAAGGCCCTAGCGTTTGCAATGCACCAGGTCATCATTGACCCA
GGCGTGTTCCACCAGGCCGCTGCCTCGCAACTCTTCGCAGGCTTCGCCGACCTGCTCGCGCCACTTCTTCACGC
GGGTGGAATCCGATCCGCACATGAGGCGGAAGGTTTCCAGCTTGAGCGGGTACGGCTCCCGGTGCGAGCTGAA
ATAGTCGAACATCCGTCGGGCCGTCGGCGACAGCTTGCGGTACTTCTCCCATATGAATTTCGTGTAGTGGTCGC
CAGCAAACAGCACGACGATTTCCTCGTCGATCAGGACCTGGCAACGGGACGTTTTCTTGCCACGGTCCAGGACG
CGGAAGCGGTGCAGCAGCGACACCGATTCCAGGTGCCCAACGCGGTCGGACGTGAAGCCCATCGCCGTCGCCT

GTAGGCGCGACAGGCATTCCTCGGCCTTCGTGTAATACCGGCCATTGATCGACCAGCCCAGGTCCTGGCAAAGC
TCGTAGAACGTGAAGGTGATCGGCTCGCCGATAGGGGTGCGCTTCGCGTACTCCAACACCTGCTGCCACACCAG
TTCGTCATCGTCGGCCCGCAGCTCGACGCCGGTGTAGGTGATCTTCACGTCCTTGTTGACGTGGAAAATGACCT
TGTTTTGCAGCGCCTCGCGCGGGATTTTCTTGTTGCGCGTGGTGAACAGGGCAGAGCGGGCCGTGTCGTTTGG
CATCGCTCGCATCGTGTCCGGCCACGGCGCAATATCGAACAAGGAAAGCTGCATTTCCTTGATCTGCTGCTTCGT
GTGTTTCAGCAACGCGGCCTGCTTGGCTTCGCTGACCTGTTTTGCCAGGTCCTCGCCGGCGGTTTTTCGCTTCTT
GGTCGTCATAGTTCCTCGCGTGTCGATGGTCATCGACTTCGCCAAACCTGCCGCCTCCTGTTCGAGACGACGCG
AACGCTCCACGGCGGCCGATGGCGCGGGCAGGGCAGGGGGAGCCAGTTGCACGCTGTCGCGCTCGATCTTGG
CCGTAGCTTGCTGGACTATCGAGCCGACGGACTGGAAGGTTTCGCGGGGCGCACGCATGACGGTGCGGCTTGC
GATGGTTTCGGCATCCTCGGCGGAAAACCCCGCGTCGATCAGTTCTTGCCTGTATGCCTTCCGGTCAAACGTCC
GATTCATTCACCCTCCTTGCGGGATTGCCCCGGAATTAATTCCCCGGATCGATCCGTCGATCTTGATCCCCTGCG
CCATCAGATCCTTGGCGGCAAGAAAGCCATCCAGTTTACTTTGCAGGGCTTCCCAACCTTACCAGAGGGCGCCC
CAGCTGGCAATTCCGGTTCGCTTGCTGTCCATAAAACCGCCCAGTCTAGCTATCGCCATGTAAGCCCACTGCAAG
CTACCTGCTTTCTCTTTGCGCTTGCGTTTTCCCTTGTCCAGATAGCCCAGTAGCTGACATTCATCCGGGGTCAGC
ACCGTTTCTGCGGACTGGCTTTCTACGTGGCTGCCATTTTTGGGGTGAGGCCGTTCGCGGCCGAGGGGCGCAG
CCCCTGGGGGGATGGGAGGCCCGCGTTAGCGGGCCGGGAGGGTTCGAGAAGGGGGGGCACCCCCCTTCGGC
GTGCGCGGTCACGCGCACAGGGCGCAGCCCTGGTTAAAAACAAGGTTTATAAATATTGGTTTAAAAGCAGGTTAA
AAGACAGGTTAGCGGTGGCCGAAAAACGGGCGGAAACCCTTGCAAATGCTGGATTTTCTGCCTGTGGACAGCCC
CTCAAATGTCAATAGGTGCGCCCCTCATCTGTCAGCACTCTGCCCCTCAAGTGTCAAGGATCGCGCCCCTCATCT
GTCAGTAGTCGCGCCCCTCAAGTGTCAATACCGCAGGGCACTTATCCCCAGGCTTGTCCACATCATCTGTGGGA
AACTCGCGTAAAATCAGGCGTTTTCGCCGATTTGCGAGGCTGGCCAGCTCCACGTCGCCGGCCGAAATCGAGCC
TGCCCCTCATCTGTCAACGCCGCGCCGGGTGAGTCGGCCCCTCAAGTGTCAACGTCCGCCCCTCATCTGTCAGT
GAGGGCCAAGTTTTCCGCGAGGTATCCACAACGCCGGCGGCCCTACATGGCTCTGCTGTAGTGAGTGGGTTGC
GCTCCGGCAGCGGTCCTGATCCCCCGCAGAAAAAAAGGATCTCAAGAAGATCCTTTGATCTTTTCTACGGCGCG
CCCAGCTGTCTAGGGCGGCGGATTTGTCCTACTCAGGAGAGCGTTCACCGACAAACAACAGATAAAACGAAAGG
CCCAGTCTTTCGACTGAGCCTTTCGTTTTATTTGATGCCT

J109-30ParsR-Amp30E-gfp-linte-AAV -B15-nc-*repL*$^{WT}$-B15@4A3 (arsenic biosensor plasmid 2, nc-repL sequence highlighted in yellow, WT AT2 region highlighted in cyan)

GTCCGGCAAAAAAACGGGCAAGGTGTCACCACCCTGCCCTTTTTCTTTAAAACCGAAAAGATTACTTCGCGTTAT
GCAGGCTTCCTCGCTCACTGACTCGCTGCGCTCGGTCGTTCGGCTGCGGCGAGCGGTATCAGCTCACTCAAAG
GCGGTAATCTCGAGGTTACATTGTCGATCTGTTCATGGTGAACAGCTTTAAATGCACCAAAAACTCGTAAAAGCTC
TGATGTATCTATCTTTTTTACACCGTTTTCATCTGTGCATATGGACAGTTTTCCCTTTGATATCTAACGGTGAACAG
TTGTTCTACTTTTGTTTGTTAGTCTTGATGCTTCACTGATAGATACAAGAGCCATAAGAACCTCAGATCCTTCCGTA
TTTAGCCAGTATGTTCTCTAGTGTGGTTCGTTGTTTTTGCGTGAGCCATGAGAACGAACCATTGAGATCATGCTTA
CTTTGCATGTCACTCAAAAATTTTGCCTCAAAACTGGTGAGCTGAATTTTTGCAGTTAAAGCATCGTGTAGTGTTTT
TCTTAGTCCGTTACGTAGGTAGGAATCTGATGTAATGGTTGTTGGTATTTTGTCACCATTCATTTTTATCTGGTTGT
TCTCAAGTTCGGTTACGAGATCCATTTGTCTATCTAGTTCAACTTGGAAAATCAACGTATCAGTCGGGCGGCCTCG
CTTATCAACCACCAATTTCATATTGCTGTAAGTGTTTAAATCTTTACTTATTGGTTTCAAAACCCATTGGTTAAGCCT
TTTAAACTCATGGTAGTTATTTTCAAGCATTAACATGAACTTAAATTCATCAAGGCTAATCTCTATATTTGCCTTGTG
AGTTTTCTTTTGTGTTAGTTCTTTTAATAACCACTCATAAATCCTCATAGAGTATTTGTTTTCAAAAGACTTAACATG
TTCCAGATTATATTTTATGAATTTTTTTAACTGGAAAAGATAAGGCAATATCTCTTCACTAAAAACTAATTCTAATTTT
TCGCTTGAGAACTTGGCATAGTTTGTCCACTGGAAAATCTCAAAGCCTTTAACCAAAGGATTCCTGATTTCCACAG
TTCTCGTCATCAGCTCTCTGGTTGCTTTAGCTAATACACCATAAGCATTTTCCCTACTGATGTTCATCATCTGAGCG
TATTGGTTATAAGTGAACGATACCGTCCGTTCTTTCCTTGTAGGGTTTTCAATCGTGGGGTTGAGTAGTGCCACAC
AGCATAAAATTAGCTTGGTTTCATGCTCCGTTAAGTCATAGCGACTAATCGCTAGTTCATTTGCTTTGAAAACAACT
AATTCAGACATACATCTCAATTGGTCTAGGTGATTTTAATCACTATACCAATTGAGATGGGCTAGTCAATGATAATT
ACATGTCCTTTTCCTTTGAGTTGTGGGTATCTGTAAATTCTGCTAGACCTTTGCTGGAAAACTTGTAAATTCTGCTA
GACCCTCTGTAAATTCCGCTAGACCTTTGTGTGTTTTTTTTGTTTATATTCAAGTGGTTATAATTTATAGAATAAAGA
AAGAATAAAAAAAGATAAAAAGAATAGATCCCAGCCCTGTGTATAACTCACTACTTTAGTCAGTTCCGCAGTATTA
CAAAAGGATGTCGCAAACGCTGTTTGCTCCTCTACAAAACAGACCTTAAAACCCTAAAGGCTTAAGTAGCACCCT
CGCAAGCTCGGGCAAATCGCTGAATATTCCTTTTGTCTCCGACCATCAGGCACCTGAGTCGCTGTCTTTTTCGTG
ACATTCAGTTCGCTGCGCTCACGGCTCTGGCAGTGAATGGGGGTAAATGGCACTACAGGCGCCTTTTATGGATT
CATGCAAGGAAACTACCCATAATACAAGAAAAGCCCGTCACGGGCTTCTCAGGGCGTTTTATGGCGGGTCTGCTA
TGTGGTGCTATCTGACTTTTTGCTGTTCAGCAGTTCCTGCCCTCTGATTTTCCAGTCTGACCACTTCGGATTATCC
CGTGACAGGTCATTCAGACTGGCTAATGCACCCAGTAAGGCAGCGGTATCATCAACAGGCTTACCCGTCTTACTG
TCCCTAGTGCTTGGATTCTCACCAATAAAAAACGCCCGGCGGCAACCGAGCGTTCTGAACAAATCCAGATGGAGT
TCTGAGGTCATTACTGGATCTATCAACAGGAGTCCAAGCGAGCTCGTAAACTTGGTCTGACAGTTACCAATGCTT
AATCAGTGAGGCACCTATCTCAGCGATCTGTCTATTTCGTTCATCCATAGTTGCCTGACTCCCCGTCGTGTAGATA
ACTACGATACGGGAGGGCTTACCATCTGGCCCCAGTGCTGCAATGATACCGCGAGACCCACGCTCACCGGCTCC
AGATTTATCAGCAATAAACCAGCCAGCCGGAAGGGCCGAGCGCAGAAGTGGTCCTGCAACTTTATCCGCCTCCA
TCCAGTCTATTAATTGTTGCCGGGAAGCTAGAGTAAGTAGTTCGCCAGTTAATAGTTTGCGCAACGTTGTTGCCAT
TGCTACAGGCATCGTGGTGTCACGCTCGTCGTTTGGTATGGCTTCATTCAGCTCCGGTTCCCAACGATCAAGGC
GAGTTACATGATCCCCCATGTTGTGCAAAAAAGCGGTTAGCTCCTTCGGTCCTCCGATCGTTGTCAGAAGTAAGT
TGGCCGCAGTGTTATCACTCATGGTTATGGCAGCACTGCATAATTCTCTTACTGTCATGCCATCCGTAAGATGCTT
TTCTGTGACTGGTGAGTACTCAACCAAGTCATTCTGAGAATAGTGTATGCGGCGACCGAGTTGCTCTTGCCCGGC
GTCAATACGGGATAATACCGCGCCACATAGCAGAACTTTAAAAGTGCTCATCATTGGAAAACGTTCTTCGGGGCG
AAAACTCTCAAGGATCTTACCGCTGTTGAGATCCAGTTCGATGTAACCCACTCGTGCACCCAACTGATCTTCAGC
ATCTTTTACTTTCACCAGCGTTTCTGGGTGAGCAAAAACAGGAAGGCAAAATGCCGCAAAAAAGGGAATAAGGGC

GACACGGAAATGTTGAATACTCATACTCTTCCTTTTTCAATATTATTGAAGCATTTATCAGGGTTATTGTCTCATGA
GCGGATACATATTTGAATGTATTTAGAAAAATAAACAAATAGGGGTTCCGCGCACATTTCCCATGGTGCCACCTGA
CGTCTAAGAAACCATTATTATCATGACATTAACCTATAAAAATAGGCGTATCACGAGGCAGAATTTCAGATAAAAAA
AATCCTTAGCTTTCGCTAAGGATGATTTCTGGAATTCGCGGCCGCTTCTAGAGTTTACAGCTAGCTCAGTCCTAG
GGACTGTGCTAGCTACTAGAGATTAAAGAGGAGAAATACTAGATGTCATTTCTGTTACCCATCCAATTGTTCAAAA
TTCTTGCTGATGAAACCCGTCTGGGCATCGTTTTACTGCTCAGCGAACTGGGAGAGTTATGCGTCTGCGATCTCT
GCACTGCTCTCGACCAGTCGCAGCCCAAGATCTCCCGCCACCTGGCATTGCTGCGTGAAAGCGGGCTATTGCTG
GACCGCAAGCAAGGTAAGTGGGTTCATTACCGCTTATCACCGCATATTCCAGCATGGGCGGCGAAAATTATTGAT
GAGGCCTGGCGATGTGAACAGGAAAAGGTTCAGGCGATTGTCCGCAACCTGGCTCGACAAAACTGTTCCGGGG
ACAGTAAGAACATTTGCAGTTAAAAATTTAGCTAAACACACATGAATTTTCAGATGTGTTTTATCCGGGTACTAGAG
CCAGGCATCAAATAAAACGAAAGGCTCAGTCGAAAGACTGGGCCTTTCGTTTTATCTGTTGTTTGTCGGTGAACG
CTCTCTACTAGAGTCACACTGGCTCACCTTCGGGTGGGCCTTTCTGCGTTTATATACTAGAGCCAACTCAAAATTC
ACACCTATTACCTTCCTCTGCACTTACACATTCGTTAAGTCATATATGTTTTTGACTTATCCGCTTCGAAGAGAGAC
ACTACCTGCAATACTAGAGATTAAAGAGGAGAAATACTAGATGAGTACAGGCATCGATAAGGACGTCCGAGAGTG
TTGGGGCGTAACTGCATTATCAGCGGGTCATCAAATTGCAATGAATAGCGCGTTTCTGGATATGGACTTGCTGTT
GTGCGGGGAAACCGGCACCGGCAAGGACACACTGGCCAACCGCATTCACGAGTTGTCCAGCAGGTCGGGACCC
TTTGTGGGCATGAACTGCGCCGCCATTCCCGAGTCGCTGGCAGAGAGCCAGTTATTCGGTGTGGTCAACGGTGC
ATTCACCGGCGTATGCCGGGCTCGCGAGGGCTACATAGAGGCCTCCAGTGGTGGCACCTTGTACCTGGATGAAA
TCGACAGCATGCCGTTGAGCCTGCAAGCCAAACTGCTGCGTGTGTTGGAGAGTCGAGGTATCGAGCGTCTGGG
CTCGACCGAATTTATCCCGGTGGATCTGCGGATCATTGCCTCGGCCCAGCGGCCACTGGATGAACTGGTGGAAC
AAGGACTTTTCCGTCGCGACCTGTTTTTTCGGCTCAACGTGCTGACGCTTCACTTGCCAGCCTTGCGCAAACGTC
GTGAACAGATCCTGCCATTGTTCGACCAGTTCACCCAGGGTATCGCTGCCGAGTTCGGACGTCCCGCTCCTGCG
CTGGACAGCGGGCGTGTGCAGCTGCTGCTCAGCCACGACTGGCCGGGCAACATCCGCGAATTGAAGTCTGCGG
CCAAGCGCTTCGTACTCGGCTTCCCCTTGCTGGGCGCCGACCCTGTGGAAGCGCTTGACCCTGCCACGGGGCT
GCGCACGCAAATGCGCATCATCGAGAAAATGCTCATCCAGGATGCCTTGAAGCGGCACAGGCACAATTTCGACG
CGGTGCTTCAGGAGTTGGAGTTGCCAAGACGCACCCTGTATCACCGCATGAAGGAACTGGGAGTTGCAGCGCC
GATCGCTGCGACGGCCGGGGTCTAATAAAACGCAGGTGTGGTTATGCTACTAGAGATTAAAGAGGAGAAATACT
AGATGAGTCTTGATGAAAGGTTTGAGGATGATCTGGACGAGGAGCGGGTTCCGAATCTGGGGATAGTTGCCGAA
AGTATTTCGCAACTGGGTATCGACGTGCTGCTATCGGGTGAGACCGGCACGGGCAAAGACACGATTGCCCGACG
GATTCATGAGATGTCAGGCCGCAAAGGGCGCCTGGTGGCGATGAATTGCGCGGCCATTCCGGAGTCCCTCGCC
GAGAGCGAGTTATTCGGCGTGGTCAGCGGTGCCTACACCGGCGCTGATCGCTCCAGAGTCGGTTATGTCGAAG
CGGCGCAGGGCGGCACGCTGTACCTGGATGAGATCGATAGCATGCCGCTGAGCCTGCAAGCCAAATTGCTGAG
GGTGCTGGAAACCCGAGCGCTTGAACGGCTGGGTTCGACGTCGACGATCAAGCTGGATATCTGCGTGATCGCCT
CCGCCCAATGCTCGCTGGACGACGCCGTCGAGCGGGGGCAGTTTCGTCGCGATCTGTATTTTCGCCTGAACGTC
CTGACACTCAAGCTTCCTCCGCTACGTAACCAGTCTGATCGCATAGTTCCCCTGTTCACACGTTTTACGGCCGCC
GCCGCGAGGGAGCTCGGTGTTCCCGTTCCCGATGTTTGCCCACTGCTGCACAAAGTGCTGCTGGGCCACGACT
GGCCCGGCAATATCCGTGAGCTCAAGGCGGCAGCCAAACGCCATGTGCTGGGTTTCCCCTTGCTGGGCGCCGA
GCCGCAGGGCGAAGAGCACTTGGCCTGTGGGCTCAAATCGCAATTGCGAGTGATCGAAAAAGCCCTGATTCAGG
AGTCGCTCAAGCGCCACGACAATTGTGTGGATTCGGTAAGCCTGGAACTGGACGTGCCACGCCGTACGCTCTAT
CGACGCATCAAAGAATTGCAGATCTAATAATACTAGAGCCAGGCATCAAATAAAACGAAAGGCTCAGTCGAAAGA
CTGGGCCTTTCGTTTTATCTGTTGTTTGTCGGTGAACGCTCTCTACTAGAGTCACACTGGCTCACCTTCGGGTGG
GCCTTTCTGCGTTTATATACTAGAGGCCGGATTATGTCCGCTGAGTGGGTCACGGTCCCGGATCAGTTCCCTTGC
GAAGCTGACCGATGTTTTTGTGCCAAAAGCTGTTGTGGCAAAAAACGGTTTGCGCAAAGTTTTGTATTACAAAGAA
TTTCACATTTTAAAATATCTTTATAAATCAATCAGTTATTTCTATTTTCAAGCTGGCACGGTTATTGCTATAGGGCTT
GTACTACTAGAGATTAAAGAGGAGAAATACTAGATGCGTAAAGGAGAAGAACTTTTCACTGGAGTTGTCCCAATTC
TTGTTGAATTAGATGGTGATGTTAATGGGCACAAATTTTCTGTCAGTGGAGAGGGTGAAGGTGATGCAACATACG
GAAAACTTACCCTTAAATTTATTTGCACTACTGGAAAACTACCTGTTCCATGGCCAACACTTGTCACTACTTTCGGT
TATGGTGTTCAATGCTTTGCGAGATACCCAGATCATATGAAACAGCATGACTTTTTCAAGAGTGCCATGCCCGAAG
GTTATGTACAGGAAAGAACTATATTTTTCAAAGATGACGGGAACTACAAGACACGTGCTGAAGTCAAGTTTGAAGG
TGATACCCTTGTTAATAGAATCGAGTTAAAAGGTATTGATTTTAAAGAAGATGGAAACATTCTTGGACACAAATTGG
AATACAACTATAACTCACACAATGTATACATCATGGCAGACAAACAAAAGAATGGAATCAAAGTTAACTTCAAAATT
AGACACAACATTGAAGATGGAAGCGTTCAACTAGCAGACCATTATCAACAAAATACTCCAATTGGCGATGGCCCT
GTCCTTTTACCAGACAACCATTACCTGTCCACACAATCTGCCCTTTCGAAAGATCCCAACGAAAAGAGAGACCAC
ATGGTCCTTCTTGAGTTTGTAACAGCTGCTGGGATTACACATGGCATGGATGAACTATACAAAGGCGGCTCCGGC
GGCTCCGGCGGCACCGGCGGCTCCGGCGGCTCCGAAAACCTGTATTTTCAGGGTGGCGCTGCAAACGACGAAA
ACTACGCTGCCGCAGTTTAATAATACTAGAGCCAGGCATCAAATAAAACGAAAGGCTCAGTCGAAAGACTGGGCC
TTTCGTTTTATCTGTTGTTTGTCGGTGAACGCTCTCTACTAGAGTCACACTGGCTCACCTTCGGGTGGGCCTTTCT
GCGTTTATATACTAGAGtaaagcgatttacttttctggctatgaattggcctgctttgtaacacactccggtctatcccgtagcgccgggcatatcctgtcgcaa
tgtgcaaatctcgcggcaacaaccagtgaatacttcattcacaagcctcaccgcctgatcgcggcagaaactggttatagccaatcaaccgtcgttcgtgcattccgtg
aagctgtaaacaaaggtattctgtctgtagagattgttatcggcgatcaccgtgaacgtcgcgctaacctgtaccggtttacaccatcctttttggccttcgcacaacaagc
caaaaatgcgctgatagaaagcaaattaaagatttcGtcagcggcaaccaaggttaaagctgttctcgctaagacattggctttatttaatttttttatccacacccccatgt
caaaatgatacccccctcccccctgtcaggatgacgtggcaataaagaataagaagtcacaagttaaaaaaacaaaaagatcagtttccggcggtgccggaacaacc
agcctcaaaaaattgacttcatggatcgctaaggcaaaagcaaaggctgacaatctgcggttatccaaaaaacgcactcaaaaacatgagttcaagcagaaagta
gaggcggctgcgcggaaatatgcttacctgaaAacaagcgttcgcctgatattggcgggatatcaaacttcgataacctaccgcattgcatgacggtaaacgaagc
tcttaatgcggttttagccaaaaaataaagataacgaacaatggggtataccggcaggattcagagggtaataaTACTAGAGCCAGGCATCAAATAAA
ACGAAAGGCTCAGTCGAAAGACTGGGCCTTTCGTTTTATCTGTTGTTTGTCGGTGAACGCTCTCTACTAGAGTCA
CACTGGCTCACCTTCGGGTGGGCCTTTCTGCGTTTATATACTAGTAGCGGCCGCTGCA

## References


Tridgett, M., Ababi, M., Osgerby, A., Ramirez Garcia, R. and Jaramillo, A. (2021) Engineering bacteria to produce pure phage-like particles for gene delivery. ACS Synth. Biol. *10*, 107-114.

Huan, Y.W., Fa-arun, J. and Wang, B. (2022) The Role of O-antigen in P1 Transduction of *Shigella flexneri* and *Escherichia coli* with its Alternative S' Tail Fibre. J. Mol. Biol. *434*, 167829.
